# Nitrate radicals and biogenic volatile organic compounds: oxidation, mechanisms, and organic aerosol

**DOI:** 10.5194/acp-17-2103-2017

**Published:** 2017

**Authors:** Nga Lee Ng, Steven S. Brown, Alexander T. Archibald, Elliot Atlas, Ronald C. Cohen, John N. Crowley, Douglas A. Day, Neil M. Donahue, Juliane L. Fry, Hendrik Fuchs, Robert J. Griffin, Marcelo I. Guzman, Hartmut Herrmann, Alma Hodzic, Yoshiteru Iinuma, José L. Jimenez, Astrid Kiendler-Scharr, Ben H. Lee, Deborah J. Luecken, Jingqiu Mao, Robert McLaren, Anke Mutzel, Hans D. Osthoff, Bin Ouyang, Benedicte Picquet-Varrault, Ulrich Platt, Havala O. T. Pye, Yinon Rudich, Rebecca H. Schwantes, Manabu Shiraiwa, Jochen Stutz, Joel A. Thornton, Andreas Tilgner, Brent J. Williams, Rahul A. Zaveri

**Affiliations:** 1 School of Chemical and Biomolecular Engineering, Georgia Institute of Technology, Atlanta, GA, USA; 2 School of Earth and Atmospheric Sciences, Georgia Institute of Technology, Atlanta, GA, USA; 3 NOAA Earth System Research Laboratory, Chemical Sciences Division, Boulder, CO, USA; 4 Department of Chemistry and Biochemistry, University of Colorado, Boulder, CO, USA; 5 National Centre for Atmospheric Science, University of Cambridge, Cambridge, UK; 6 Department of Atmospheric Sciences, RSMAS, University of Miami, Miami, FL, USA; 7 Department of Chemistry, University of California at Berkeley, Berkeley, CA, USA; 8 Max-Planck-Institut für Chemie, Division of Atmospheric Chemistry, Mainz, Germany; 9 Cooperative Institute for Research in Environmental Sciences, University of Colorado, Boulder, CO, USA; 10 Center for Atmospheric Particle Studies, Carnegie Mellon University, Pittsburgh, PA, USA; 11 Department of Chemistry, Reed College, Portland, OR, USA; 12 Institut für Energie und Klimaforschung: Troposphäre (IEK-8), Forschungszentrum Jülich, Jülich, Germany; 13 Department of Civil and Environmental Engineering, Rice University, Houston, TX, USA; 14 Department of Chemistry, University of Kentucky, Lexington, KY, USA; 15 Atmospheric Chemistry Department, Leibniz Institute for Tropospheric Research, Leipzig, Germany; 16 Atmospheric Chemistry Observations and Modeling, National Center for Atmospheric Research, Boulder, CO, USA; 17 Department of Atmospheric Sciences, University of Washington, Seattle, WA, USA; 18 National Exposure Research Laboratory, US Environmental Protection Agency, Research Triangle Park, NC, USA; 19 Program in Atmospheric and Oceanic Sciences, Princeton University, Princeton, NJ, USA; 20 Geophysical Fluid Dynamics Laboratory/National Oceanic and Atmospheric Administration, Princeton, NJ, USA; 21 Centre for Atmospheric Chemistry, York University, Toronto, Ontario, Canada; 22 Department of Chemistry, University of Calgary, Calgary, Alberta, Canada; 23 Department of Chemistry, University of Cambridge, Cambridge, UK; 24 Laboratoire Interuniversitaire des Systemes Atmospheriques (LISA), CNRS, Universities of Paris-Est Créteil and ì Paris Diderot, Institut Pierre Simon Laplace (IPSL), Créteil, France; 25 Institute of Environmental Physics, University of Heidelberg, Heidelberg, Germany; 26 Department of Earth and Planetary Sciences, Weizmann Institute, Rehovot, Israel; 27 Division of Geological and Planetary Sciences, California Institute of Technology, Pasadena, CA, USA; 28 Department of Chemistry, University of California Irvine, Irvine, CA, USA; 29 Department of Atmospheric and Oceanic Sciences, University of California, Los Angeles, CA, USA; 30 Department of Energy, Environmental and Chemical Engineering, Washington University in St. Louis, St. Louis, MO, USA; 31 Atmospheric Sciences and Global Change Division, Pacific Northwest National Laboratory, Richland, WA, USA

## Abstract

Oxidation of biogenic volatile organic compounds (BVOC) by the nitrate radical (NO_3_) represents one of the important interactions between anthropogenic emissions related to combustion and natural emissions from the biosphere. This interaction has been recognized for more than 3 decades, during which time a large body of research has emerged from laboratory, field, and modeling studies. NO_3_-BVOC reactions influence air quality, climate and visibility through regional and global budgets for reactive nitrogen (particularly organic nitrates), ozone, and organic aerosol. Despite its long history of research and the significance of this topic in atmospheric chemistry, a number of important uncertainties remain. These include an incomplete understanding of the rates, mechanisms, and organic aerosol yields for NO_3_-BVOC reactions, lack of constraints on the role of heterogeneous oxidative processes associated with the NO_3_ radical, the difficulty of characterizing the spatial distributions of BVOC and NO_3_ within the poorly mixed nocturnal atmosphere, and the challenge of constructing appropriate boundary layer schemes and non-photochemical mechanisms for use in state-of-the-art chemical transport and chemistry–climate models.

This review is the result of a workshop of the same title held at the Georgia Institute of Technology in June 2015. The first half of the review summarizes the current literature on NO_3_-BVOC chemistry, with a particular focus on recent advances in instrumentation and models, and in organic nitrate and secondary organic aerosol (SOA) formation chemistry. Building on this current understanding, the second half of the review outlines impacts of NO_3_-BVOC chemistry on air quality and climate, and suggests critical research needs to better constrain this interaction to improve the predictive capabilities of atmospheric models.

## 1 Introduction

The emission of hydrocarbons from the terrestrial biosphere represents a large natural input of chemically reactive compounds to Earth’s atmosphere (Guenther et al., 1995; Goldstein and Galbally, 2007). Understanding the atmospheric degradation of these species is a critical area of current research that influences models of oxidants and aerosols on regional and global scales. Nitrogen oxides (NO*_x_* = NO + NO_2_) arising from combustion and microbial action on fertilizer are one of the major anthropogenic inputs that perturb the chemistry of the atmosphere (Crutzen, 1973). Nitrogen oxides have long been understood to influence oxidation cycles of biogenic volatile organic compounds (BVOC), especially through photochemical reactions of organic and hydroperoxy radical intermediates (RO_2_ and HO_2_) with nitric oxide (NO) (Chameides, 1978).

The nitrate radical (NO_3_) arises from the oxidation of nitrogen dioxide (NO_2_) by ozone (O_3_) and occurs principally in the nighttime atmosphere due to its rapid photolysis in sunlight and its reaction with NO (Wayne et al., 1991; Brown and Stutz, 2012). The nitrate radical is a strong oxidant, reacting with a wide variety of volatile organic compounds, including alkenes, aromatics, and oxygenates as well as with reduced sulfur compounds. Reactions of NO_3_ are particularly rapid with unsaturated compounds (alkenes) (Atkinson and Arey, 2003). BVOC such as isoprene, monoterpenes, and sesquiterpenes typically have one or more unsaturated functionalities such that they are particularly susceptible to oxidation by O_3_ and NO_3_.

**Figure 1:**
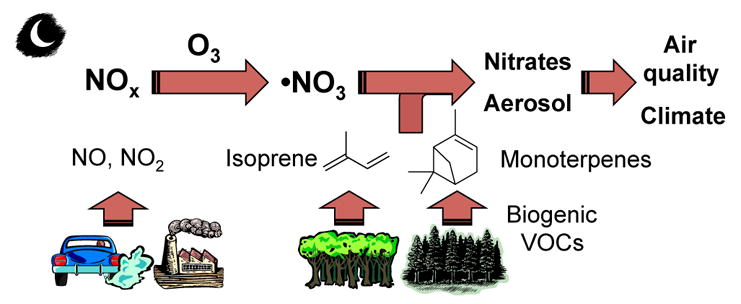
Schematic of nighttime NO_3_-BVOC chemistry.

The potential for NO_3_ to serve as a large sink for BVOC was recognized more than 3 decades ago (Winer et al., 1984). Field studies since that time have shown that in any environment with moderate to large BVOC concentrations, a majority of the NO_3_ radical oxidative reactions are with BVOC rather than VOC of anthropogenic origin (Brown and Stutz, 2012). This interaction gives rise to a mechanism that couples anthropogenic NO*_x_* emissions with natural BVOC emissions (Fry et al., 2009; Xu et al., 2015a). Although it is one of several such anthropogenic–biogenic interactions (Hoyle et al., 2011), reactions of NO_3_ with BVOC are an area of intense current interest and one whose study has proven challenging. These challenges arise from the more limited current database of laboratory data for NO_3_ oxidation reactions relative to those of other common atmospheric oxidants such as hydroxyl radical (OH) and O_3_. The mixing state of the night-time atmosphere and the limitations it imposes for characterization of nocturnal oxidation chemistry during field measurements and within atmospheric models present a second challenge to this field of research. Figure 1 illustrates these features of nighttime NO_3_-BVOC chemistry.

Reactions of NO_3_ with BVOC have received increased attention in the recent literature as a potential source of secondary organic aerosol (SOA) (Pye et al., 2010; Fry et al., 2014; Boyd et al., 2015). This SOA source is intriguing for several reasons. First, although organics are now understood to comprise a large fraction of total aerosol mass, and although much of these organics are secondary, sources of SOA remain difficult to characterize, in part due to a large number of emission sources and potential chemical mechanisms (Zhang et al., 2007; Hallquist et al., 2009; Jimenez et al., 2009; Ng et al., 2010). Analysis of aerosol organic carbon shows that a large fraction is modern, arising either from biogenic hydrocarbon emissions or biomass burning sources (e.g., Schichtel et al., 2008; Hodzic et al., 2010). Conversely, field data in regionally polluted areas indicate strong correlations between tracers of anthropogenic emissions and SOA, which suggests that anthropogenic influences can lead to production of SOA from modern (i.e., non-fossil) carbon (e.g., Weber et al., 2007). Model studies confirm that global observations are best simulated with a biogenic carbon source in the presence of anthropogenic pollutants (Spracklen et al., 2011). Reactions of NO_3_ with BVOC are one such mechanism that may lead to anthropogenically influenced biogenic SOA (Hoyle et al., 2007), and it is important to quantify the extent to which such reactions can explain sources of SOA.

Second, some laboratory and chamber studies suggest that SOA yields from NO_3_ oxidation of common BVOC, such as isoprene and selected monoterpenes, are greater than that for OH or O_3_ oxidation (Hallquist et al., 1997b; Griffin et al., 1999; Spittler et al., 2006; Ng et al., 2008; Fry et al., 2009, 2011, 2014;; Rollins et al., 2009; Boyd et al., 2015). However, among the monoterpenes, the SOA yields may be much more variable for NO_3_ oxidation than for other oxidants, with anomalously low SOA yields in some cases and high SOA yields in others (Draper et al., 2015; Nah et al., 2016b).

Third, not only is NO_3_-BVOC chemistry a potentially efficient SOA formation mechanism, it is also a major pathway for the production of organic nitrates (von Kuhlmann et al., 2004; Horowitz et al., 2007), a large component of oxidized reactive nitrogen that may serve as either a NO*_x_* reservoir or NO*_x_* sink. Results from recent field measurements have shown that organic nitrates are important components of ambient OA (Day et al., 2010; Rollins et al., 2012; Fry et al., 2013; Ayres et al., 2015; Xu et al., 2015a, b; Kiendler-Scharr et al., 2016; Lee et al., 2016). Furthermore, within the last several years, the capability to measure both total and speciated gas-phase and particle-phase organic nitrates has been demonstrated (Fry et al., 2009, 2013, 2014; Rollins et al., 2010, 2013; Lee et al., 2016; Nah et al., 2016b). The life-times of organic nitrates derived from BVOC-NO_3_ reaction with respect to hydrolysis, photooxidation, and deposition play an important role in the NO*_x_* budget and formation of O_3_ and SOA. These processes appear to depend strongly on the parent VOC and oxidation conditions and must be better constrained for understanding organic nitrate lifetimes in the atmosphere (Darer et al., 2011; Hu et al., 2011; Liu et al., 2012b; Boyd et al., 2015; Pye et al., 2015; Rindelaub et al., 2015; Lee et al., 2016; Nah et al., 2016b).

Fourth, incorporation of SOA yields for NO_3_-BVOC reactions into regional and global models indicates that these reactions could be a significant, or in some regions even dominant, SOA contributor (Hoyle et al., 2007; Pye et al., 2010, 2015; Chung et al., 2012; Fry and Sackinger, 2012; Kiendler-Scharr et al., 2016). Model predictions of organic aerosol formation from NO_3_-BVOC until recently have been difficult to verify directly from field measurements. Recent progress in laboratory and field studies have provided some of the first opportunities to develop coupled gas and particle systems to describe mechanistically and predict SOA and organic nitrate formation from NO_3_-BVOC reactions (Pye et al., 2015).

Finally, analyses from several recent field studies examining diurnal variation in the organic and/or nitrate content of aerosols conclude that nighttime BVOC oxidation through NO_3_ radicals constitutes a large organic aerosol source (Rollins et al., 2012; Fry et al., 2013; Xu et al., 2015a, b; Kiendler-Scharr et al., 2016). Although such analyses may correct their estimates of aerosol production for the variation in boundary layer depth, field measurements at surface level are necessarily limited in their ability to accurately assess the atmospheric chemistry in the overlying residual layer or even the gradients that may exist within the relatively shallow nocturnal boundary layer (Stutz et al., 2004; Brown et al., 2007b). Thus, although there is apparent consistency between recent results from both modeling and field studies, the vertically stratified structure of the nighttime atmosphere makes such comparisons difficult to evaluate critically. There is a limited database of nighttime aircraft measurements that has probed this vertical structure with sufficient chemical detail to assess NO_3_-BVOC reactions (Brown et al., 2007a; Brown et al., 2009), and some of these data show evidence for an OA source related to this chemistry, especially at low altitude (Brown et al., 2013). A larger database of aircraft and/or vertically resolved measurements is required, however, for comprehensive comparisons to model predictions.

The purpose of this article is to review the current literature on the chemistry of NO_3_ and BVOC to critically assess the current state of the science. The review focuses on BVOC emitted from terrestrial vegetation. The importance of NO_3_ reactions with reduced sulfur compounds, such as dimethyl sulfide in marine ecosystems, is well known (Platt et al., 1990; Yvon et al., 1996; Allan et al., 1999, 2000; Vrekoussis et al., 2004; Stark et al., 2007; Osthoff et al., 2009) but is outside of the scope of this review. Key uncertainties include chemical mechanisms, yields of major reaction products such as SOA and organic nitrogen, the potential for NO_3_ and BVOC to interact in the ambient atmosphere, and the implications of that interaction for current understanding of air quality and climate. The review stems from an International Global Atmospheric Chemistry (IGAC) and US National Science Foundation (NSF) sponsored workshop of the same name held in June 2015 at the Georgia Institute of Technology (Atlanta, GA, USA). Following this introduction, Sect. 2 of this article reviews the current literature in several areas relevant to the understanding of NO_3_-BVOC atmospheric chemistry. Section 3 outlines perspectives on the implications of this chemistry for understanding climate and air quality, its response to current emission trends, and its relevance to implementation of control strategies. Finally, the review concludes with an assessment of the impacts of NO_3_-BVOC reactions on air quality, visibility, and climate.

## 2 Review of current literature

This section contains a literature review of the current state of knowledge of NO_3_-BVOC chemistry with respect to (1) reaction rate constants and mechanisms from laboratory and chamber studies; (2) secondary organic aerosol yields, speciation, and particle-phase chemistry; (3) heterogeneous reactions of NO_3_ and their implications for NO_3_-BVOC chemistry; (4) instrumental methods for analysis of reactive nitrogen compounds, including NO_3_, organic nitrates, and nitrogen-containing particulate matter; (5) field observations relevant to the understanding of NO_3_ and BVOC chemistry; and (6) models of NO_3_-BVOC chemistry.

### 2.1 NO_3_-BVOC reaction rates and chemical mechanisms

#### 2.1.1 Reaction rates

Among the numerous BVOC emitted into the troposphere, kinetic data for NO_3_ oxidation have been provided for more than 40 compounds. The most emitted/important BVOC have been subject to several kinetic studies, using both absolute and relative methods, which are evaluated to determine rate constants by IUPAC (Table 1). This is the case for isoprene, *α*-pinene, *β*-pinene, and 2-methyl-3-buten-2-ol (MBO). However, for isoprene, *β*-pinene, and MBO, rate constants obtained by different studies range over a factor of 2. For some other terpenes, only few kinetic studies have been carried out, with at least one absolute rate determination. This is the case for sabinene, 2-carene, camphene, d-limonene, *α*-phellandrene, myrcene, *γ* -terpinene, and terpinolene. For these compounds, experimental data agree within 30–40 %, except *α*-phellandrene and terpinolene for which discrepancies are larger. For other BVOC, including other terpenes, sesquiterpenes, and oxygenated species, rate constants are mostly based on a single determination and highly uncertain. For these compounds, further rate constant determinations and end-product measurements are essential to better evaluate the role of NO_3_ in their degradation. The ability to predict the NO_3_-BVOC rate constants using structure–activity relationships (SARs) has been improved. A recent study (Kerdouci et al., 2010; Kerdouci, 2014) presented a new SAR parameterization based on 180 NO_3_-VOC reactions. The method is capable of predicting 90 % of the rate constants within a factor of 2.

#### 2.1.2 Mechanisms

**Figure 2:**
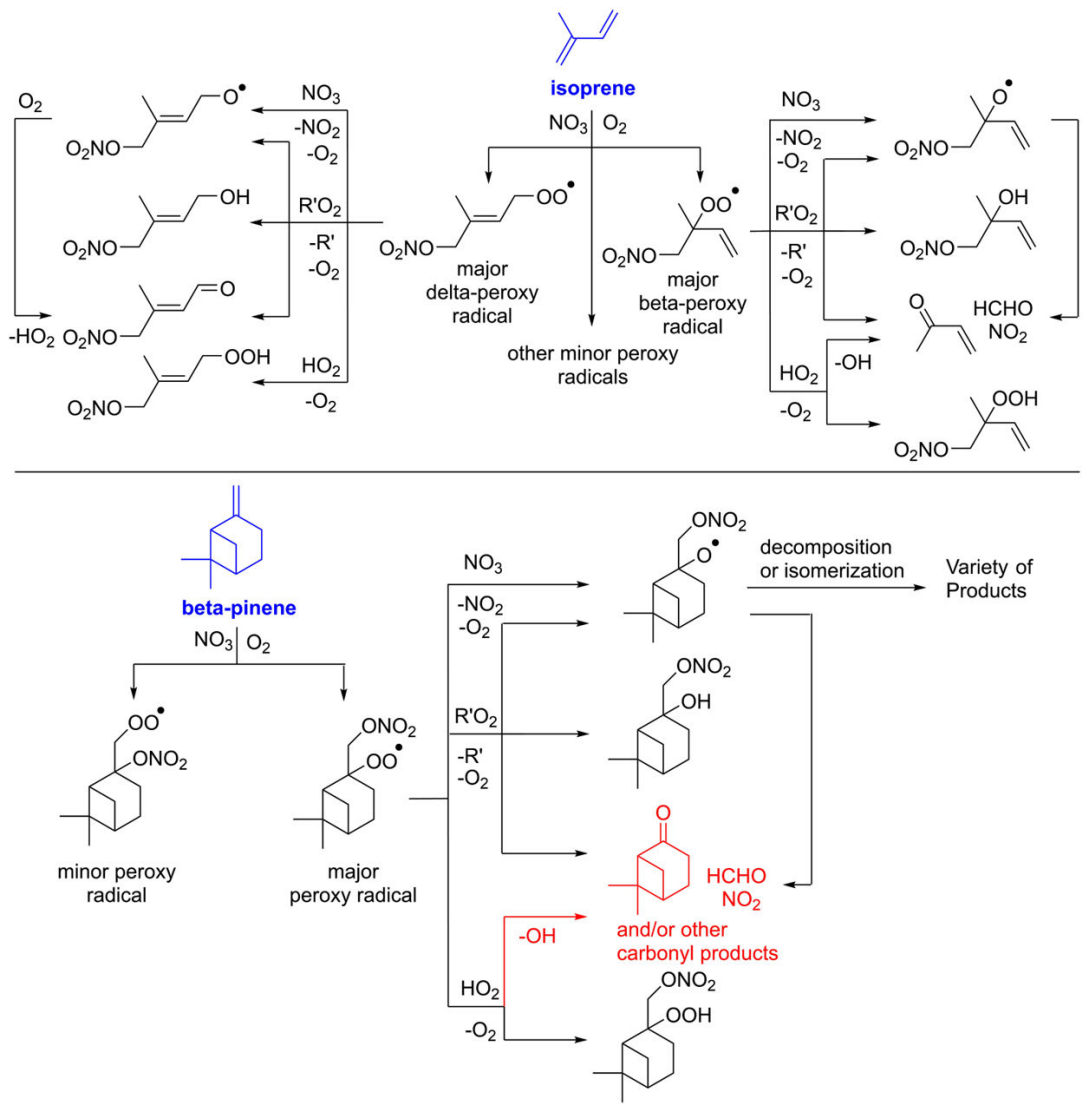
Condensed reaction mechanism for isoprene and *β*-pinene oxidation via NO_3_ (adapted from Schwantes et al., 2015 and Boyd et al., 2015). For brevity, only products generated from the dominant peroxy radicals (RO_2_) are shown. *R*′ represents an alkoxy radical, carbonyl compound, or hydroxy compound. Two of the largest uncertainties in *β*-pinene oxidation are shown in red: (1) quantification of product yields from the RO_2_+ HO_2_ channel and (2) identification of carbonyl products formed from RO_2_ reaction with NO_3_, RO_2_, or HO_2_ (see text for more details).

In general, NO_3_ reacts with unsaturated VOC by addition to a double bond (Wayne et al., 1991), though hydrogen abstraction may occur, most favorably for aldehydic species (Zhang and Morris, 2015). The location and likelihood of the NO_3_ addition to a double bond depends on the substitution on each end of the double bond, with the favored NO_3_ addition position being the one resulting in the most substituted carbon radical. In both cases, molecular oxygen adds to the resulting radical to form a peroxy radical (RO_2_). For example, the major RO_2_ isomers produced from isoprene and *β*-pinene oxidation via NO_3_ are shown in Fig. 2. The RO_2_ distribution for isoprene oxidation by OH has been shown to be dependent on the RO_2_ lifetime (Peeters et al., 2009, 2014), but no similar theoretical studies have been conducted on the NO_3_ system. Schwantes et al. (2015) determined the RO_2_ isomer distribution at an RO_2_ lifetime of ~ 30 s for isoprene oxidation via NO_3_. More theoretical and experimental studies are needed to understand the influence of RO_2_ lifetime, which is long at night (~ 50–200 s for isoprene; Schwantes et al., 2015), on the RO_2_ isomer distribution, as this distribution influences the formation of all subsequent products (Fig. 2).

The fate of RO_2_ determines the subsequent chemistry. During the nighttime in the ambient atmosphere, RO_2_ will isomerize or react with another RO_2_, NO_3_, or HO_2_. In order to monitor RO_2_ isomerization reaction products, RO_2_ life-times must be long in laboratory studies similar to the ambient atmosphere (e.g., Peeters et al., 2009; Crounse et al., 2011). The NO_3_ plus BVOC (NO_3_+ BVOC) reaction can be a source of nighttime HO_2_ and OH radicals (Platt et al., 1990). Reaction with NO is a minor peroxy radical fate at night (Pye et al., 2015; Xiong et al., 2015). Few laboratory studies have contrasted the fates of RO_2_ and their impacts on gas-phase oxidation and aerosol formation (Ng et al., 2008; Boyd et al., 2015; Schwantes et al., 2015). Boyd et al. (2015) examined how RO_2_ fate influences SOA formation and yields, and studied the competition between the RO_2_-NO_3_ and RO_2_-HO_2_ channels for *β*-pinene. Boyd et al. (2015) determined that the SOA yields for both channels are comparable, indicating that the volatility distribution of products may not be very different for the different RO_2_ fates. In contrast, the results from NO_3_ oxidation of smaller BVOC, such as isoprene, show large differences in SOA yields depending on the RO_2_ fate (Ng et al., 2008), with larger SOA yields for second-generation NO_3_ oxidation (Rollins et al., 2009).

The well-established gas-phase first-generation products from the major *β*- and *δ*-RO_2_ isomers formed from isoprene oxidation are shown in Fig. 2 (adapted from Schwantes et al., 2015). Some of the products are common between all the pathways, such as methyl vinyl ketone for the dominant *β*-RO_2_ isomer. However, some products are unique to only one channel (e.g., hydroxy nitrates form from RO_2_-RO_2_ reactions and nitrooxy hydroperoxides form from RO_2_-HO_2_ reactions). In this case, the overall nitrate yield and the specific nitrates formed from isoprene depend on the initial RO_2_ isomer distribution and the fate of the RO_2_. Furthermore, the distribution of gas-phase products will then influence the formation of SOA. For isoprene, the SOA yields from RO_2_-RO_2_ reactions are ~ 2 times greater than the yield from RO_2_-NO_3_ reactions (Ng et al., 2008). The less well-established first-generation products from *β*-pinene oxidation are also shown in Fig. 2 (adapted from Boyd et al., 2015). There are still lingering uncertainties (shown in red) in the first-generation products formed from *β*-pinene oxidation. The product yields from the RO_2_+ HO_2_ channel are not well constrained, largely due to the unavailability of authentic standards. In Fig. 2, a carbonyl product is assumed to form directly from the RO_2_ + HO_2_ reaction instead of proceeding through an alkoxy intermediate consistent with theoretical calculations from different compounds (Hou et al., 2005a, b; Praske et al., 2015). This is also uncertain, as few theoretical studies have been conducted on large molecules like *β*-pinene. The identification of the carbonyl compound(s) produced from RO_2_ reaction with NO_3_, RO_2_, or HO_2_ is unknown. Hallquist et al. (1999) detected a low molar yield (0–2 %; Table 2) of nopinone from *β*-pinene NO_3_ oxidation. Further laboratory studies identifying other carbonyl products are recommended.

Given the limited number of studies that have considered the fate of the peroxy radical, generalizations cannot yet be made for all VOC. Indeed, more studies are needed to determine systematically how gas-phase products and SOA yields are influenced by reactions of RO_2_. More specifically, for all chamber experiments, constraining the fate and lifetime of RO_2_ is required to attribute product and SOA yields to a specific pathway. As shown in Table 2 in Sect. 2.2, the nitrate yields and SOA yields for NO_3_-induced degradation of many VOC vary significantly between different studies. This is likely, in part, a result of each experiment having a different distribution of RO_2_ fates, but may also arise from vapor wall losses.

In general, there are very few mechanistic studies for NO_3_ relative to other oxidants. Furthermore, the elucidation of mechanisms is limited by the fact that most studies provide overall yields of organic nitrates (without individual identification of the species) and/or identification (without quantification) due to the lack of standards.

### 2.2 Organic aerosol yields, speciation, and particle-phase chemistry

Several papers have reported chamber studies to measure the organic aerosol yield and/or gaseous and aerosol-phase oxidation product distribution from NO_3_-BVOC reactions. These are summarized in Table 2. In general, these experimental results show that monoterpenes are efficient sources of SOA, with reported yields variable but consistently above 20 %, with the notable exception of *α*-pinene (yields 0–15 %). This anomalous monoterpene also has a much larger product yield of carbonyls instead of organic nitrates compared to the others. This difference among monoterpenes was investigated in the context of the competition between O_3_ and NO_3_ oxidation (Draper et al., 2015), in which shifting from O_3_-dominated to NO_3_-dominated oxidation was observed to suppress SOA formation from *α*-pinene, but not from *β*-pinene, Δ-carene, or limonene. The smaller isoprene has substantially lower SOA yields (2–24 %), and the only sesquiterpene studied, *β*-caryophyllene, has a much larger yield (86–150 %) than the monoterpenes.

In general, these chamber experiments are conducted under conditions that focus on first-generation oxidation only, but further oxidation can continue to change SOA loadings in the real atmosphere (e.g., Rollins et al., 2009; Chacon-Madrid et al., 2013). Recent experiments showed that particulate organic nitrates formed from *β*-pinene-NO_3_ are resilient to photochemical aging, while those formed from *α*-pinene-NO_3_ evaporate more readily (Nah et al., 2016b).

Other chamber studies have not reported SOA mass yields or gas-phase product measurements but have otherwise demonstrated the importance of NO_3_-BVOC reactions to SOA production. These studies have identified *β*-pinene and Δ-carene as particularly efficient sources of SOA upon NO_3_ oxidation (Hoffmann et al., 1997), confirmed the greater aerosol-forming potential from *β*-pinene versus *α*-pinene (Bonn and Moortgat, 2002), and reported Fourier transform infrared spectroscopy (FTIR) and aerosol mass spectrometry (AMS) measurements of the composition of organic nitrates detected in aerosol formed from NO_3_-isoprene, *α*-pinene, *β*-pinene, Δ-carene, and limonene reactions (Bruns et al., 2010).

Relative humidity (RH) can be an important parameter, as it affects the competition between NO_3_-BVOC reactions and heterogeneous uptake of N_2_O_5_. Among existing laboratory studies, only a few have focused on the effect of RH on SOA formation from NO_3_-initiated oxidation (Bonn and Moortgat, 2002; Spittler et al., 2006; Fry et al., 2009; Boyd et al., 2015). The impact of RH might be important, especially at night and during the early morning when RH near the surface is high and NO_3_ radical chemistry is competitive with O_3_ and OH reactions. However, observations of the effect of water on SOA formation originating from NO_3_ oxidation hint at a varied role. Spittler et al. (2006) reported lower SOA yields under humid conditions, but other studies did not observe a significant effect (Bonn and Moortgat, 2002; Fry et al., 2009; Boyd et al., 2015). Among the important effects of water is its role as a medium for hydrolysis. In laboratory studies, primary and secondary organic nitrates were found to be less prone to aqueous hydrolysis than tertiary organic nitrates (Darer et al., 2011; Hu et al., 2011). First-generation organic nitrates retaining double bonds may also hydrolyze relatively quickly, especially in the presence of acidity (Jacobs et al., 2014; Rindelaub et al., 2015). Depending on the relative amount of these different types of organic nitrates, the overall hydrolysis rate could be different for organic nitrates formed from NO_3_ oxidation and photooxidation in the presence of NO*_x_* (Boyd et al., 2015). Recently, there has been increasing evidence from field measurements that organic nitrates hydrolyze in the particle phase, producing HNO_3_ (Liu et al., 2012b; Browne et al., 2013). This has been only a limited focus of chamber experiments to date (Boyd et al., 2015). In addition to the effect of RH, particle-phase acidity is known to affect SOA formation from ozonolysis and OH reaction (e.g., Gao et al., 2004; Tolocka et al., 2004). Thus far, only one study has examined the effect of acidity on NO_3_-initiated SOA formation and found a negligible effect (Boyd et al., 2015). Notably, an effect of acidity was observed for the hydrolysis of organic nitrates produced in photochemical reactions (Szmigielski et al., 2010; Rindelaub et al., 2015). While much organic nitrate aerosol is formed via NO_3_+ BVOC reactions, some fraction can also form from RO_2_+ NO chemistry. Rollins et al. (2010) observed the organic nitrate moiety in 6–15 % of total SOA mass generated from high-NO*_x_* photooxidation of limonene, *α*-pinene, Δ^3^-carene, and tridecane. A very recent study of Berkemeier et al. (2016) showed that organic nitrates accounted for ~ 40 % of SOA mass during initial particle formation in *α*-pinene oxidation by O_3_ in the presence of NO, decreasing to ~ 15 % upon particle growth to the accumulation-mode size range. They also observed a tight correlation (*R*^2^ = 0.98) between organic nitrate content and SOA particle number concentrations. This implies that organic nitrates may be among the extremely low volatility organic compounds (ELVOC) (Ehn et al., 2014; Tröstl et al., 2016) that play a critical role in nucleation and nanoparticle growth.

### 2.3 Heterogeneous and aqueous-phase NO_3_ processes

The NO_3_ radical is not only a key nighttime oxidant of organic (and especially biogenic) trace gases but it can also play an important role in the aqueous phase of tropospheric clouds and deliquesced particles (Chameides, 1978; Wayne et al., 1991; Herrmann and Zellner, 1998; Rudich et al., 1998). Whilst the reaction of NO_3_ with organic particles and aqueous droplets in the atmosphere is believed to represent only an insignificant fraction of the overall loss rate for NO_3_, it can have a substantial impact on the chemical and physical properties of the particle by modifying its lifetime, oxidation state, viscosity, and hygroscopic properties and thus its propensity to act as a cloud condensation nucleus (Rudich, 2003).

Biogenic VOC include, but are not limited to the isoprenoids (isoprene, mono-, and sesquiterpenes) as well as alkanes, alkenes, carbonyls, alcohols, esters, ethers, and acids (Kesselmeier and Staudt, 1999). Recent measurements indicate that biogenic emissions of aromatic trace gases are also significant (Misztal et al., 2015). The gas-phase degradation of BVOC leads to the formation of a complex mixture of organic trace gases including hydroxyl- and nitrate-substituted oxygenates which can transfer to the particle phase by condensation or dissolution. Our present understanding is that non-anthropogenic SOA has a large contribution from isoprenoid degradation.

As is generally the case for laboratory studies of heterogeneous processes, most of the experimental investigations on heterogeneous uptake of NO_3_ to organic surfaces have dealt with single-component systems that act as surrogates for the considerably more complex mixtures found in atmospheric SOA. A further level of complexity arises when we consider that initially reactive systems, e.g., containing condensed or dissolved unsaturated hydrocarbons, can become deactivated as SOA ages, single bonds replace double bonds, and the oxygen-to-carbon ratio increases.

We summarize the results of the laboratory studies to provide a rough guide to NO_3_ reactivity on different classes of organics which may be present in SOA and note that further studies of NO_3_ uptake to biogenic SOA which was either generated and aged under well-defined conditions (Fry et al., 2011) or sampled from the atmosphere are required to confirm predictions of uptake efficiency based on the presently available database.

#### 2.3.1 Heterogeneous processes

For some particle-phase organics, the reaction with NO_3_ is at least as important as other atmospheric oxidants such as O_3_ and OH (Shiraiwa et al., 2009; Kaiser et al., 2011). The lifetime (*τ*) of a single component, liquid organic particle with respect to loss by reaction with NO_3_ at concentration [NO_3_] is partially governed by the uptake coefficient (*γ*) (Robinson et al., 2006; Gross et al., 2009):

(1) $$\tau_{\text{liquid}}=\frac{2\rho_{\text{org}}N_{A}D_{p}}{3M_{\text{org}}\bar{c}\gamma [{\text{NO}}_{3}]},$$

where *D*_p_ is the particle diameter, *ρ*_org_ and *M*_org_ are the density and molecular weight of the organic component, respectively, *c̄* is the mean molecular velocity of gas-phase NO_3_, and N_A_ is Avogadro number. Thus, defined, *τ* is the time required for all the organic molecules in a spherical (i.e., liquid) particle to undergo a single reaction with NO_3_.

Recent studies have shown that organic aerosols can adopt semi-solid (highly viscous) or amorphous solid (crystalline or glass) phase states, depending on the composition and ambient conditions (Virtanen et al., 2010; Koop et al., 2011; Renbaum-Wolff et al., 2013). Typically, the bulk phase diffusion coefficients of NO_3_ are ~ 10^−7^–10^−9^ cm^2^ s^−1^ in semi-solid and ~ 10^−10^ cm^2^ s^−1^ in solids (Shiraiwa et al., 2011). Slow bulk diffusion of NO_3_ in a viscous organic matrix can effectively limit the rate of uptake (Xiao and Bertram, 2011; Shiraiwa et al., 2012). Similarly, the solubility may be different in a concentrated, organic medium. If bulk diffusion is slow, the reaction may be confined to the near-surface layers of the particle or bulk substrate. The presence of organic coatings on aqueous aerosols was found to suppress heterogeneous N_2_O_5_ hydrolysis by providing a barrier through which N_2_O_5_ needs to diffuse to undergo hydrolysis (Alvarado et al., 1998; Cosman et al., 2008; Grifiths et al., 2009). Reactive uptake by organic aerosols is expected to exhibit a pronounced decrease at low RH and temperature, owing to a phase transition from viscous liquid to semi-solid or amorphous solid (Arangio et al., 2015). Therefore, the presence of a semi-solid matrix may effectively shield reactive organic compounds from chemical degradation in long-range transport in the free troposphere.

**Figure 3:**
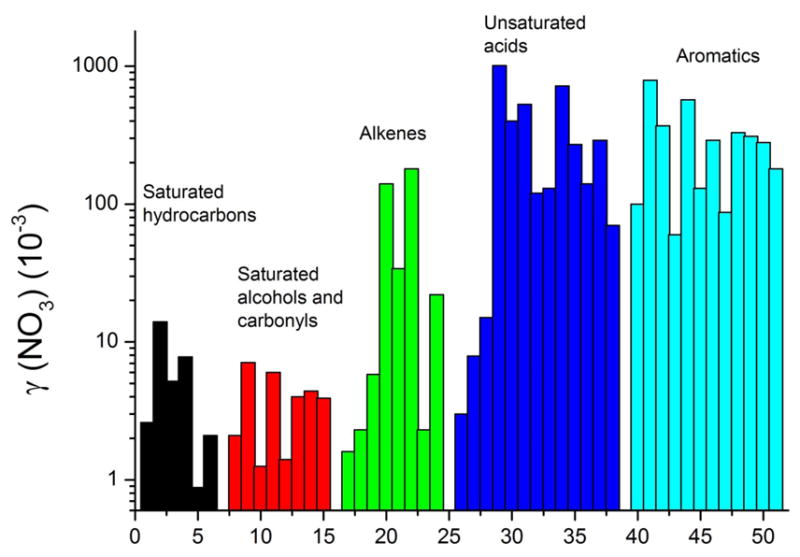
Uptake coefficients, *γ* (NO_3_), for the interaction of NO_3_ with single-component organic surfaces. Details of the experiments and the references (corresponding to the *x*-axis numbers) are given in Table S1 in the Supplement.

To get an estimate of the processing rate of BVOC-derived SOA, we have summarized the results of several laboratory studies to provide a rough guide to NO_3_ reactivity on different classes of organics that may be present in SOA (Fig. 3). A rough estimate of the reactivity of NO_3_ to freshly generated, isoprenoid-derived SOA, which still contains organics with double bonds (e.g., from diolefinic monoterpenes such as limonene), may be obtained by considering the data on alkenes and unsaturated acids, where the uptake coefficient is generally close to 0.1.

The classes of organics for which heterogeneous reactions with NO_3_ have been examined are alkanoic/alkenoic acids, alkanes and alkenes, alcohols, aldehydes, polyaromatic hydrocarbons (PAHs), and secondary organic aerosols. Laboratory studies have used either pure organic substrates, with the organic of interest internally mixed in an aqueous particle; as a surface coating, with the reactive organic mixed in a nonreactive organic matrix; or in the form of self-assembling monolayers. The surrogate surface may be available as a macroscopic bulk liquid (or frozen liquid) or in particulate form and both gas-phase and particle-phase analyses have been used to derive kinetic parameters and investigate products formed.

In the gas phase, the NO_3_ radical reacts slowly (by H-abstraction) with alkanes, more rapidly with aldehydes due to the weaker C-H bond of the carbonyl group, and most readily with alkenes and aromatics via electrophilic addition. This trend in reactivity is also observed in the condensed-phase reactions of NO_3_ with organics so that long-chain organics, for which non-sterically hindered addition to a double bond is possible, and aromatics are the most reactive. In very general terms, uptake coefficients are in the range of 1–10 × 10^−3^ for alkanes, alcohols, and acids without double bonds, 2–200 × 10^−3^ for alkenes with varying numbers of double bonds, 3–1000 × 10^−3^ for acids with double bonds again depending on the number of double bonds, and 100–500 × 10^−3^ for aromatics. These trends are illustrated in Fig. 3 which plots the experimental data for the uptake of NO_3_ to single-component organic surfaces belonging to different classes of condensable organics. Condensed-phase organic nitrates have been frequently observed following interaction of NO_3_ with organic surfaces (see below).

##### Saturated hydrocarbons

Uptake of NO_3_ to saturated hydrocarbons is relatively slow, with uptake coefficients close to 10^−3^. Moise et al. (2002) found that (for a solid sample) uptake to a branched-chain alkane was more efficient than for a straight-chain alkane, which is consistent with known trends in gas-phase reactivity of NO_3_. The slow surface reaction with alkanes enables both surface and bulk components of the reaction to operate in parallel. The observation of RONO_2_ as product is explained (Knopf et al., 2006; Gross and Bertram, 2009) by processes similar to those proceeding in the gas phase, i.e., abstraction followed by formation of peroxy and alkoxy intermediates which react with NO_2_ and NO_3_ to form the organic nitrate.

##### Unsaturated hydrocarbons

With exception of the data of Moise et al. (2002), the up-take of NO_3_ to an unsaturated organic surface is found to be much more efficient than to the saturated analogue. The NO_3_ uptake coefficient for, e.g., squalene, is at least an order of magnitude more efficient than for squalane (Xiao and Bertram, 2011; Lee et al., 2013). The location of the double bond is also important and the larger value for *γ* found for a self-assembling monolayer of NO_3_+ undec-10-ene-1-thiol compared to liquid, long-chain alkenes is due to the fact that the terminal double bond is located at the interface and is thus more accessible for a gas-phase reactant (Gross and Bertram, 2009). NO_3_ uptake to mixtures of unsaturated methyl oleate in a matrix of saturated organic was found to be consistent with either a surface or bulk reaction (Xiao and Bertram, 2011). The formation of condensed-phase organic nitrates and simultaneous loss of the vinyl group indicates that the reaction proceeds, as in the gas phase, by addition of NO_3_ to the double bond followed by reaction of NO_3_ (or NO_2_) with the resulting alkyl and peroxy radicals formed (Zhang et al., 2014b).

##### Saturated alcohols and carbonyls

Consistent with reactivity trends for NO_3_ in the gas phase, the weakening of some C-H bonds in oxidized, saturated organics results in a more efficient interaction of NO_3_ than for the non-oxidized counterparts although, as far as the limited dataset allows trends to be deduced, the gas-phase reactivity trend of polyalcohol being greater than alkanoate appears to be reversed in the liquid phase (Gross et al., 2009). For multicomponent liquid particles, the uptake coefficient will also depend on the particle viscosity (Iannone et al., 2011) though it has not been clearly established if the reaction proceeds predominantly at the surface or throughout the particle (Iannone et al., 2011). The reaction products are expected to be formed via similar pathways as seen in the gas phase, i.e., abstraction of the aldehydic-H atom for aldehydes and abstraction of an H atom from either the O-H or adjacent *α*-CH_2_ group for alcohols prior to reaction of NO_2_ and NO_3_ with the ensuing alkyl and peroxy radicals (Zhang and Morris, 2015).

##### Organic acids

The efficiency of uptake of NO_3_ to unsaturated acids is comparable to that found with other oxidized, saturated organics (Moise et al., 2002) suggesting that the reaction proceeds, as in the gas phase, via abstraction rather than addition. Significantly larger uptake coefficients have been observed for a range of unsaturated, long-chain acids, with *γ* often between 0.1 and 1 (Gross et al., 2009; Knopf et al., 2011; Zhao et al., 2011a). *γ* depends on the number and position (steric factors) of the double bond. For example, the uptake coefficient for abietic acid is a factor of 100 lower than for linoleic acid (Knopf et al., 2011). The condensed-phase products formed in the interaction of NO_3_ with unsaturated acids are substituted carboxylic acids, including hydroxy nitrates, carbonyl nitrates, dinitrates, and hydroxy dinitrates (Hung et al., 2005; Docherty and Ziemann, 2006; McNeill et al., 2007; Zhao et al., 2011a).

##### Aromatics

The interaction of NO_3_ with condensed-phase aromatics and PAHs results in the formation of a large number of nitrated aromatics and nitro PAHs. Similar to the gas-phase mechanism, the reaction is initiated by addition of NO_3_ to the aromatic ring, followed by breaking of an N-O bond to release NO_2_ to the gas phase and forming a nitrooxy-cyclohexadienyl-type radical which can further react with O_2_, NO_2_, or undergo internal rearrangement to form hydroxyl species (Gross and Bertram, 2008; Lu et al., 2011). The uptake coefficients are large and comparable to those derived for the unsaturated fatty acids.

The literature results on the interaction of NO_3_ with organic substrates are tabulated in Table S1 in the Supplement, in which the uptake coefficient is listed (if available) along with the observed condensed- and gas-phase products.

#### 2.3.2 Aqueous-phase reactions

The in situ formation of NO_3_ (e.g., electron transfer reactions between nitrate anions and other aqueous radical anions such as ${\text{SO}}_{x}^{-}$, sulfur-containing radical anions, or ${\text{Cl}}_{2}^{-}$) is generally of minor importance and the presence of NO_3_ in aqueous particles is largely a result of transfer from the gas phase (Herrmann et al., 2005; Tilgner et al., 2013). Concentrations of NO_3_ in tropospheric aqueous solutions cannot be measured in situ, and literature values are based on multiphase model predictions (Herrmann et al., 2010). Model studies with the chemical aqueous-phase radical mechanism (CAPRAM; Herrmann et al., 2005; Tilgner et al., 2013) predict [NO_3_] between 1 × 6 × 10^−16^ and 2.7 × 10^−13^ mol L^−1^. High NO_3_ concentration levels are associated with urban clouds, while in rural and marine clouds these levels are an order of magnitude lower. Since the NO_3_ concentrations are related to the NO*_x_* budget, typically higher NO_3_ concentrations are present under urban cloud conditions compared to rural and marine cloud regimes.

NO_3_ radicals react with dissolved organic species via three different pathways: (i) by H-atom abstraction from saturated organic compounds, (ii) by electrophilic addition to double bonds within unsaturated organic compounds, and (iii) by electron transfer from dissociated organic acids (Huie, 1994; Herrmann and Zellner, 1998). For a detailed overview on aqueous-phase NO_3_ radical kinetics, the reader is referred to several recent summaries (Neta et al., 1988; Herrmann and Zellner, 1998; Ross et al., 1998; Herrmann, 2003; Herrmann et al., 2010, 2015). Compared to the highly reactive and non-selective OH radical, the NO_3_ radical is characterized by a lower reactivity and represents a more selective aqueous-phase oxidant. The available kinetic data indicate that the reactivity of NO_3_ radicals with organic compounds in comparison to the two other key radicals (OH, ${\text{SO}}_{4}^{-}$) is as follows: $\text{OH}>{\text{SO}}_{4}^{-}\gg {\text{NO}}_{3}$ (Herrmann et al., 2015).

In Table S2, we list kinetic parameters for reaction of NO_3_ with aliphatic organic compounds as presently incorporated in the CAPRAM database (Bräuer et al., 2017). Typical ranges of rate constants (in M^−1^ s^−1^) for reactions of NO_3_ in the aqueous phase are 106–107 for saturated alcohols, carbonyls, and sugars; 10^4^–10^6^ for protonated aliphatic mono- and dicarboxylic acids, with higher values for oxygenated acids; 10^6^–10^8^ for deprotonated aliphatic mono- and dicarboxylic acids (higher values typically for oxygenated acids); 10^7^–10^9^ for unsaturated aliphatic compounds; and 10^8^–2 × 10^9^ for aromatic compounds (without nitro/acid functionality). The somewhat larger rate constants for deprotonated aliphatic mono- and dicarboxylic acids, unsaturated aliphatic compounds and aromatic compounds is related to the occurrence of electron transfer reactions and addition reaction pathways, which are often faster than H-abstraction reactions.

Many aqueous-phase NO_3_ reaction rate constants, even for small oxygenated organic compounds, are not available in the literature and have to be estimated. In the absence of SARs for NO_3_ radical reactions with organic compounds, Evans–Polanyi-type reactivity correlations are used to predict kinetic data for H-abstraction NO_3_ radical reactions. The latest correlation for NO_3_ reactions in aqueous solution based on 38 H-abstraction reactions of aliphatic alcohols, carbonyl compounds and carboxylic acids was published by Hoffmann et al. (2009):

(2) $$\log(k_{H})=(39.9\pm 5.4)-(0.087\pm 0.014)\times \text{BDE},$$

where BDE is the bond dissociation energy (in kJ mol^−1^). The correlation is quite tight, with a correlation coefficient of *R* = 0.9.

**Figure 4:**
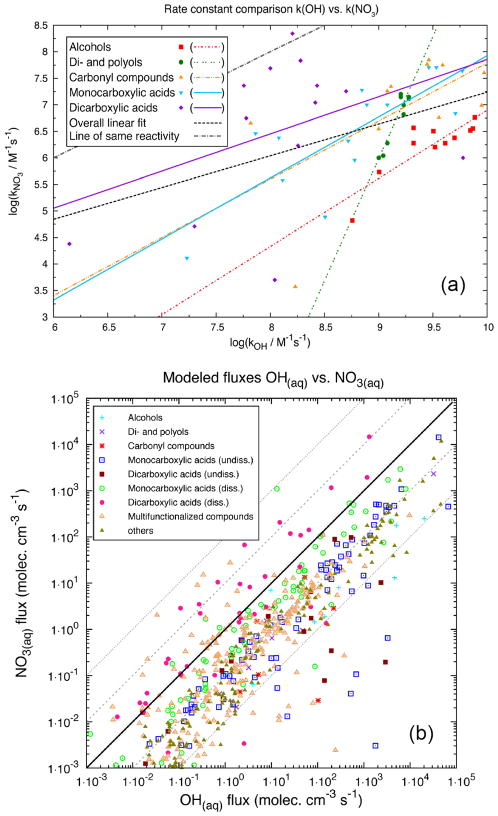
**(a)** Correlation of OH versus NO_3_ radical rate constants in the aqueous phase for the respective compound classes. The linear regression fits for the different compound classes are presented in the same color as the respective data points. The black line represents the correlation of the overall data. **(b)** Comparison of modeled, aqueous-phase reaction fluxes (mean chemical fluxes in mol cm^−3^ s^−1^ over a simulation period of 4–5 days) of organic compounds with hydroxyl (OH) versus nitrate (NO_3_) radicals distinguished by different compound classes (urban CAPRAM summer scenario).

A direct comparison of the aqueous-phase OH and NO_3_ radical rate constants (*k*_298 K_) of organic compounds from different compound classes is presented in Fig. 4, which shows that the NO_3_ radical reaction rate constants for many organic compounds are about 2 orders of magnitude smaller than respective OH rate constants. In contrast, deprotonated dicarboxylic acids can react with NO_3_ via electron transfer and have similar rate constants for OH reaction. Rate constants for OH and NO_3_ with alcohols and diols/polyols are well correlated (*R*^2^ values are given in Table S3), whereas those rate constants for carbonyl compounds and diacids have a lower degree of correlation.

Figure 4b shows a comparison of the modeled chemical turnovers of reactions of organic compounds with OH versus NO_3_ radicals distinguished for different compound classes. The simulations were performed with the SPACCIM model (Wolke et al., 2005) for the urban summer CAPRAM scenario (see Tilgner et al., 2013 for details) using the master chemical mechanism (MCM) 3.2/CAPRAM 4.0 mechanism (Rickard, 2015; Bräuer et al., 2017) which has in total 862 NO_3_ radical reactions with organic compounds.

Most of the data lie under the 1 : 1 line, indicating that, for most of the organic compounds considered, chemical degradation by OH is more important than by NO_3_, with a significant fraction of the data lying close to a 10 : 1 line, though OH fluxes sometimes exceed NO_3_ fluxes by a factor of 10^3^ – 10^4^. Approximate relative flux ratios (NO_3_ / OH) for different classes of organic are 10^−1^ – 10^−2^ for alcohols (including diols and polyols) and carbonyl compounds, 10^−1^ – 10^−4^ for undissociated monoacids and diacids, ~ 1 (or larger) for dissociated monoacids, 10^−2^ – > 10 for dissociated diacids, and 10^−2^–1 for organic nitrates. For carboxylate ions, NO_3_-initiated electron transfer is thus the dominant oxidation pathway. As OH-initiated oxidation proceeds via an H-abstraction, high NO_3_-OH flux ratios can be observed for carboxylate ions but not for protonated carboxylic acids.

Overall, Fig. 4b shows that, over a 4-day summer cycle, NO_3_ radical reactions can compete with OH radical reactions in particular for protonated carboxylic acids and multifunctional compounds. Nevertheless, aqueous NO_3_ radical reactions with organics will become more important during winter or at higher latitudes, where photochemistry as the main source of OH is less important. Finally, it should be noted that NO_3_ aqueous-phase nighttime chemistry will influence the concentration levels of many aqueous-phase reactants available for reaction during the next day.

### 2.4 Instrumental methods

Atmospheric models of the interaction of NO_3_ with BVOC rely on experimental data gathered in both the laboratory and the field. These experimental data are used to define model parameters and to evaluate model performance by comparison to observed quantities. Instrumentation for measurements of nitrogen-containing species, oxidants, and organic compounds, including NO*_x_*, O_3_, NO_3_, BVOC, and oxidized reactive nitrogen compounds, are all important to understand the processes at work. Of particular importance to the subject of this review is the characterization of organic nitrates, which are now known to exist in both the gas and particle phases and whose atmospheric chemistry is complex. This section reviews historical and current experimental methods used for elucidating NO_3_-BVOC atmospheric chemistry.

#### 2.4.1 Nitrate radical measurements

Optical absorption spectroscopy has been the primary measurement technique for NO_3_. It usually makes use of two prominent absorption features of NO_3_ near 623 and 662 nm. Note that the dissociation limit of the NO_3_ molecule lies between the two absorption lines (Johnston et al., 1996); thus, illumination by measurement radiation at the longer wave-length band does not lead to photolysis of NO_3_. The room temperature absorption cross section of NO_3_ at 662 nm is ~ 2 × 10^−17^ cm^2^ molec^−1^ and increases at lower temperature (Yokelson et al., 1994; Osthoff et al., 2007). Thus, at a typical minimum detectable optical density (reduction of the intensity compared to no absorption) and a light-path length of 5 km, a detection limit of 10^7^ molec cm^−3^ or ~ 0.4 ppt (under standard conditions) is achieved.

Initial measurements of NO_3_ in the atmosphere were long-path averages using light paths between either the sun or the moon (e.g., Noxon et al., 1978) and the receiving spectrometer (also called passive techniques because natural light sources were used) or between an artificial light source and spectrometer over a distance of several kilometers (active techniques; e.g., Platt et al., 1980). Passive techniques were later extended to yield NO_3_ vertical profiles (e.g., Weaver et al., 1996). In recent years, resonator cavity techniques allowed construction of very compact instruments capable of performing in situ measurements of NO_3_ with absorption spectroscopy (see in situ measurement techniques below).

An important distinction between the techniques is whether NO_3_ can be deliberately or inadvertently removed from the absorption path as part of the observing strategy. Long-path absorption spectroscopy does not allow control over the sample for obtaining a zero background by removing NO_3_ (Category 1). Resonator techniques (at least as long as the resonator is encased) allow deliberate removal of NO_3_ from the absorption path as part of the measurement sequence and may also result in inadvertent removal during sampling (Category 2).

For instruments of Category 1, the intensity without absorber (I_0_) cannot be easily detected. Therefore, the information about the absorption due to NO_3_ (and any other trace gas) has to be determined from the structure of the absorption, which is usually done by using differential optical absorption spectroscopy (DOAS) (Platt and Stutz, 2008), which relies on the characteristic fingerprint of the NO_3_ absorption structure in a finite wavelength range (about several 10 nm wide). Thus, a spectrometer of sufficient spectral range and resolution (around 0.5 nm) is required.

Instruments of Category 2 can determine the NO_3_ concentration from the difference (or rather log of the ratio) of the intensity with and without NO_3_ in the measurement volume. In this case, only an intensity measurement at a single wavelength (typically of a laser) is necessary, and specificity can be achieved through chemical titration with NO (Brown et al., 2001). However, enhanced specificity without chemical titration can be gained by combining resonator techniques with DOAS detection. It should be noted that the advantage of a closed cavity to be able to remove (or manipulate) NO_3_ comes at the expense of potential wall losses, which have to be characterized. Such instruments have the advantage of being able to also detect N_2_O_5_, which is in thermal equilibrium with NO_3_ and can be quantitatively converted to NO_3_ by thermal dissociation (Brown et al., 2001, 2002).

Another complication arises from the presence of water vapor and oxygen lines in the wavelength range of strong NO_3_ absorptions. To compensate for these potential interferences in open-path measurements (where NO_3_ cannot easily be removed), daytime measurements are frequently used as reference because NO_3_ levels are typically very low (but not necessarily negligibly low) (Geyer et al., 2003). Thus, a good fraction of the reported NO_3_ data (in particular, older data) represents day–night differences.

##### Passive long-path remote sensing techniques

Measurements of the NO_3_ absorption structure using sunlight take advantage of the fact that NO_3_ is very quickly photolyzed by sunlight (around 5 s lifetime during the day) allowing for vertically resolved measurements during twilight (e.g., Aliwell and Jones, 1998; Allan et al., 2002; Coe et al., 2002; von Friedeburg et al., 2002). The fact that the NO_3_ concentration is nearly zero due to rapid photolysis in the directly sunlit atmosphere, while it is largely undisturbed in a shadowed area, can be used to determine NO_3_ vertical concentration profiles during sunrise using the moon as a light source (Smith and Solomon, 1990; Smith et al., 1993; Weaver et al., 1996). Alternatively, the time series of the NO_3_ column density derived from scattered sunlight originating from the zenith (or from a viewing direction away from the sun) during sunrise can be evaluated to yield NO_3_ vertical profiles (Allan et al., 2002; Coe et al., 2002; von Friedeburg et al., 2002).

Nighttime NO_3_ total column data have been derived by spectroscopy of moonlight and starlight (Naudet et al., 1981), the intensity of which is about 4–5 orders of magnitude lower than that of sunlight. Thus, photolysis of NO_3_ by moonlight is negligible. A series of moonlight NO_3_ measurements have been reported (Noxon et al., 1980; Noxon, 1983; Sanders et al., 1987; Solomon et al., 1989, 1993; Aliwell and Jones, 1996a, b; Wagner et al., 2000). These measurements yield total column data of NO_3_, the sum of tropospheric and stratospheric partial columns. Separation between stratospheric and tropospheric NO_3_ can be accomplished (to some extent) by the Langley plot method (Noxon et al., 1980), which takes advantage of the different dependence of tropospheric and stratospheric NO_3_ slant column density on the lunar zenith angle.

##### Active long-path techniques

A large number of NO_3_ measurements have been made using the active long-path DOAS technique (Platt et al., 1980, 1981, 1984; Pitts et al., 1984; Heintz et al., 1996; Allan et al., 2000; Martinez et al., 2000; Geyer et al., 2001a, b, 2003; Gölz et al., 2001; Stutz et al., 2002, 2004, 2010; Asaf et al., 2009; McLaren et al., 2010; Crowley et al., 2011; Sobanski et al., 2016). Here, a searchlight-type light source is used to transmit a beam of light across a kilometer-long light path in the open atmosphere to a receiving telescope–spectrometer combination. The light source typically is a broadband thermal radiator (incandescent lamp, Xe arc lamp, laser-driven light source). More recently, LED light sources were also used (Kern et al., 2006). The telescope (around 0.2 m diameter) collects the radiation and transmits it, usually through an optical fiber, into the spectrometer, which produces the absorption spectrum. Modern instruments now almost exclusively use transmitter/receiver combinations at one end of the light path and retro-reflector arrays (e.g., cat-eye-like optical devices) at the other end. The great advantage of this approach is that power and optical adjustment is only required at one end of the light path while the other end (with the retro-reflector array) is fixed. In this way, several retro-reflector arrays, for instance, mounted at different altitudes, can be used sequentially with the same transmitter/receiver unit allowing determination of vertical profiles of NO_3_ (and other species measurable by DOAS) (Stutz et al., 2002, 2004, 2010).

##### In situ measurement techniques

Cavity ring-down spectroscopy (CRDS) and cavity-enhanced absorption spectroscopy (CEAS) are related techniques for in situ quantification of atmospheric trace gases such as NO_3_. These methods are characterized by high sensitivity, specificity, and acquisition speed (Table 3a), and they allow for spatially resolved measurements on mobile platforms.

In CRDS, laser light is “trapped” in a high-finesse stable optical cavity, which usually consists of a pair of highly reflective spherical mirrors in a near-confocal arrangement. The concentrations of the optical absorbers present within the resonator are derived from the Beer–Lambert law and the rate of light leaking from the cavity after the input beam has been switched off (O’Keefe and Deacon, 1988). CRDS instruments are inherently sensitive as they achieve long effective optical absorption paths (up to, or in some cases exceeding, 100 km) as the light decay is monitored for several 100 μs, and the absorption measurement is not affected by laser intensity fluctuations. For detection of NO_3_ at 662 nm, pulsed laser sources such as Nd:YAG pumped dye lasers have been used because of the relative ease of coupling the laser beam to the optical cavity (Brown et al., 2002, 2003; Dubé et al., 2006). Relatively lower cost continuous-wave (cw) diode laser modules that are easily modulated also have been popular choices (e.g., King et al., 2000; Simpson, 2003; Ayers et al., 2005; Odame-Ankrah and Osthoff, 2011; Wagner et al., 2011).

In a CEAS instrument (also referred to as integrated cavity output spectroscopy, ICOS, or cavity-enhanced DOAS, CE-DOAS), the spectrum transmitted through a high-finesse optical cavity is recorded. Mixing ratios of the absorbing gases are derived using spectral retrieval routines similar to those used for open-path DOAS (e.g., O’Keefe, 1998, 1999; Ball et al., 2001; Fiedler et al., 2003; Platt et al., 2009; Schuster et al., 2009).

CRDS and CEAS are, in principle, absolute measurement techniques and do not need to rely on external calibration. In practice, however, chemical losses can occur on the inner walls of the inlet (even when constructed from inert materials such as Teflon) or at the aerosol filters necessary for CRDS instruments. Hence, the inlet transmission efficiencies have to be monitored for measurements to be accurate (Fuchs et al., 2008, 2012; Odame-Ankrah and Osthoff, 2011). On the other hand, a key advantage of in situ instruments over open-path instruments is that the sampled air can be manipulated. Deliberate addition of excess NO to the instrument’s inlet titrates NO_3_ and allows measurement of the instrument’s zero level and separation of contributions to optical extinction from other species, such as NO_2_, O_3_, and H_2_O. Adding a heated section to the inlet (usually in a second detection channel) enables (parallel) detection of N_2_O_5_ via the increase in the NO_3_ signal (Brown et al., 2001; Simpson, 2003).

In addition, non-optical techniques have been used to detect and quantify NO_3_. Chemical ionization mass spectrometry (CIMS) is a powerful method for sensitive, selective, and fast quantification of a variety of atmospheric trace gases (Huey, 2007). NO_3_ is readily detected after reaction with iodide reagent ion as the nitrate anion at *m/z* 62; at this mass, however, there are several known interferences, including dissociative generation from N_2_O_5_, HNO_3_, and HO_2_NO_2_ (Slusher et al., 2004; Abida et al., 2011; Wang et al., 2014). There has been more success with the quantification of N_2_O_5_, usually as the iodide cluster ion at *m/z* 235 (Kercher et al., 2009), though accurate N_2_O_5_ measurement at *m/z* 62 has been reported from recent aircraft measurements with a large N_2_O_5_ signal (Le Breton et al., 2014).

Two groups have used laser-induced fluorescence (LIF) to quantify NO_3_ (and N_2_O_5_ through thermal dissociation) in ambient air (Wood et al., 2003; Matsumoto et al., 2005a, b). The major drawback of this method is the relatively low fluorescence quantum yield of NO_3_, and hence the method has not gained wide use.

Another technique that was demonstrated to be capable of measuring NO_3_ radicals at atmospheric concentration is matrix isolation electron spin resonance (MIESR) (Geyer et al., 1999). Although the technique allows simultaneous detection of other radicals (including HO_2_ and NO_2_), it has not been used extensively, probably because of its complexity.

Recently, a variety of in situ NO_3_ (Dorn et al., 2013) and N_2_O_5_ (Fuchs et al., 2012) measurement techniques were compared at the SAPHIR chamber in Jülich, Germany. All instruments measuring NO_3_ were optically based (absorption or fluorescence). N_2_O_5_ was detected as NO_3_ after thermal decomposition in a heated inlet by either CRDS or LIF. Generally, agreement within the accuracy of instruments was found for all techniques detecting NO_3_ and/or N_2_O_5_ in this comparison exercise. This study showed excellent agreement between the instruments on the single-digit ppt NO_3_ and N_2_O_5_ levels with no noticeable interference due to NO_2_ and water vapor for instruments based on cavity ring-down or cavity-enhanced spectroscopy. Because of the low sensitivity of LIF instruments, N_2_O_5_ measurements by these instruments were significantly noisier compared to the measurements by cavity-enhanced methods. The agreement between instruments was less good in experiments with high aerosol mass loadings, specifically for N_2_O_5_, presumably due to enhanced, unaccounted loss of NO_3_ and N_2_O_5_ demonstrating the need for regular filter changes in closed-cavity instruments. Whereas differences between N_2_O_5_ measurements were less than 20 % in the absence of aerosol, measurements differed up to a factor of 2.5 for the highest aerosol surface concentrations of 5 × 10^8^ nm^2^ cm^−3^. Also, differences between NO_3_ measurements showed an increasing trend (up to 50 %) with increasing aerosol surface concentration for some instruments.

#### 2.4.2 Gas-phase organic nitrate measurements

Analytical techniques to detect gaseous organic nitrates have been documented in a recent review by Perring et al. (2013). Sample collection techniques for organic nitrates include preconcentration on solid adsorbents (Atlas and Schauffler, 1991; Schneider and Ballschmiter, 1999; Grossenbacher et al., 2001), cryogenic trapping (Flocke et al., 1991) or collection in stainless steel canisters (Flocke et al., 1998; Blake et al., 1999), or direct sampling (Day et al., 2002; Beaver et al., 2012).

The approaches to the analysis of the organic nitrates fall into three broad categories. First, one or more chemically speciated organic nitrates are measured by a variety of techniques including liquid chromatography (LC) (Kastler et al., 2000) or gas chromatography (GC) with electron capture detection (Fischer et al., 2000), GC with electron impact or negative-ion chemical ionization mass spectrometry (GC-MS) (Atlas, 1988; Luxenhofer et al., 1996; Blake et al., 1999, 2003a, b; Worton et al., 2008), GC followed by conversion to NO and chemiluminescent detection (Flocke et al., 1991, 1998), GC followed by photoionization mass spectrometry (Takagi et al., 1981), GC followed by conversion of organic nitrates to NO_2_ and luminol chemiluminescent detection (Hao et al., 1994), CIMS (Beaver et al., 2012; Paulot et al., 2012), and proton transfer reaction MS (PTR-MS) (Perring et al., 2009). Second, the sum of all organic nitrates can be measured directly by thermal dissociation to NO_2_, which is subsequently measured by LIF (TD-LIF) (Day et al., 2002), CRDS (TD-CRDS) (Paul et al., 2009; Thieser et al., 2016), or cavity-attenuated phase shift spectroscopy (TD-CAPS) (Sadanaga et al., 2016). Finally, the sum of all organic nitrates can be measured indirectly as the difference between all reactive NO*_x_* except for organic nitrates and total oxidized nitrogen (NO*_y_* ) (Parrish et al., 1993).

Recent advances in adduct ionization utilize detection of the charged cluster of the parent reagent ion with the compound of interest. This scheme is then coupled to high-resolution time-of-flight (HR-ToF) mass spectrometry. The combination of these methods allows the identification of molecular composition due to the soft ionization approach that minimizes fragmentation. Multifunctional organic nitrates resulting from the oxidation of BVOC have been detected using CF_3_O^−^ (Bates et al., 2014; Nguyen et al., 2015; Schwantes et al., 2015; Teng et al., 2015) and iodide as reagent ions (Lee et al., 2014a, 2016; Xiong et al., 2015, 2016; Nah et al., 2016b).

#### 2.4.3 Online analysis of particulate matter

Total (organic plus inorganic) mass of particulate nitrates is routinely quantified using online AMS (Jayne et al., 2000; Allan et al., 2004), from which the mass of organic nitrates can be obtained by three techniques. First, the ${\text{NO}}^{+}/{\text{NO}}_{2}^{+}$ ratio (or ${\text{NO}}_{2}^{+}/{\text{NO}}^{+}$ ratio) in the mass spectra is used to distinguish organic from inorganic nitrates (Fry et al., 2009, 2013; Farmer et al., 2010; Xu et al., 2015b; Kiendler-Scharr et al., 2016). It is noted that the ${\text{NO}}_{2}^{+}/{\text{NO}}^{+}$ approaches zero in the case of low or nonexistent ${\text{NO}}_{2}^{+}$ signal, while ${\text{NO}}^{+}/{\text{NO}}_{2}^{+}$ gives large numbers. Second, positive matrix factorization (PMF) of data matrices including the NO^+^ and ${\text{NO}}_{2}^{+}$ ions in addition to organic ions (Sun et al., 2012; Hao et al., 2014; Xu et al., 2015b) is used. Third, the particulate inorganic nitrate concentration, as measured by an independent method such as ion chromatography, is subtracted from the total particulate nitrate concentration (Schlag et al., 2016; Xu et al., 2015a, b). A detailed comparison of these three methods is presented in Xu et al. (2015b). As the ${\text{NO}}^{+}/{\text{NO}}_{2}^{+}$ ratio in AMS data is dependent on instruments and the types of nitrates (inorganic and organic nitrates from different VOC oxidations), different strategies were developed when using this method to estimate particulate organic nitrates (Fry et al., 2013; Xu et al., 2015b).

A specialized inlet that selectively scrubs gaseous organic nitrates or collects particulate mass on a filter has been coupled to some of the techniques summarized in this section and utilized to observe particulate organic nitrates in the ambient atmosphere and laboratory studies. A TD-LIF equipped with a gas-scrubbing denuder (Rollins et al., 2010, 2012) and the filter inlet for gases and aerosols (FIGAERO) (Lopez-Hilfiker et al., 2014) at the front end of an iodide adduct HR-ToF-CIMS are examples (Lee et al., 2016; Nah et al., 2016b).

#### 2.4.4 Offline analysis of particulate matter

Owing to its ability to analyze polar organic compounds without a prior derivatization step, liquid chromatography coupled to MS (HPLC/MS) is well suited for the characterization of SOA compounds originating from the reactions of BVOC and NO_3_. Unlike in GC/MS methods, a soft ionization technique such as electrospray ionization (ESI) is utilized to ionize target analytes in the LC/MS technique. In the ESI/MS, target analytes are detected as a cation adduct of a target analyte (e.g., [*M* + H]^+^ or [*M* + Na]^+^) for a positive mode or a deprotonated form of a target analyte ([*M* − H]^−^) for a negative mode. As a biogenic SOA compound typically bears a functional group, such as a carboxylic group or a sulfate group, that easily loses a proton, the negative-mode ESI ((−)ESI) is commonly applied to detect SOA compounds. High-resolution MS such as TOF or Fourier transform ion cyclotron (FTICR) MS is commonly used to assign chemical formulas for SOA compounds unambiguously.

The LC/(−)ESI-MS technique played a crucial role in relating the formation of organosulfates (OS) and nitrooxy-organosulfates (NOS) to NO_3_-initiated oxidation of BVOC in laboratory-generated and ambient SOA. Since these earlier works, a number of studies have reported the presence of OS and/or NOS compounds in ambient samples (Table S4), though most studies do not connect these compounds explicitly to the NO_3_ oxidation of BVOC. It should be noted that the direct infusion (−)ESI-MS technique rather than LC/(−)ESI-MS is often used for the analysis of fog, rainwater, and cloud water samples as diluted liquid water samples can be injected into the ion source directly without a sample pretreatment procedure. However, caution is warranted for the direct infusion technique because it cannot separate isobaric isomers and it is susceptible to ion suppression, especially from the presence of inorganic ions in the samples.

Whilst the LC or direct infusion (−)ESI-MS techniques have been successfully applied for the detection of the oxidation products from NO_3_-BVOC reactions, the techniques have been less successful in quantifying these compounds, mainly due to the lack of authentic standard compounds. The synthesis of these compounds should be a priority for future studies.

Finally, total organic nitrate functional groups within the particle phase have been quantified in ambient air using FTIR of particles collected on ZnSe impaction disks (low-pressure cascade impactor, size segregated) or Teflon filters (PM_1_) (Mylonas et al., 1991; Garnes and Allen, 2002; Day et al., 2010). The organic nitrate content of particles can be quantified offline as well by collection on quartz fiber filters, extraction into solution (e.g., with water–acetonitrile mixtures), and analysis using standard wet chemistry techniques such as high-pressure liquid chromatography coupled to electrospray ionization mass spectrometry (HPLC-ESI-MS) (Angove et al., 2006; Perraud et al., 2010; Draper et al., 2015).

### 2.5 Field observations

This section surveys the current literature on field observations of nitrate radicals and BVOC (Sect. 2.5.1), and organic nitrate aerosol attributable to NO_3_-BVOC chemistry (Sect. 2.5.2).

#### 2.5.1 Nitrate radicals and BVOC

A few years after the first measurement of tropospheric NO_3_ (Noxon et al., 1980; Platt et al., 1980), it was recognized that the nitrate radical is a significant sink for BVOC, especially monoterpenes in terrestrial ecosystems and dimethyl sulfide (DMS) in maritime air influenced by continental NO*_x_* sources (Winer et al., 1984). The conclusion was based upon computer simulations using NO_3_ concentrations measured in field studies in the western US and Europe, and measured rate constants of NO_3_ with olefins. The scenarios in these simulations showed very low monoterpene concentrations in the early morning that were directly attributable to BVOC reactions with NO_3_. An analysis of NO_3_ formation rates at several urban and rural sites in Scandinavia (Ljungström and Hallquist, 1996) resulted in the conclusion that while night-time urban loss of NO_3_ is dominated by reaction with NO, the loss in rural regions is likely dominated by reactive hydrocarbons, especially monoterpenes.

Due to the fast reactions of NO_3_ with BVOC, lifetimes of NO_3_ in biogenically influenced environments can be very short, making simultaneous detection of VOC and NO_3_ in biogenic regions very difficult. For this reason, several studies have inferred levels of NO_3_ and its role in processing BVOC using observational analysis and supporting modeling. In particular, the rapid decay of isoprene after sunset has received considerable attention. Measurements of BVOC ~ 1–2 m above canopy level in a loblolly pine plantation in Alabama during the 1990 ROSE program (Goldan et al., 1995) were used to infer a nighttime NO_3_ mixing ratio of only 0.2 ppt and NO_3_ lifetime of only 7 s due to high levels of monoterpenes. The 4 h decay time of isoprene after sun-set could not be accounted for by gas reactions with NO_3_ and O_3_ although the decrease in the *α*- */ β*-pinene ratio at night was consistent with known NO_3_ and O_3_ chemistry. As part of the North American Research Strategy for Tropospheric Ozone – Canada East (NARSTO-CE) campaign, measurements of BVOC were made in Nova Scotia in a heavily forested region (Biesenthal et al., 1998). A box-model simulation based on the observational analysis found that the short lifetime of isoprene at night (*τ* = 1–3 h) could not be explained by the NO_3_ radical, which was estimated to be 0.1 ppt maximum at night due to low NO*_x_* and O_3_ levels and high monoterpene emissions. When OH yields from ozonolysis of BVOC were included in the model, this nighttime OH oxidant could partially account for the isoprene decay. During the Southern Oxidants Study (SOS) campaign in Nashville, TN (Starn et al., 1998), a chemical box model was used to show that rapid nighttime decays of isoprene were consistent with simulated NO_3_ but only when the site was impacted by urban NO*_x_* emissions. During the PROPHET study, measurements of VOC were made in a mixed forest approximately 10 m above the canopy surface (Hurst et al., 2001). Isoprene decays at night had an average lifetime of ~ 2.7 h. Box modeling showed that O_3_ reactions as well as dry deposition were insufficient to account for the decay, and that the NO_3_ radical was a significant sink only after the majority of isoprene had already decayed. On some nights, oxidation by OH could account for all the decay but the decay rates were overpredicted. The authors concluded that vertical transport of isoprene-depleted air aloft contributes to the fast initial decay of isoprene, followed by nighttime OH, NO_3_, and O_3_ chemistry decay. Steinbacher et al. (2005) reported on surface measurements in the Po Valley at a site 200–300 m from the closest edge of a deciduous forest. Bimodal diurnal cycles of isoprene were observed with morning and evening maxima that were reproduced by a Eulerian model. Isoprene decay lifetimes of 1–3 h were partially explained by NO_3_ decay, although a dynamic influence on isoprene decrease seemed to be likely including horizontal and vertical dispersion. During the HOHenpeissenberg Photochemistry Experiment (HOHPEX) field campaign, BVOC were analyzed via 2-D GC at a site located on a hilltop above adjacent rural agricultural/forested area that is frequently in the residual layer at night (Bartenbach et al., 2007). For the reactive monoterpenes, a significant non-zero dependency of the concentration variability on lifetime was found, indicating that chemistry (as well as transport) was playing a role in determining the ambient VOC concentrations. The night-time analysis gave an estimate of the NO_3_ mixing ratio of 6.2 ± 4.2 ppt, indicating it was a significant chemical factor in depletion of monoterpenes.

While the studies above made indirect conclusions about the role of NO_3_ in BVOC processing, field studies including direct measurements of NO_3_ are key to confirming the above findings. Golz et al. (2001) reported measurements of NO_3_ by long-path DOAS at an eucalyptus forest site in Portugal during the FIELDVOC94 campaign in 1994. The DOAS beam passed directly over the canopy at heights of 15 and 25 m, and as a result, they were unable to measure NO_3_ above the 6 ppt instrumental detection limit despite NO_3_ production rates of 0.4 ppt s^−1^. Rapid reaction with BVOC limited the NO_3_ lifetime to approximately 20 s such that NO_3_ reactions dominated other indirect losses, such as heterogeneous N_2_O_5_ uptake. Simultaneous measurements of NO_3_ and VOC during the Berliner Ozonexperiment (BERLIOZ) campaign in 1998 allowed one of the first assessments of the NO_3_ budget in comparison to OH and O_3_ oxidants (Geyer et al., 2001b). Surface measurements at this semi-rural location close to forests found the NO_3_ radical above detection limit (2.4 ppt) on 15 of 19 nights with a maximum of 70 ppt, a steady-state lifetime ranging from 20 to 540 s and N_2_O_5_ ranging from 2 to 900 ppt. The two most significant losses of NO_3_ were found to be its direct reaction with olefins (monoterpenes dominating) and indirect loss due to heterogeneous hydrolysis of N_2_O_5_. Over the study, it was possible for the first time to quantify the relative contribution of the NO_3_ radical to oxidation of VOC as 28 (24 h) and 31 % for olefinic VOC (24 h) compared to the total oxidation via NO_3_, OH, and O_3_. As part of the 1999 SOS study, NO_3_, isoprene, and its oxidation products were measured at a suburban forested site in Nashville, TN (Stroud et al., 2002). The nitrate radical measured at multiple beam heights by DOAS had maximum mixing ratios of 100 ppt that were generally found to anticorrelate with isoprene levels with significant vertical gradients on some nights. Early evening losses of isoprene were attributable to reaction with the NO_3_ radical. During the Pacific 2001 Air Quality Study (PACIFIC 2001) field campaign, NO_3_ was measured by long-path DOAS at an elevated forested site in the lower Fraser Valley of British Columbia with beam-path nighttime NO_3_ levels up to a maximum of 50 ppt (average of nighttime boundary layer and residual layer) (McLaren et al., 2004). Simultaneous analysis of carbonyl compounds in aerosol samples (Liggio and Mclaren, 2003) during the study found that only monoterpene oxidation products pinonaldehyde and nopinone (not reported) were enhanced in aerosol filters collected at night, evidence of the role of NO_3_ in nighttime oxidation of BVOC in the valley. In 2004 measurements of NO_3_ and N_2_O_5_ by CRDS, isoprene and its oxidation products were made on board the NOAA P-3 aircraft as part of the New England Air Quality Study (NEAQS) and International Consortium for Atmospheric Research on Transport and Transformation (ICARTT) campaigns in the northeast US (Brown et al., 2009). These studies found a very clear anticorrelation between isoprene levels after dark and NO_3_ mixing ratios, which varied as high as 350 ppt when isoprene was absent from the air mass. The loss frequencies (i.e., first-order loss rate constants) of NO_3_ were strongly correlated with the loss rate constant of NO_3_ with isoprene for lifetimes less than 20 min, clearly showing that isoprene was the most important factor determining the lifetime of NO_3_. It was also shown that more than 20 % of emitted isoprene was oxidized at night and that 1–17 % of SOA was contributed by NO_3_-isoprene oxidation. A number of recent studies have also investigated the role of NO_3_+ BVOC chemistry in more polluted areas. In many urban areas, the NO_3_+ BVOC chemistry occurs in parallel to heterogeneous NO_3_ / N_2_O_5_ chemistry and reactions of NO_3_ with anthropogenic VOC. Examples of such environments have been discussed in Brown et al. (2011, 2013) and Stutz et al. (2010) who presented observations in Houston, TX. Brown et al. (2011) and Stutz et al. (2010) found that up to 50 % of the NO_3_+ VOC reactions in Houston are driven by isoprene, with the other VOC emitted by industrial sources. Surprisingly, heterogeneous NO_3_ / N_2_O_5_ chemistry plays a minor role in Houston. Brown et al. (2011) also point out that the nocturnal VOC oxidation by NO_3_ dominates over that by ozone. Nocturnal NO_3_ formation rates were rapid and comparable to those of OH during the day. Crowley et al. (2011) compared NO_3_ chemistry in air masses of marine, continental, and urban origin at a field site in southern Spain. Under all conditions, NO_3_+ BVOC reactions (predominately *α*-pinene and limonene) contributed to the overall NO_3_ reactivity, confirming other observations that concluded that this chemistry is important in all environments where BVOC sources are present. In the southeastern US summer, this importance extends even through the daytime, when photolysis and NO reactions compete (Ayres et al., 2015). The NO_3_+ BVOC reaction rates observed in these studies imply a high production rate of SOA and organic peroxy radicals.

#### 2.5.2 Organic nitrate aerosols

There are many factors that motivate understanding organic nitrate in the particulate phase through field deployment of a variety of instrumentation, much of which is described in other sections of this review. Nitrogen-containing organic fragments (not necessarily organic nitrates) have been identified in atmospheric particles using mass spectrometric techniques (Reemtsma et al., 2006; Farmer et al., 2010; O’Brien et al., 2014). Total atmospheric organic nitrates, as well as organic nitrates segregated by phase, also have been measured in the atmosphere using techniques such as TD-LIF, CIMS, etc. (Day et al., 2003; Beaver et al., 2012). Given these observations and the propensity of organic nitrate compounds to partition to the condensed phase to create SOA (Rollins et al., 2013), it is critical to determine the level of organic nitrates that reside specifically in the atmospheric aerosol phase under typical ambient conditions and to identify the chemical and physical processes that determine their concentrations. It is also important to note that formation of SOA that contains organic nitrate groups has the potential to sequester NO*_x_*, thereby influencing the cycling of atmospheric oxidants.

**Figure 5:**
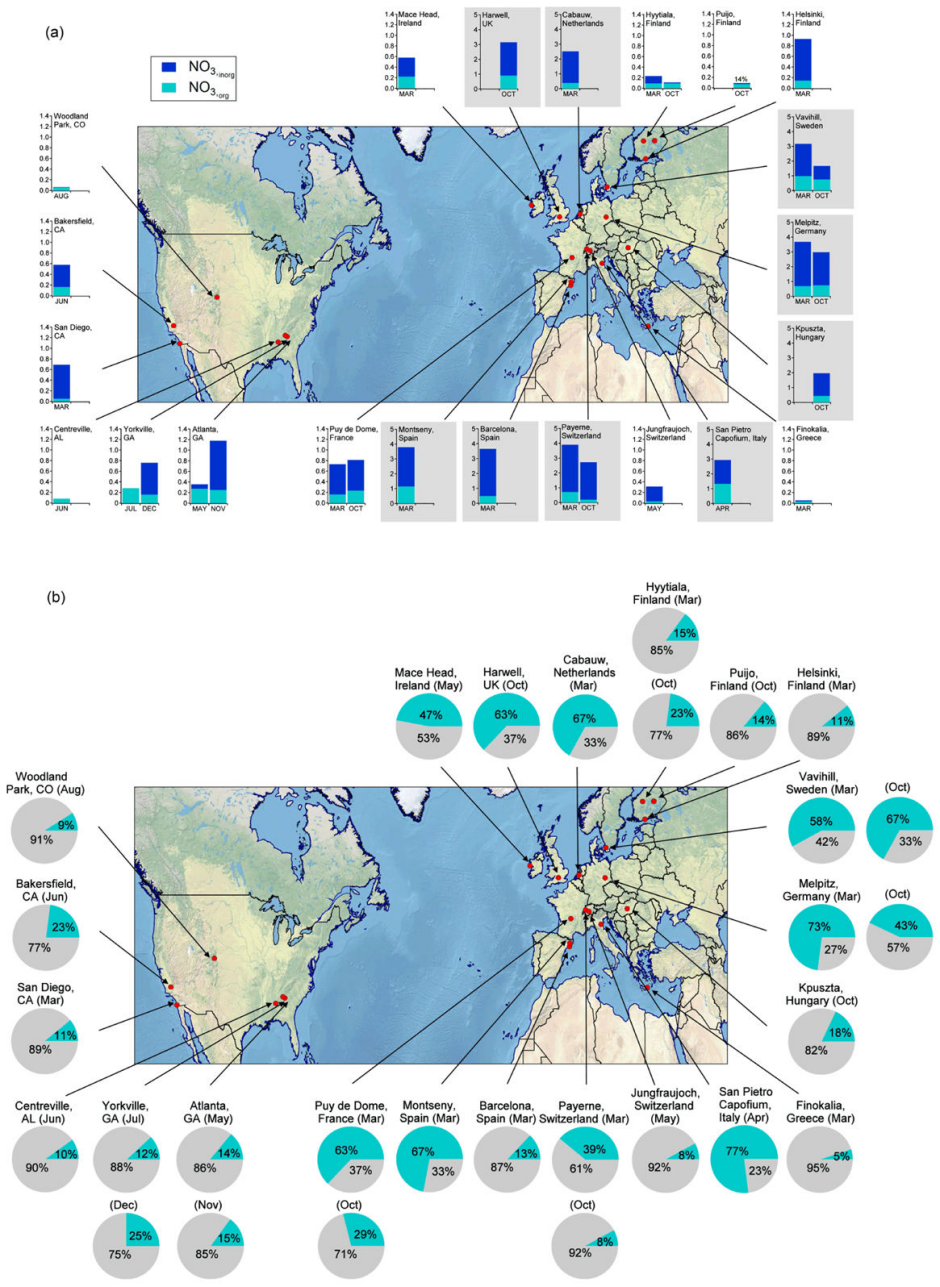
**(a)** Average mass concentrations (in μg m^−3^, ambient temperature and pressure) of submicrometer particulate organic nitrates (NO_3, org_) and particulate inorganic nitrates (NO_3, inorg_) in different months at multiple sites. The concentrations correspond to mass concentrations of –ONO_2_ functionality. Note that the *y* axis is different for sites with total nitrates greater than 1 μg m^−3^ (shaded). Detailed information and measurements for each site are provided in Table S5. **(b)** Percentage (by mass; cyan) of submicrometer particulate organic nitrate aerosols in ambient organic aerosols in different months at multiple sites. Detailed information and measurements for each site are provided in Table S5.

Organic nitrates in urban PM that were identified using functional group analyses such as FTIR spectroscopy have been attributed to emission of nitrogen-containing primary organic aerosol or to involvement of reactive nitrogen compounds in SOA formation chemistry (Mylonas et al., 1991; Garnes and Allen, 2002; Day et al., 2010). Other more advanced techniques, such as TD-LIF enhanced with the ability to separate phases or techniques to obtain high-resolution mass spectra (HR-ToF-AMS), have been utilized to quantify the amount of organic nitrate in particles in areas less likely to be influenced strongly by BVOC emissions, such as urban areas or areas influenced by oil and gas operations (Lee et al., 2015). Of specific interest here, however, are observations of organic nitrate PM in areas with a significant influence of BVOC, especially if co-located measurements allow for insight into the role that NO_3_ plays in the initial BVOC oxidation step. As such, we focus here on online measurements and on measurements that allow specific attribution to BVOC-NO_3_ reactions. Such measurements broadly can be categorized by region of sampling: the eastern United States (US), the western US, and Europe. Figure 5a summarizes average mass concentrations of submicrometer particulate organic nitrates (NO_3, org_) and particulate inorganic nitrates (NO_3, inorg_) in different months at multiple sites around the world. Figure 5b summarizes the corresponding percentage (by mass) of submicrometer particulate organic nitrate aerosols in ambient organic aerosols. Detailed information and measurements for each site are provided in Table S5.

##### Eastern United States

The first reports of aerosol organic nitrates in the southeastern (SE) US resulted from composition analysis of four daily PM filter samples from four Southeastern Aerosol Research and Characterization (SEARCH) network sites during summer 2004. Filters were analyzed for polar compounds, with particular focus on organosulfates, using offline chromatographic–MS methods (Gao et al., 2006; Surratt et al., 2007, 2008). Several nitrooxy organosulfates were identified, but the only one quantified (1–2 % of organic mass) was associated with *α*-pinene photooxidation or reaction with NO_3_. Several of the nitrooxy organosulfates were likely the same as products from BVOC-oxidant–NO*_x_* -seed systems based on comparison to spectra collected from chamber studies.

Brown et al. (2013) examined several nighttime aircraft vertical profiles in Houston (October 2006 during the Texas Air Quality Study 2006) that showed increases of total nitrate aerosol (and increases in AMS *m/z* 30 to *m/z* 46 ratio, the unit mass resolution approximation for ${\text{NO}}^{+}/{\text{NO}}_{2}^{+}$, indicative of organic nitrates; Farmer et al., 2010) and oxygenated organic aerosol (OOA). The OA versus carbon monoxide (CO) slopes at lower altitudes were consistent with SOA sources from NO_3_-BVOC reactions, with a combination of observations and zero-dimensional modeling showing 1 to 2 μg m^−3^ SOA formation from NO_3_-BVOC oxidation overnight with formation rates of 0.05 to 1 μg m^−3^ h^−1^.

More recently, during the summer Southern Oxidant and Aerosol Study (SOAS; mixed, semi-polluted forest) in Alabama (2013), an unprecedented suite of instruments quantified particle-phase organic nitrates using five different online methods: HR-ToF-AMS ( ${\text{NO}}^{+}/{\text{NO}}_{2}^{+}$), HR-ToF-AMS – PiLS (particle-into-liquid sampler) ion chromatography (PiLS-IC), HR-ToF-AMS (PMF), TD-LIF (denuded), and iodide CIMS. Total particle-phase nitrates increased throughout the night and peaked in early/mid-morning. Xu et al. (2015b) systematically evaluated the three AMS-related methods in estimating ambient particulate organic nitrate concentrations. Analysis presented in Xu et al. (2015a, b) using the HR-ToF-AMS – PiLS-IC method showed that organic nitrate functional groups comprised ~ 5–12 % of OA mass and correlated with PMF-derived less-oxidized oxygenated OA (LO-OOA). Two-thirds of the LO-OOA was estimated to be formed via NO_3_-BVOC chemistry (dominantly monoterpenes, ~ 80 %), with the balance due to ozone (O_3_)-BVOC chemistry. Organic nitrates were calculated to comprise 20–30 % of the LO-OOA factor. Ayres et al. (2015) used a measurement-constrained model for nighttime that compared NO_3_ production/loss to total organic nitrate (HR-ToF-AMS ${\text{NO}}^{+}/{\text{NO}}_{2}^{+}$, TD-LIF) formation to calculate a molar yield of aerosol-phase organic nitrates of 23–44 % (organic nitrate formed per NO_3_-BVOC reaction) that was dominated by monoterpene oxidation. They noted that the estimated yield was low compared to aggregated aerosol-phase organic nitrate yields, possibly due to rapid nitrate losses not considered in the model. Organic nitrate hydrolysis in the particle phase is one potential loss pathway, although recent laboratory studies suggest this process is slow for NO_3_ + *β*-pinene SOA (Boyd et al., 2015). Also, particle-phase organic nitrates were observed to contribute 30–45 % to the total NO*_y_* budget. Lee et al. (2016) quantified speciated particle-phase organic nitrates using iodide CIMS (88 individual C_4_-C_17_ mono/dinitrates). A large fraction was highly functionalized, with six to eight oxygen atoms per molecule. Diurnal cycles of isoprene-derived organic nitrates generally peaked during daytime, and monoterpene-derived organic nitrates peaked at night or during early/mid-morning. Using an observationally constrained diurnal zero-dimensional model, they showed that the observations were consistent with fast gas–particle equilibrium and a short particle-phase lifetime (2–4 h), again possibly due to hydrolysis if the field-derived lifetimes for particle-phase organic nitrates can be reconciled with recent laboratory studies (Boyd et al., 2015). The sum of the CIMS particle-phase organic nitrates (mass of nitrate functional groups only) was correlated with the two total aerosol organic nitrate AMS-based methods (*R*^2^ = 0.52, 0.67) with slopes of 0.63 and 0.90 (Lee et al., 2016). The CIMS sum was also correlated with the total measured with the TD-LIF method (*R*^2^ = 0.55); however, since the TD-LIF measurements were ~ 2–4 times higher (depending on period) than the AMS-based methods, the CIMS versus TD-LIF slope was substantially lower (0.19). Reasons for the differences between the total organic nitrate measured by different methods have been investigated but remain unclear.

A seasonal and regional survey of particle-phase organic nitrates is reported by Xu et al. (2015b) using a HR-ToF-AMS and an aerosol chemical speciation monitor (ACSM) (Ng et al., 2011) at four rural and urban sites in the greater Atlanta area (2012–2013) and in Centreville, AL (summer 2013 only, SOAS). They show strong diurnal cycles during summer, peaking early/mid-morning, and cycles with similar timing but smaller magnitude during winter. The concentrations were slightly higher in summer, which was attributed to compensating effects of source strength and gas–particle partitioning. Shallower boundary layers during winter also may have played a role in making the summer and winter concentrations more similar (Kim et al., 2015).

Fisher et al. (2016) report a broad regional survey of particle-phase (and gas-phase) organic nitrates (HR-ToF-AMS ${\text{NO}}^{+}/{\text{NO}}_{2}^{+}$) during summertime for the Studies of Emissions and Atmospheric Composition, Clouds and Climate Coupling by Regional Surveys (SEAC4RS) aircraft campaign (August–September, 2013, SE US only) as well as the ground-based SOAS measurements. A substantial vertical gradient was observed in particle-phase organic nitrates, with concentrations decreasing by several-fold from the boundary/residual layer into the free troposphere. Consistent with SOAS ground observations, 10–20 % of observed boundary layer total (gas plus particle) organic nitrates were in the particle phase for the aircraft measurements.

In addition to the measurements made in the SE US, characterization of aerosol organic nitrates has been performed in New England. As part of the New England Air Quality Study (NEAQS) in summer 2002, Zaveri et al. (2010) observed evolution of aerosols in the nocturnal residual layer with an airborne quadrupole (Q)-AMS in the Salem Harbor power plant plume. The aerosols were acidic and internally mixed, suggesting that the observed nitrate was in the form of organic nitrate and that the enhanced particulate organics in the plume were possibly formed from NO_3_-initiated oxidation of isoprene present in the residual layer.

##### Western United States

Significant work on understanding ambient organic nitrate formation from BVOC-NO_3_ has been performed in California. During the California Research at the Nexus of Air Quality and Climate Change (CalNex) field campaign from mid-May through June 2010, Rollins et al. (2012, 2013) measured particulate total alkyl and multifunctional nitrates (*p*ΣANs) with TD-LIF at a ground site in Bakersfield, California. They attributed the increase in *p*ΣAN concentrations at night to oxidation of BVOC by NO_3_ forming SOA, with an estimated 27 to 40 % of the OA growth due to molecules with nitrate functionalities. On average, 21 % of ΣANs were in the particle phase and increased with OA, which was fit to a volatility basis set in which *p*ΣANs / ΣANs increased from ~ 10 % at < 1 μg m^−3^ and plateaued at ~ 30 % by ~ 5 μg m^−3^. At the same site, using PMF analysis of FTIR and HR-ToF-AMS measurements, Liu et al. (2012a) showed that the organic nitrate-containing biogenic SOA condensed onto 400 to 700 nm sized primary particles at night. As part of the Carbonaceous Aerosol and Radiative Effects Study (CARES) in June 2010, Setyan et al. (2012) observed enhanced SOA formation due to interactions between anthropogenic and biogenic emissions at a forest site in the foothills of the Sierra Nevada mountains, approximately 40 km downwind of Sacramento. While nitrate accounted for only ~ 4 % of the particle mass measured by a HR-ToF-AMS, it was attributed potentially to organic nitrates based on the much higher ${\text{NO}}^{+}/{\text{NO}}_{2}^{+}$ ion ratio than observed in pure ammonium nitrate.

During the Rocky Mountain Biogenic Aerosol Study field campaign in Colorado’s Front Range (rural coniferous montane forest) (BEACHON-RoMBAS) from July to August 2011, Fry et al. (2013) observed aerosol-phase organic nitrates by optical spectroscopic (denuded TD-LIF) and mass spectrometric (HR-ToF-AMS, ${\text{NO}}^{+}/{\text{NO}}_{2}^{+}$) instruments. The two methods agreed well on average (AMS/TD-LIF slope of 0.94–1.16, depending on averaging method) with a fair correlation (*R*^2^ = 0.53). Similar to studies in other forested environments, the organic nitrate concentration was found to peak at night. The organic nitrate concentration was positively correlated with the product of the nitrogen dioxide and O_3_ mixing ratios but not with that of O_3_ alone; this suggested nighttime NO_3_-initiated oxidation of monoterpenes as a significant source of nighttime aerosol organic nitrates. The gas–particle partitioning also showed a strong diurnal cycle, with the fraction in the particle phase peaking at ~ 30 % at night and decreasing to a broad minimum of ~ 5 % during daytime, which suggests a change in composition in addition to thermodynamic partitioning effects.

##### Europe

Iinuma et al. (2007) analyzed ambient aerosol samples collected on filters in a Norway spruce forest in northeastern Germany during the BEWA campaign (Regional biogenic emissions of reactive volatile organic compounds from forests: process studies, modeling, and validation experiments) and compared the results to those from chamber studies. The filter extracts were analyzed using LC-ESI-ToF-MS in parallel to ion trap MS. Several nitrooxy organosulfates with significant mass in the BEWA ambient samples were enhanced in the nighttime samples relative to the daytime samples. Their abundance in the nighttime samples strongly suggests that NO_3_-monoterpene chemistry in the presence of sulfate aerosols has an important role in the formation of these nitrooxy organosulfate aerosols.

A similar study by Gómez-González et al. (2008) focused on isoprene through LC-multidimensional MS (MS*^n^*) analysis of filter samples from both chamber studies and ambient summer day/night PM_2.5_ samples from K-Puszta, Hungary, a mixed deciduous/coniferous forest site. Although not the focus of the study, they confirmed the presence of significant quantities of nitrooxy organosulfates that were enhanced in the nighttime samples over the daytime samples.

Initial online evidence of the production of organic nitrate aerosols in Europe was provided by Allan et al. (2006) when studying nucleation events driven by BVOC oxidation in Hyytiälä, a (boreal) forested region in Finland. The Q-AMS *m/z* 30 to *m/z* 46 ratio (the unit mass resolution approximation for ${\text{NO}}^{+}/{\text{NO}}_{2}^{+}$ ratio) was frequently found to be very high, ~ 10, for a distinct organic Aitken mode that became apparent late in the afternoon and increased at night. They hypothesized that the excess *m/z* 30 (NO^+^) signal was associated with organic nitrates, although could not rule out amine contributions. During the same field study, Vaattovaara et al. (2009) applied two tandem differential mobility analyzer methods to study the evolution of the nucleation-and Aitken-mode particle compositions at this boreal forest site. The results showed a clear anthropogenic influence on the nucleation- and Aitken-mode-particle compositions during the events and suggested organic nitrate and organosulfate aerosol was generated from monoterpene oxidation. Also, it was shown that organic nitrate was enhanced in aerosol exposed to elevated temperatures, implying low volatility of organic nitrates (Häkkinen et al., 2012).

More recently, Hao et al. (2014) used a HR-ToF-AMS on a tower in Kuopio, Finland, 224 m above a lake surrounded by a mixed forest of mostly coniferous (pine and spruce) mixed with deciduous trees (mostly birch) to measure submicron aerosol composition. The site also was influenced by urban emissions. A particular focus of the study was to separate organic and inorganic nitrate using PMF. They found that ~ 37 % of the nitrate mass at this location and time could be allocated to organic nitrate factors, the rest being inorganic nitrate. The organic nitrate aerosol was segregated into two organic factors, less-oxidized OOA (LO-OOA), and more-oxidized OOA (MO-OOA) (previously called SV- and LV-OOA, respectively); the majority (74 %) of the organic nitrate was found to be in the more volatile LO-OOA factor. Based on meteorology, the air mass source of the organic nitrate aerosol was from a sector with residential and forested areas. Again, the organic nitrate aerosol showed a diurnal trend that was highest at night.

An analysis of AMS data taken across Europe within EU-CAARI and EMEP intensive measurement campaigns (Kulmala et al., 2011; Crippa et al., 2014) has recently shown high organic nitrate contributions to total measured PM_1_ nitrate (Kiendler-Scharr et al., 2016). The spatial distribution and diurnal pattern of particulate organic nitrate indicate a gradient of concentration. High concentrations are found in source regions with NO*_x_* emissions and during the night. Low concentrations are found in remote regions and during the day. EURAD-IM simulations for Europe show an increase of SOA by 50 to 70 % when considering SOA formation by NO_3_ oxidation with maximum ground-level concentrations of SOA from NO_3_ oxidation in the range of 2 to 4 μg m^−3^ (Li et al., 2013; Kiendler-Scharr et al., 2016).

##### Summary of organic nitrate aerosol observations

Taken together, the observations of particle-phase organic nitrates in the US and Europe suggest that particle-phase organic nitrates (formed substantially via NO_3_-BVOC chemistry) are ubiquitous, especially in, but not limited to, summer. Their formation appears to play an important role in SOA formation, which can potentially be underestimated due to short particle-phase lifetimes. Regions with widespread NO*_x_* and BVOC emissions and a humid climate may create optimal conditions for a rapid life cycle of particle-phase organic nitrates.

### 2.6 Models of NO_3_-BVOC chemistry

To understand the implications of NO_3_-BVOC chemistry on atmospheric chemistry as a whole, under both current and future scenarios, the physical and chemical processes, such as those reported in Sect. 2.1 through Sect. 2.3, must be parameterized in numerical models. In this section, we summarize how these reactions are represented in current air quality models (AQMs).

#### 2.6.1 Chemical mechanisms

Organic nitrates are produced from the reactions of VOC with OH followed by NO as well as with NO_3_, and both of these pathways are represented in chemical mechanisms albeit at varying levels of detail. The use of the term “model” below refers to the treatment of BVOC + NO_3_ chemistry in lumped chemical mechanisms. The products formed from the OH-initiated (typically daytime) versus NO_3_-initiated (typically nighttime) chemistry may or may not be treated separately.

The NO_3_-BVOC reactions result in an RO_2_ that reacts with NO_3_, other RO_2_, HO_2_, or NO. RO_2_-NO reactions for NO_3_-initiated chemistry are relatively unimportant due to rapid reaction of NO with NO_3_ at night (Perring et al., 2009), but they are included in models. Unimolecular rearrangements of the NO_3_-initiated RO_2_ radical are not currently considered in models (Crounse et al., 2011). The products of the initial NO_3_-BVOC reaction may retain the nitrate group, thus forming an organic nitrate or releasing nitrogen as NO_2_. The branching between organic nitrate formation and N recycling is parameterized in models. Table 4 summarizes the gas-phase organic nitrate yields for isoprene and monoterpene oxidation by NO_3_ in a number of currently available chemical mechanisms. The yields represent the first-generation yields since products may react to form further organic nitrates or release NO_2_. The organic nitrate yield values span from 0 (e.g., SAPRC07 isoprene) to 100 % (e.g., MCM isoprene). Although GEOS-Chem v10-01 does not consider gas-phase monoterpene chemistry, the model has recently been updated to consider a 10–50 % yield of organic nitrates from the monoterpene-NO_3_ reaction independent of the nitrate-RO_2_ fate but dependent on monoterpene identity (Fisher et al., 2016). Differences in the organic nitrate yield from NO_3_ oxidation result from a number of causes including treatment of RO_2_ fate, assumptions about decomposition versus retention, and prioritization of functional group identity.

Some models parameterize the yield of organic nitrates as a function of RO_2_ fate while others, such as the carbon bond-based (CB) mechanisms, treat all RO_2_ fates the same. The MCM v3.3.1 also considers the yield of isoprene organic nitrates to be independent of RO_2_ fate, but monoterpene organic nitrate yields are variable between 0 and 100 % depending on RO_2_ fate. Differences in organic nitrate formation, due to treating the organic nitrate yield as a function of RO_2_ fate, may vary with atmospheric conditions. Reactions with both HO_2_ and RO_2_ are significant at night (Xie et al., 2013; Pye et al., 2015). RO_2_-NO_3_ may be important in urban areas or locations where BVOC concentrations are not so high as to deplete NO_3_ (Rollins et al., 2012).

Mechanisms differ in their assumptions about whether or not the organic nitrates from NO_3_-initiated chemistry release NO_2_ or retain the nitrate group. An example of this difference in treatment of organic nitrates can been seen in the reactions of nitrated peroxy radicals with different radicals (NO, HO_2_, RO_2_) predicted by SAPRC07 and MCM. MCM predicts greater loss of the nitrate group, while SAPRC tends to retain it, leading to either < 5 % (MCM) or > 50 % (SAPRC) organic nitrate yields.

In order to predict accurately the fates of RO_2_ and yield of organic nitrates, models must also include information on RO_2_ reaction rate constants. Some mechanisms use the same set of RO_2_ rate constants for all hydrocarbons. However, the MCM (Jenkin et al., 1997; Saunders et al., 2003) indicates that the RO_2_-HO_2_ rate constant should vary with carbon number (*n*) and predict *k* = 2.91 × 10^−13^ exp(1300/T) [1 − exp(−0.245*n*)] molec^−1^ cm^3^ s^−1^. The MCM RO_2_-RO_2_ rate constant varies between 2 × 10^−12^ cm^3^ molec^−1^ s^−1^ (based on C_1_-C_3_ primary RO_2_ with adjacent O or Cl) and 6.7 × 10^−15^ cm^3^ molec^−1^ s^−1^ for tertiary alkyl RO_2_ (based on *t*-C_4_H_9_O_2_). RO_2_-NO_3_ and RO_2_-NO rate constants are estimated as 2.3 × 10^−13^ and 9.0 × 10^−12^ cm^3^ molec^−1^ s^−1^ at 298 K.

AQMs and chemistry–climate models typically cannot handle the complexity associated with tracking each individual VOC and all its possible reaction products. As a result, surrogate species are often used to represent classes of compounds (e.g., CB05, which uses the designation NTR to indicate organic nitrates). This mapping can cause yields of organic nitrates to be falsely low in a mechanism if other functional groups are prioritized over nitrate in the mapping of predicted products to mechanism species. Compared to the other mechanisms in Table 4, SAPRC07 monoterpenes tend to have very low organic nitrate yields as a result of prioritization of peroxide and non-nitrate functional groups. If nitrate groups were prioritized, SAPRC07 would more closely resemble the “other monoterpene” yields from SAPRC07tic. In addition, the diversity across mechanisms in the RO_2_-HO_2_ monoterpene organic nitrate yields would be reduced such that they would all indicate > 50 % organic nitrate yields and all but the CB mechanisms would predict a 100 % yield of organic nitrates from RO_2_-HO_2_. The RO_2_-HO_2_ pathway is relatively unstudied in laboratory conditions due to difficulties in maintaining sufficient concentrations of both NO_3_ and HO_2_ radicals (Boyd et al., 2015; Schwantes et al., 2015).

#### 2.6.2 Influence on organic aerosol

Nitrate radical oxidation can lead to significant amounts of SOA on global and regional scales. Due to a lack of information on the identity and volatility of later-generation BVOC + NO_3_ products, most models parameterize SOA formation separately from gas-phase chemistry using either the Odum two-product (Odum et al., 1996) fit, volatility basis set (VBS) (Donahue et al., 2006) fit, or fixed yield (Table 5). Based on the understanding of SOA pathways at the time, Hoyle et al. (2007) found that up to 21 % of the global average SOA burden may be due to NO_3_ oxidation, and Pye et al. (2010) predicted ~ 10 % of global SOA production was due to NO_3_. Regional contributions to SOA concentrations can be much higher (Hoyle et al., 2007; Pye et al., 2010). Nitrate radical reactions themselves are estimated to account for less than 3 % of isoprene oxidation and less than 2 % of sesquiterpene oxidation globally; however, they account for 26 % of bicyclic monoterpene oxidation (Pye et al., 2010). Representations of monoterpene-NO_3_ SOA are more widespread in chemistry–climate models than other BVOC-NO_3_ SOA parameterizations due to the relatively early recognition of its high yields (e.g., Griffin et al., 1999) and relative importance for SOA. Inclusion of SOA from isoprene-NO_3_ is more variable as reflected in Table 5.

SOA from BVOC-NO_3_ reactions traditionally has been parameterized on the initial hydrocarbon reaction assuming semivolatile products and an Odum two-product approach (e.g., Chung and Seinfeld, 2002). This treatment is often implemented in parallel to the gas-phase chemistry, meaning that later-generation products leading to SOA are not identified. Information is still emerging on the fate of organic nitrates, and that information is just beginning to be included in models. Hydrolysis of particle-phase organic nitrates is one such process more recently considered with impacts for both O_3_ and PM in models (Hildebrandt Ruiz and Yarwood, 2013; Browne et al., 2014; Pye et al., 2015; Fisher et al., 2016).

#### 2.6.3 Influence on reactive nitrogen and ozone

The influence of BVOC nighttime oxidation on the nitrogen budget remains unclear. Current modeling efforts have mainly focused on the nighttime oxidation of isoprene, which is dominated by isoprene-NO_3_ reaction. This pathway is initialized via addition of NO_3_ to one of the double bonds, as discussed in Sect. 2.1.2. Due to the additional stabilization from alkoxy radical and nitrate functional groups (Paulson and Seinfeld, 1992), the yield of first-generation organic nitrates is relatively high (62–78 %; Table 2); they may react with NO_3_ again to produce secondary dinitrates (Perring et al., 2009; Rollins et al., 2009, 2012). Assuming little NO*_x_* is recycled from these organic nitrates, most models suggest that nighttime oxidation of isoprene by NO_3_ contributes significantly to the budget of organic nitrates (von Kuhlmann et al., 2004; Horowitz et al., 2007; Mao et al., 2013; Xie et al., 2013). Two recent studies (Suarez-Bertoa et al., 2012; Müller et al., 2014), however, suggest fast photolysis of carbonyl nitrates with high efficiency of NO*_x_* recycling, which could lead to release of NO*_x_* in the next day. Further modeling is required to investigate the importance of nighttime isoprene oxidation on the nitrogen budget.

Very little modeling effort has been dedicated to the influence of nighttime terpene oxidation on the nitrogen budget, mainly due to the lack of laboratory data on oxidation products and their fate. In contrast to isoprene, terpene emissions are temperature sensitive but not light sensitive (Guenther et al., 1995), leading to a significant portion of terpene emissions being released at night. The high yield of organic nitrates and SOA from the terpene-NO_3_ reaction (Fry et al., 2009, 2011, 2014; Boyd et al., 2015) provides an important sink for NO*_x_* at night, likely larger than for isoprene-NO_3_ over the eastern US (Warneke et al., 2004). Recent laboratory experiments suggest that aerosol organic nitrates can be either a permanent or temporary NO*_x_* sink depending on their monoterpene precursors (and hence nature of the resulting RO_2_) as well as ambient RH (Boyd et al., 2015; Nah et al., 2016b). In order to understand the impact of terpenes on nighttime chemistry, a fully coupled model of terpene-NO*_x_* chemistry will be required, as monoterpenes can be the dominant loss process for NO_3_ and N_2_O_5_ at night (Ayres et al., 2015).

While a significant portion of nitrogen is emitted at night (Boersma et al., 2008), the impact of nighttime chemistry on the initiation of the following daytime chemistry has received little attention in regional and global models. Different treatments of NO_3_ chemistry can result in 20 % change in the following daytime O_3_ concentration, as shown by a 1-D model study (Wong and Stutz, 2010) and box model simulations (Millet et al., 2016). This impact can be further complicated by uncertainty in emissions of BVOC and model resolutions. For example, a recent study by Millet et al. (2016) shows that in a city downwind of an isoprene-rich forest, daytime O_3_ can be largely modulated by the chemical removal of isoprene throughout the night. Such local-scale events may only be captured by a very high-resolution model with detailed characterization of emission sources. It is important to assess this impact on a global scale using 3-D chemistry models, owing to the profound coupling of boundary layer dynamics and chemistry. Quantifying the impact of BVOC-NO_3_ chemistry on NO*_x_* fate is important given the long-standing problem in current global and regional AQMs of a large overestimate of O_3_ over the eastern US in summer (Fiore et al., 2009).

#### 2.6.4 Comparison of field data with air quality models

Recent field campaigns (SOAS, SEAC4RS, EUCAARI, EMEP) have allowed for the attribution of SOA to NO_3_ oxidation to provide model constraints not previously available. Pye et al. (2015) and Fisher et al. (2016) implemented updated BVOC + NO_3_ chemistry in CMAQ and GEOS-Chem, respectively, to interpret data in the SE US during the summer of 2013 (SOAS and SEAC4RS). Model predictions of gas-phase monoterpene nitrates (primarily NO_3_ derived) were higher than the sum of C_10_H_17_NO_4_ and C_10_H_17_NO_5_ (Nguyen et al., 2015) by a factor of 2–3 (Fisher et al., 2016) and 7 (Pye et al., 2015), consistent with a significant fraction of the monoterpene nitrates being highly functionalized (Lee et al., 2016). The studies identified particle-phase hydrolysis as an important modulator of particulate organic-nitrate concentrations and organic nitrate lifetime. The GEOS-Chem simulation reproduced the particle-phase organic nitrate diurnal cycles (SOAS), boundary layer concentrations, and gas–particle partitioning reasonably well; however, it underestimated concentrations in the free troposphere, possibly due to measurement limitations and/or the implementation of rapid uptake followed by hydrolysis of all gas-phase organic nitrates in the model, which may not be valid for non-tertiary organic nitrates (Fisher et al., 2016).

## 3 Perspectives and outlook

Section 3 outlines perspectives on the implications of NO_3_-BVOC atmospheric chemistry with respect to (1) aerosol optical and physical properties; (2) health effects; (3) trends in NO*_x_* emissions and organic aerosols and their implications for control strategies related to particulate matter; (4) critical needs for analytical methods; (5) critical needs for models; (6) field studies in the developing world and under-studied areas; and (7) critical issues to address in future field and laboratory measurements in light of current understanding of this chemistry and trends in emissions.

### 3.1 Aerosol optical and physical properties

The climatic effects of atmospheric aerosols depend on their various physical and chemical properties. Hygroscopicity, cloud condensation nuclei (CCN) activity, optical properties (namely light absorption and scattering), and ability to act as CCN and ice nuclei (IN) are the key aerosol properties that would determine their ability to affect climate. Additional properties such as aerosol number size distribution, chemical composition, mixing state, and morphology will determine whether the aerosols will be optically important or whether they would affect cloud properties. These aerosol properties depend on the sources, aging processes, and removal pathways that aerosols experience in the atmosphere (Boucher, 2013).

Absorption by aerosol may affect the cloud lifetime and altitude due to heating of the atmosphere (Mishra et al., 2014). They can also change the atmospheric lapse rate, which in turn can result in modification in aerosol microphysics in mixed-phase, ice, and convective clouds (Boucher, 2013). In addition to direct emissions of known absorbing particles (black carbon, mineral dust, biomass burning aerosols), SOA may also have absorption properties. The absorbing component of organic carbon (OC), namely “brown carbon” (BrC), is associated with OC found in both primary and secondary OC and has a spectral-dependent absorption that smoothly increases from short visible to UV wavelengths (Bond and Bergstrom, 2006). It has been suggested that BrC is a component of SOA that is composed of high molecular weight and multifunctional species such as humic-like substances, organic nitrates, and organosulfate species (Andreae and Gelencser, 2006; Bond and Bergstrom, 2006; Ramanathan et al., 2007b; Laskin et al., 2015; Moise et al., 2015). Many modeling studies often assume that BC and mineral dust are the only two significant types of light-absorbing aerosols on the global scale. Therefore, they treat SOA as a purely scattering component that leads to climate cooling (Stier et al., 2007; Bond et al., 2011; Ma et al., 2012). However, observations suggest that BrC is widespread mostly around and downwind urban centers (Jacobson, 1999). In such places, BrC may have significant contribution, and in some cases it may dominate the total aerosol absorption at specific (short) wavelengths (Ramanathan et al., 2007a; Bahadur et al., 2012; Chung et al., 2012; Feng et al., 2013).

Based on observations, Chung et al. (2012) recently suggested that the direct radiative forcing of carbonaceous aerosols is +0.65 (0.5 to about 0.8) Wm^−2^, comparable to that of methane, the second most important greenhouse gas. This study emphasizes the important role of BrC and calls for better measurements of the absorption properties of BrC, specifically at short wavelengths where the absorption is most significant. Many previous studies have concentrated on primary particulate matter, mostly from biomass burning. However, these studies often neglected contributions to absorption due to BrC in SOA. There is ample laboratory and field evidence for the formation of such absorbing material in SOA (Chung et al., 2012; Lack et al., 2012). This absorbing component is the least characterized component of the atmospheric absorbing aerosols and constitutes a major knowledge gap, calling for an urgent need to identify the optical properties of the organic (BrC) component in SOA, and the chemical pathways leading to its formation and losses (Laskin et al., 2015; Lin et al., 2015; Moise et al., 2015).

Recently, Washenfelder et al. (2015) measured aerosol optical extinction and absorption in rural Alabama during the SOAS campaign. While they found that the majority of BrC aerosol mass was associated with biomass burning, a smaller (but not negligible) contribution was attributed to biogenically derived SOA. This fraction reached a daily maximum at night and correlated with particle-phase organic nitrates and is associated with nighttime reactions between monoterpenes and the NO_3_ radical (Xu et al., 2015a). Based on the above, it is concluded that SOA produced from reactions of NO_3_ with BVOC can be a major source of SOA during the night that may affect daytime aerosol loading. This important fraction of NO_3_-derived SOA can contribute to the direct radiative effect of SOA through scattering and absorption of incoming solar radiation.

Nitration of aromatic compounds (oxidation via NO_2_, NO_3_, N_2_O_5_) has a potential to form chromophores that can absorb solar radiation. Theoretical and experimental studies have shown that nitration of PAHs leads to nitro PAHs and their derivatives such as nitrophenols (Jacobson, 1999; Harrison et al., 2005; Lu et al., 2011). The nitro substituents on the aromatic ring in compounds enhance and shift the absorption to longer wavelengths (> 350 nm). Field studies report that nitrogen-containing mono- and polyaromatic SOA constituents absorb light at short (near-UV and visible) wave-lengths. The reaction products between NO_3_ and BVOC have the potential to form effective chromophores. Multifunctional organic nitrates and organosulfate compounds formed during the nighttime suggest that the SOA produced from NO_3_ reactions leads to formation of BrC that can absorb solar radiation (Iinuma et al., 2007).

Only a few studies have investigated optical properties of SOA partially composed of organic nitrates (Moise et al., 2015). Most existing literature on optical properties of organic nitrates in SOA has been focused on oxidation of anthropogenic precursor compounds (Jacobson, 1999; Nakayama et al., 2010; Lu et al., 2011; Liu et al., 2012b), while a few partially contradictory studies have examined SOA formed from NO_3_ reaction with biogenic precursors (Song et al., 2013; Varma et al., 2013). The typically high mass absorption coefficient (MAC) that was observed for anthropogenic high-NO*_x_* SOA can be partially attributed to the presence of nitroaromatic groups, for example, via the nitration of PAHs (Jacobson, 1999; Lu et al., 2011). Song et al. (2013) examined optical properties of SOA formed by NO_3_+ O_3_+ *α*-pinene. With neutral seed aerosol, organic nitrates were present but observed to be non-absorbing; however, with acidic seed aerosol, SOA were strongly light absorbing, which the authors attributed to nitrooxy organosulfates formed via aldol condensation. Varma et al. (2013) measured absorption of NO_3_ +*β*-pinene SOA and found a higher refractive index than when oxidation was via OH or O_3_, and attributed to the difference to the low HC / NO*_x_* ratio and presence of organic nitrates in the particle phase.

**Figure 6:**
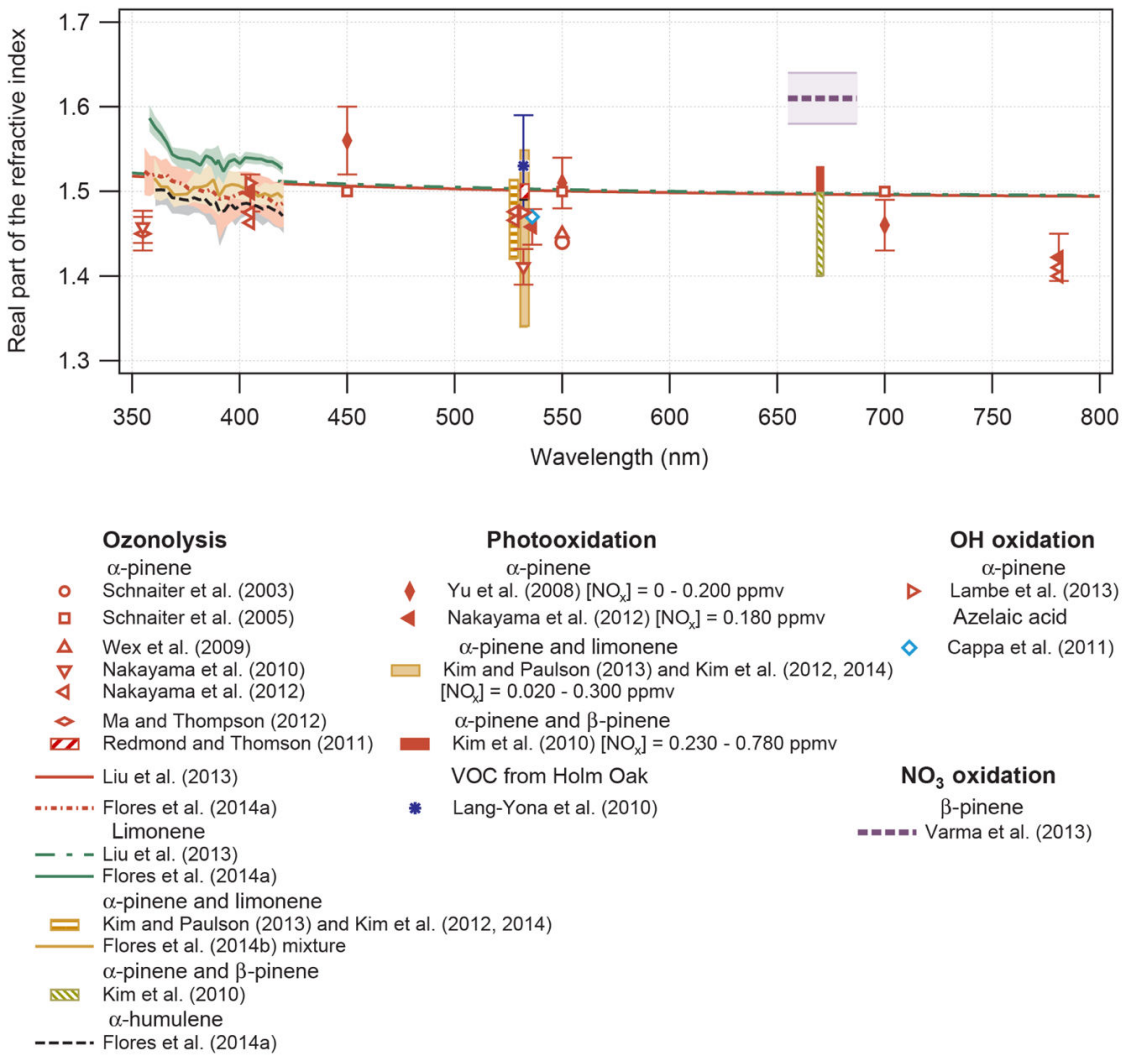
The real part of refractive index (RI) (mr) for biogenic SOA compiled from several chamber studies. The legend specifies the precursor type and oxidation pathway as well as the reference. The figure is reprinted with permission from Moise et al. (2015).

Laboratory and field studies suggest that SOA formed by nighttime chemistry can have profound regional and possible global climatic effects via their absorbing properties. However, the optical properties of NO_3_-containing SOA are not well known. Varma et al. (2013) measured a high value for the refractive index real part value of 1.61 (±0.03) at *λ* = 655– 687 nm following reactions of NO_3_ with *β*-pinene. This value is significantly higher than values observed following OH- and ozone-initiated terpene oxidation (Fig. 6) (Moise et al., 2015). This has been attributed to the high content (up to 45 %) of organic nitrates in the particle phase (Varma et al., 2013).

Key physical parameters of aerosols include particle size and number, volatility, viscosity, hygroscopicity, and CCN activity. While it is clear that atmospheric particle size increases through condensation of BVOC + NO_3_ oxidation products, the effect of NO_3_ oxidation on particle number is not usually studied in laboratory experiments. Very little is known about the volatility of SOA from NO_3_, with field studies from Hyytiälä indicating that organic nitrates may have low volatility (Häkkinen et al., 2012). Viscosity is not known. Few studies report the hygroscopicity and CCN activity of SOA from NO_3_ oxidation of BVOC. A study by Suda et al. (2014) showed that organic compounds with nitrate functionality (compared to other functional groups such as hydroxyl, carbonyl, hydroperoxide) have the lowest hygroscopicity and CCN efficiency. Recently, Cerully et al. (2015) reported that the hygroscopicity of less-oxidized OOA (LO-OOA, mostly from BVOC + NO_3_) is lower than other OA subtypes (MO-OOA and isoprene-OA) resolved by PMF analysis of AMS data from the SOAS campaign. As monoterpenes + NO_3_ reactions can contribute ~ 50 % of nighttime OA production (Xu et al., 2015a), results from Cerully et al. (2015) suggested that it is possible that SOA formed from NO_3_ oxidation of BVOC is less hygroscopic than OA formed from other oxidation pathways.

### 3.2 Health effects

Nitrated organic compounds also pose adverse health effects (Franze et al., 2003, 2005; Pöschl, 2005; Gruijthuijsen et al., 2006; Pöschl and Shiraiwa, 2015). In particular, several studies have reported that biological particles such as birch pollen protein can be nitrated by O_3_ and NO_2_ in polluted urban air (Franze et al., 2005; Reinmuth-Selzle et al., 2014). The mechanism of protein nitration involves the formation of long-lived reactive oxygen intermediates, which are most likely tyrosyl radicals (phenoxy radical derivatives of tyrosine) (Shiraiwa et al., 2011). The resulting organic nitrates were found to enhance the immune response and the allergenicity of proteins and biomedical data suggest strong links between protein nitration and various diseases (Gruijthuijsen et al., 2006). Inhalation and deposition of organic nitrates into lung lining fluid in the human respiratory tract may lead to hydrolysis of organic nitrates forming HNO_3_, which may reduce pulmonary functions (Koenig et al., 1989). Consequently, inhalation of aerosols partially composed of nitrated proteins or nitrating reagents might promote (i) immune reactions, (ii) the genesis of allergies, (iii) the intensity of allergic diseases, and (iv) airway inflammation. Toxicity of nitrated SOA compounds is still unclear. In the light of these observations and remaining uncertainties, the effect of organic nitrates present in biogenic SOA on human health should be a focus of future studies.

Formaldehyde is an important source of atmospheric radicals as well as a major hazardous air pollutant (HAP). It is a degradation product of almost every VOC in the atmosphere, and BVOC are known to contribute substantially to ambient concentrations of formaldehyde (Luecken et al., 2012). The overall yield of formaldehyde from BVOC-NO_3_ reactions is lower than from corresponding OH reactions, indicating that any changes in the relative distribution of oxidation routes will have a corresponding change in formaldehyde (and thus oxidant regeneration and HAP exposure).

### 3.3 Trends in NO*_x_* emissions and organic aerosols – implications for air quality control strategies

Nitrogen oxide emissions are converted to NO_3_ and thus affect nitrate-derived SOA. In the United States, where NO*_x_* emissions are dominated by fuel combustion, regulatory actions have resulted in decreasing NO*_x_* levels after increases from 1940 to 1970 (Nizich et al., 2000) and relatively stable levels between ~ 1970 and ~ 2000 (Richter et al., 2005). NO*_x_* emissions in the US are estimated to have decreased by roughly 30–40 % in the recent past (between 2005 and 2011/2012), as reflected in satellite-observed NO_2_, ground-based measurements, and the Environmental Protection Agency (EPA) National Emission Inventory (NEI) (Russell et al., 2012; Xing et al., 2013, 2015; Hidy et al., 2014; Tong et al., 2015). Recent decreases in NO*_x_* have been attributed to the mobile sector, and power plant controls including the EPA NO*_x_* State Implementation Plan Call implemented between 2003 and 2004 (Kim et al., 2006; Russell et al., 2012; Hidy et al., 2014; Foley et al., 2015; Lu et al., 2015). In the United States, NO*_x_* emissions are expected to continue to decrease and reach 72 and 61 % of their 2011 levels in 2018 and 2025, respectively (Eyth et al., 2014). Furthermore, recent work indicates that NO*_x_* emissions may be overestimated in models for the United States (Travis et al., 2016) particularly for on-road gasoline vehicles (McDonald et al., 2012).

Globally, the Representative Concentration Pathway trajectories indicate that NO*_x_* emissions will decrease below year 2000 levels by the middle of the 21st century (Lamarque et al., 2011). Europe has experienced declines in NO*_x_* with NO_2_ concentrations decreasing by 20 % over western Europe between 1996 and 2002 (Richter et al., 2005) and decreasing by an additional ~ 20 % in the more recent past (2004–2010) (Castellanos and Boersma, 2012). In contrast, NO*_x_* emissions in China have increased by large amounts since 1996 (Richter et al., 2005; Stavrakou et al., 2008; Verstraeten et al., 2015) with a more recent leveling out or decrease of NO_2_ concentrations (Krotkov et al., 2016). NO_2_ concentrations in India have continued to increase (Krotkov et al., 2016; Duncan et al., 2016).

These large past and expected future changes in anthropogenic NO*_x_* emissions indicate that analysis of historical data could reveal how NO*_x_* emissions affect organic aerosol formation and more specifically SOA from NO_3_-initiated chemistry. Long-term monitoring networks often measure NO*_x_* and OC, which could allow for correlation analysis. In addition, air quality trends in organic aerosol from traditionally less-sampled locations (e.g., Streets et al., 2008) and emissions for locations such as China have been characterized and could be used for analysis.

In addition to examining measurement data for relationships between NO_3_-derived SOA and NO*_x_*, chemical transport modeling with emission sensitivity simulations can be used to provide estimates of how various SOA pathways respond to changes in NO*_x_* emissions. For example, Carlton et al. (2010b) used the CMAQ model to determine that controllable NO*_x_* emissions were responsible for just over 20 % of total SOA in the United States based on the NO_3_-BVOC mechanism available at the time. Pye et al. (2015) predicted nitrate-derived SOA concentrations would decrease by 25 % due to a 25 % reduction in NO*_x_* emissions, but the overall change including all organic aerosol components would be only 9 % as a result of other less sensitive (or increasing) components. Other modeling studies (Lane et al., 2008; Zheng et al., 2015; Fisher et al., 2016) have shown that total organic aerosol or particle-phase organic nitrates may not respond strongly to decreased NO*_x_* emissions, but significant spatial and composition changes can occur.

### 3.4 Organic nitrate standards

The CIMS technique allows for highly time-resolved, chemically speciated measurements of multifunctional organic nitrates (Beaver et al., 2012; Paulot et al., 2012; Lee et al., 2014a; Xiong et al., 2015). Synthesis, purification, and independent quantification of an individual, isomerically specific organic nitrate is, however, required for calibration because standards are not commercially available, except for a few monofunctional alkyl nitrates.

The synthesis of monofunctional alkyl nitrates can be performed via several methods (Boschan et al., 1955), including nitration of alkyl halides with silver nitrate, direct nitration of alcohols or alkanes with nitric acid (Luxenhofer et al., 1996; Woidich et al., 1999), or treatment of alcohols with dinitrogen pentoxide (Kames et al., 1993). Techniques for the synthesis of multifunctional nitrates (in particular, hydroxynitrates) have been described in previous reports (Muthuramu et al., 1993; Kastler and Ballschmiter, 1998; Werner et al., 1999; Treves et al., 2000). Carbonyl nitrates have also been synthesized using the same protocol, i.e., nitration of hydroxy ketones with dinitrogen pentoxide (Kames et al., 1993; Suarez-Bertoa et al., 2012).

Most recently, three isomers of isoprene hydroxynitrates were synthesized (Lockwood et al., 2010; Lee et al., 2014b). As the precursor ingredient is an organic epoxide on which hydroxy and nitrate functional groups are attached, the same protocol (Nichols et al., 1953; Cavdar and Saracoglu, 2008) can be applied to synthesize hydroxynitrates of various VOC backbones assuming availability of precursor compounds. Oxidation of a single-parent compound can yield numerous isomerically unique byproducts possessing various functional groups, including one or more nitrates. As such, synthesis of and calibration for each nitrate rapidly become prohibitive. Given that multifunctional organic nitrates possessing more than four oxygen atoms, for which synthesis protocols currently do not exist, dominate the particulate nitrate mass of submicron particles (Lee et al., 2016), a more comprehensive calibration technique is needed. Three broad approaches are currently utilized. One is to cryogenically collect a suite of oxidation byproducts (present in the atmosphere, formed in a simulation chamber or flow tube, etc.) on a GC column. The desorbing eluent, separated in time by volatility/polarity as it is thermally desorbed, is measured simultaneously by CIMS and a quantitative instrument such as the TD-LIF (Day et al., 2002; Lee et al., 2014b). The corresponding eluting peaks detected by both instruments allow for calibration of each surviving, isobarically unique (at least for unit mass resolution spectrometers) organic nitrate (Bates et al., 2014; Schwantes et al., 2015; Teng et al., 2015). The second approach employed for the iodide adduct ionization technique is to deduce the instrument response from a comparison of the binding energies of the numerous iodide organic nitrate clusters to those of compounds with known sensitivities by applying variable voltages in the ion molecule reaction region to break up charged clusters systematically. The rate at which the signal of an organic nitrate cluster decays with voltage is a function of its binding energy, which governs its transmission efficiency through the electric fields and thus its sensitivity (Lopez-Hilfiker et al., 2016). Lastly, quantum chemical calculations of specific compounds allow the determination of the sensitivity of their iodide adduct (Iyer et al., 2016) and CF_3_O^−^ (Kwan et al., 2012; Paulot et al., 2012) ionizations.

### 3.5 Critical needs for models

#### 3.5.1 Robust and efficient representation of gas-phase chemistry

Previous sections have detailed the reactions of BVOC with NO_3_ and the need to include this chemistry to represent more accurately processes that control O_3_ and SOA formation. But applying that information in a way that can be used for air quality studies presents a serious challenge. As highlighted in Sect. 2.6.1, the chemical mechanisms currently being used in AQMs are limited in their representation of NO_3_-BVOC chemistry, largely lumping all monoterpenes together, and with no agreement on yields. The lack of detail in current mechanisms is reflected in the variety of methods by which SOA formation from BVOC-NO_3_ chemistry is estimated (Sect. 2.6.2).

Typically, the NO_3_-BVOC chemistry is implemented in AQMs into the existing system of organic and inorganic chemical reactions that occur in the atmosphere. Because there may be hundreds or thousands of different chemical reactions occurring simultaneously and the computational efforts required to solve those on a 3-D grid are onerous, the chemical mechanisms used in AQMs are typically condensed to a certain extent. The greatest challenges in modeling the reactions initiated by NO_3_ and BVOC in AQMs are (1) deciding how much detail must be included to accurately represent the chemistry; (2) estimating intermediate reactions and/or products when direct experimental observations are not available; (3) integrating the new reactions into existing chemical mechanisms; and (4) validating the complete schemes against observational data.

Including all of the attack pathways and isomers that are formed in the reactions of NO_3_ and BVOC and their subsequent products rapidly becomes an intractable problem, as the number of species and reactions produced from a VOC grows exponentially with the number of carbons in the compound (Aumont et al., 2005), resulting in an estimate of almost 400 million products from a single C_10_ hydrocarbon. Even restricting the chemistry solely to the RO_2_ formed from *α*-pinene, *β*-pinene, and limonene via addition of NO_3_ to the double bond results in 861 unique product species and 2646 reactions as estimated from the MCM (http://mcm.leeds.ac.uk/MCM-devel/home.htt; Saunders et al., 2003). In comparison, the chemical mechanisms used in AQMs typically consider a total of 100–200 species and less than 400 reactions to model the entire gas-phase chemistry occurring in the troposphere. One challenge is to find a balance between complexity and computational efficiency that involves both deriving complete mechanisms as well as condensing them to the extent possible.

The second major challenge is that many of the chemical pathways must be estimated given the limited experimental measurements of intermediate reaction rate constants and products. Structure–activity predictions have been used heavily in the past, but these have been formulated for a limited number of compounds. Their predictions become less accurate as the complexity of the molecule increases (Calvert et al., 2015). When heterogeneous reactions play a significant role in the transport and fate of reaction products, as they do in monoterpene chemistry, the challenge becomes even greater. With recent research, new product structures that contribute to SOA have been identified (Boyd et al., 2015). However, these are not covered by existing predictive theory, and these new pathways must be characterized, including reaction rate constants, co-reactants, and products. Physical parameters of all of these new species, such as solubility, radiative properties, emission rates, and deposition velocities also are required, but data are often unavailable for these or even comparable species.

The last challenge is integrating the chemistry within the rest of the chemistry occurring in the atmosphere. The major chemical mechanisms used in AQMs today were developed primarily to address episodes of elevated O_3_ under conditions of high NO*_x_* and have been evaluated for this purpose. Thus, the mechanisms often do not lend themselves well to predicting the chemistry of complex VOC or other air quality endpoints (Kaduwela et al., 2015). Minor pathways with respect to O_3_ formation have been removed from the mechanisms to reduce the computational burden, but these pathways may be important for formation of SOA. In addition, the detailed chemistry of multistep alkoxy and peroxy radical chemistry is condensed into a single step in some mechanisms, but identifying whether these radicals react with NO*_x_* or HO*_x_* or isomerize is critical for predicting the types of organic molecules that are formed. As described in Sect. 2.6, existing mechanisms include the capability for a limited number of nitrates, and in many cases the links to facilitate expansion to more detailed representations are missing.

Significant work must be done to allow modelers to implement this new information in AQMs and thus use this updated knowledge to develop improved predictions of future air quality. One approach is to focus on key chemicals of interest, derive mechanisms that are suitable for specialized applications, and append these on to existing frameworks (for example, Xie et al., 2013). The longer-term view requires a more comprehensive approach that draws on the development of community archives that can better accommodate rapidly changing information and better represent the interactions of biogenic with anthropogenic chemistry. Here, we put forward our recommendations for future work in the following areas:

1. Development of tools for the semi-automated production of the reaction pathways and products of later-generation products resulting from alternate pathways of radical reactions with BVOC. These tools should be able to incorporate experimental data when available. In conjunction with the automated development, we require advanced methods for condensing these large mechanisms into computationally feasible reaction schemes.
2. Improvements in estimation techniques for uncertain pathways, including reaction rate constants for multifunctional stable compounds and radicals for which measurements are not available, and the quantification of the errors associated with these estimation methods.
3. Development of theory and techniques for integrating gas-phase products with SOA production, in this case, describing the transformation of gas-phase organic nitrates to their SOA products.
4. Development of more versatile base mechanisms that have the flexibility to accept increased detail in VOC description and the continuing validation of the complete tropospheric chemical mechanisms against observational data.

#### 3.5.2 Improved techniques and protocols for evaluation of complex and reduced gas-phase mechanisms

Generally speaking, once detailed mechanisms are developed, they are evaluated through some form of benchmarking. Systematic strategies for mechanism evaluation include validation of highly detailed mechanisms unable to be run in 3-D models against benchmark data from well-characterized simulation chamber experiments (Jenkin et al., 1997; Aumont et al., 2005) and the incorporation of these mechanisms into box or 1-D models to validate radical and short-lived species against field campaign observations. Less-detailed air quality (AQ) mechanisms can then be compared to these reference mechanisms by way of sensitivity experiments in idealized modeling studies – often aimed at assessing the sensitivity in O_3_ to changing NO*_x_* and VOC emissions (Archibald et al., 2010; Squire et al., 2015). AQ mechanisms are often also then re-evaluated against chamber and/or field experiment data before they are implemented into 3-D models and then undergo evaluation against extensive measurements in the residual layer.

One of the greatest challenges in the BVOC-NO_3_ system is that current nighttime measurements are mainly collected from surface sites, which are confined to a shallow surface layer at night and not representative of the whole nighttime boundary layer. The impact of nighttime chemistry on daytime ozone and nitrogen/aerosol budget would require careful investigation of nighttime chemistry in the residual layer, which contains > 80 % of air masses at night.

Moreover, the benchmarking activities mentioned above and the development process discussed in Sect. 3.5.1 are not well aligned. A more unified approach that identifies some key mechanistic problems and identifies strategies to evaluate them is required in order to make improved progress on simulating the changing composition of the atmosphere.

#### 3.5.3 Reduce uncertainties in sub-grid-scale processes

Uncertainties in AQM predictions also arise from the representation of physical sub-grid-scale processes. The ones particularly relevant for the NO_3_-BVOC chemistry include, but are not limited to, the following.

##### Nighttime boundary layer mixing

The spatial distribution of BVOC and NO*_x_* precursors is highly variable, but the current AQMs neglect these heterogeneities and assume perfect mixing within grid cells of typically 3–10 km in the horizontal. At those resolutions, models are unable to resolve the localized surface emission sources and the microscale structure of boundary layer turbulence, and therefore cannot resolve spatial heterogeneities in chemistry, partitioning, and mixing of chemicals, which are essential for predicting the concentrations of secondary pollutants.

Typically, the freshly emitted monoterpene species have a tendency to accumulate in the shallow nighttime boundary layer (typically < 200 m), and can react with NO_3_ if available. However, often NO_3_ is located in the residual layer that is decoupled from the nighttime boundary layer (NBL), and the BVOC + NO_3_ reactions would depend on the model’s ability to mix the two layers. Thus, mixing within and out of the boundary layer provides a key challenge for modeling the impacts of BVOC-NO_3_ chemistry, as the measured gradients of NO_3_ and BVOC are very strong in the vertical (e.g., Brown et al., 2007b; Fuentes et al., 2007).

A large focus on model evaluation has been on the impacts of higher horizontal resolution (Jang et al., 1995). It has been shown in several cases that owing to the complex interplay of chemical families, the sensitivity of the chemical system is not captured at lower resolution (e.g., Cohan et al., 2006). However, very little work has focused on the role of improvements in vertical resolution, despite the fact that inter-model differences in properties like the height of the boundary layer vary by over a factor of 2 in some cases (e.g., Hu et al., 2010). Moreover, the NBL is not well mixed, so evaluation of nocturnal physics requires more than just evaluating the NBL height.

##### Plume parameterizations

Typically, parameterizations have been applied to anthropogenic emission sources (e.g., aircraft plumes, urban plumes) and not to biogenic sources. Partly, this is a result of the differences in the source terms, anthropogenic emissions often being well represented as point sources in space, whereas biogenic emissions are often large area sources. However, as the emissions of BVOC are often very species specific, and observations highlight large spatial variability over small areas (e.g., Niinemets et al., 2010), the adoption of the anthropogenic plume parameterizations to BVOC emissions could lead to improvements in model performance.

One approach is the plume-in-grid (PiG) parameterization (Karamchandani et al., 2002). This aims to solve the problem of sub-grid-scale chemical processes by implementing ensembles of Gaussian puffs within the AQM (e.g., Vijayaraghavan et al., 2006). Other approaches include hybrid Eulerian–Lagrangian models (Alessandrini and Ferrero, 2009). These differ from the PiG models by simulating large numbers of stochastic trajectories that can make use of variable reactive volumes to simulate their diffusion into background air masses simulated on Eulerian grids.

Global models have generally used a different approach to the problem of plumes. Broadly, following one of the two paradigms (Paoli et al., 2011) to (i) modify the emissions of the reaction mix (using so-called effective emissions or applying emission conversion factors) and (ii) modify the rates of reaction (effective reaction rates).

### 3.6 Field studies in the developing world and under-studied areas

In light of the questions raised earlier in this review, assessing the role of NO_3_-BVOC chemistry will require field experiments over a wide range of ratios of isoprene to monoterpene emissions and of NO_3_ to BVOC. Future studies of NO_3_-BVOC chemistry are in the planning stages for North America. These studies will provide access to environments with different NO*_x_* levels and over a modest range of isoprene and monoterpene emission rates. A wider range of these parameters can be accessed in countries where NO*_x_* emission controls are not as completely implemented and where BVOC emissions are abundant. Bringing the state-of-the-art capabilities developed for study of NO_3_-BVOC chemistry to locations in China and India would allow insight not only into the role of that chemistry in those countries now but also into the role this chemistry played in Europe and the US prior to implementation of current emission standards. Experiments in the tropics potentially would allow observations of the confluence of BVOC and very low NO*_x_* to be explored, thus providing insight into BVOC-NO_3_ as a sink of NO*_x_*.

### 3.7 Future needs for chamber studies

Field studies, by definition, include the entire complexity of the real atmosphere, so that the identification of single processes and quantification of their impact is challenging. Specific experiments in chambers allow investigating processes without effects from meteorology, which largely impacts observations in the real atmosphere specifically during nighttime, when the lower troposphere is not as well mixed as it is during daytime. In chamber experiments, specific compounds of interest can be isolated and studied under well-controlled oxidation environments, allowing a more detailed and direct characterization of the composition, chemical, and physical properties of aerosols. Because such laboratory chamber data provide the basic understanding for predicting SOA formation, it is important that the design of such experiments mimics the oxidation environments in the atmosphere to the greatest extent possible. Several important needs for understanding NO_3_-BVOC chemistry in chambers include (1) elucidation of kinetic and mechanistic information for NO_3_-BVOC reactions; (2) characterization of wall losses for low-volatility products in the NO_3_-BVOC system; (3) understanding the fate of peroxy radicals in the nighttime atmosphere and its influence on this chemistry; (4) hydrolysis and photooxidation of BVOC-derived organic nitrates from specific BVOC plus specific oxidant pairs over a range of appropriate conditions; (5) optical properties of aerosol organic nitrate; and (6) intercomparison of instrumental methods for key species in the NO_3_-BVOC system.

#### 

##### Kinetic and mechanistic elucidation

The number of chamber studies investigating NO_3_ chemistry is small compared to the number of studies for photochemical oxidation and ozonolysis. In most of the studies, gas-phase oxidation products and SOA yields from the oxidation of BVOC have been measured. Studies include the investigation of SOA from monoterpenes (Wangberg et al., 1997; Griffin et al., 1999; Hallquist et al., 1999; Spittler et al., 2006; Fry et al., 2009, 2011; Boyd et al., 2015; Nah et al., 2016b), methyl butenol (Fantechi et al., 1998a, b), and isoprene (Rollins et al., 2009; Ng et al., 2010; Schwantes et al., 2015). A few more studies investigated gas-phase reaction kinetics, including the reactions of NO_3_ with aldehydes (Clifford et al., 2005; Bossmeyer et al., 2006), amines (Zhou and Wenger, 2013), or cresol (Olariu et al., 2013). As a consequence of the small number of studies, the oxidation mechanisms of organic compounds by NO_3_ and the yields of oxidation products in the gas phase and particle phase have larger uncertainties. The well-controlled oxidation environments in chamber experiments, coupled with complimentary gas-phase and particle-phase measurements (online and offline), allow for elucidating detailed oxidation mechanisms under varying reaction conditions (Ng et al., 2008; Boyd et al., 2015; Schwantes et al., 2015). Identification of gas- and particle-phase reaction products from NO_3_-BVOC chemistry within controlled chamber environments can also greatly aid in the interpretation of field data in which multiple oxidants and BVOC are present. Future chamber experiments will naturally take advantage of new advanced gas–aerosol instrumentation and aim to constrain the formation yields of gas-phase oxidation products and establish a fundamental under-standing of aerosol formation mechanisms from NO_3_-BVOC under a wide range of oxidation conditions.

##### Wall losses

Although chamber studies allow separating processes driven by chemistry and physics from transport processes that occur in the real atmosphere, careful characterization of the behavior of NO_3_ in chambers as well as the organic products of the NO_3_ oxidation remains a research priority. Yields of gas-phase oxidation products can be influenced by chamber-specific loss processes (surface loss on the chamber wall) and SOA yields can be impacted by both direct loss of particles and loss of species that can condense on particle or chamber wall surfaces (McMurry and Grosjean, 1985; Loza et al., 2010; Matsunaga and Ziemann, 2010; Yeh and Ziemann, 2014; Zhang et al., 2014a, 2015; Krechmer et al., 2016; La et al., 2016; Nah et al., 2016a; Ye et al., 2016). The extent to which vapor wall loss affects SOA yields appears to be dependent on the VOC system, from relatively small effects to as high as a factor of 4 (Zhang et al., 2014a; Nah et al., 2016a). Studies on the effects of vapor loss on SOA formation from BVOC + NO_3_ are limited. With minimal or no competing gas–particle partitioning processes, substantial vapor wall loss of organic nitrates has been observed in experiments not specific to NO_3_ oxidation (Yeh and Ziemann, 2014; Krechmer et al., 2016). However, the use of excess oxidant concentrations and rapid SOA formation in BVOC + NO_3_ experiments (hence, shorter experiments) could potentially mitigate the effects of vapor wall loss on SOA yields in chamber studies (Boyd et al., 2015; Nah et al., 2016a). In light of the developing understanding of this issue, an important consideration for the design of any future systematic chamber studies is the influence of vapor wall loss on SOA formation from nitrate radical oxidation under different reaction conditions, such as peroxy radical fates, relative humidity, seeds, oxidant level, chamber volume, etc.

##### Peroxy radical fate

As discussed above, the fate of peroxy radicals directly governs the product distribution in the NO_3_-BVOC system, including SOA yields and composition. Dark reactions of peroxy radicals differ significantly from their photochemical analogs, and are directly related to the development of mechanistic understanding in the NO_3_-BVOC system. There is a need to systematically investigate reaction products and SOA formation from NO_3_-BVOC reactions under different peroxy radical reaction regimes, but this aspect has only recently become a focus of chamber studies (Ng et al., 2008; Boyd et al., 2015; Schwantes et al., 2015). Rapid formation of highly oxygenated organic nitrates has been observed in laboratory studies of *β*-pinene + NO_3_ and *α*-pinene + NO_3_; these products could be formed by unimolecular isomerization of peroxy radicals or autoxidation (Nah et al., 2016b). The importance of this peroxy radical reaction channel in NO_3_-BVOC chemistry warrants further studies. Future chamber studies will need to be explicit in their specification of the peroxy radical chemistry regime that is investigated in a particular experiment, and will need to relate that regime to the conditions of ambient nighttime atmosphere.

##### Organic nitrate hydrolysis and photooxidation

Recent field studies have shown that organic nitrates formed from NO_3_-BVOC are important components of ambient OA. However, the reactivity in both gaseous and condensed phases of these biogenic nitrates, in particular of polyfunctional nitrates, has been subject to few studies and requires better characterization to evaluate the role of these compounds as reservoirs/sinks of NO*_x_*. Field results suggest that the fate of organic nitrates in both the gas and aerosol phase have variable lifetimes with respect to hydrolysis. The difference in the relative amount of primary/secondary/tertiary organic nitrates (which hydrolyze with different rates) from nitrate radical oxidation versus photochemical oxidation needs to be constrained. Most of the hydrolysis studies thus far are conducted in bulk, except for a few recent studies on monoterpene organic nitrates (e.g., Boyd et al., 2015; Rindelaub et al., 2015). The solubility of multifunctional organic nitrates in water and the extent to which hydrolysis occurs in aerosol water warrant future studies. The effect of particle acidity on hydrolysis might also be important for organic nitrates formed in different BVOC systems.

While there are extensive studies on photochemical aging of ozonolysis SOA, studies on photochemical aging of NO_3_-initiated SOA and organic nitrates are extremely limited. A recent study shows that the particle-phase organic nitrates from NO_3_+*β*-pinene and NO_3_+*α*-pinene reactions exhibit completely different behavior upon photochemical aging during the night-to-day transition, and act as permanent and temporary NO*_x_* sinks, respectively (Nah et al., 2016b). With the ~ 1-week lifetime of aerosols in the atmosphere and the majority of NO_3_-BVOC organic nitrates that are formed at night, the photochemical fates of these organic nitrates could impact next-day NO*_x_* cycling and ozone formation. Therefore, there is a critical need to understand the multigenerational chemistry and characterize the evolution of organic nitrates over its diurnal life cycle, including aging NO_3_-initiated SOA and organic nitrates by photolysis and/or OH radicals.

##### Aerosol optical properties

The optical properties, especially in the short wavelength region, of NO_3_-derived SOA may be most conveniently measured during coordinated chamber studies that also include detailed measurements of gas-phase oxidation chemistry and aerosol composition. Such studies could also serve to isolate the specific optical properties of NO_3_-BVOC-derived aerosol to obtain better optical closure in the interpretation of field data. Field studies that include aerosol optical properties measurements in conjunction with other instrumentation can help quantify the bulk organic nitrate abundance and identify organic nitrate molecular composition in the SOA.

##### Instrument intercomparisons

The discussion above shows that recent advances in analytical instrumentation are key to the developing science of NO_3_-BVOC chemistry. Chamber studies provide an excellent opportunity for the comparison and validation of such instrumentation. State-of-the-art and developing instruments for measurement of NO_3_ and N_2_O_5_ were compared approximately a decade ago (Fuchs et al., 2012; Dorn et al., 2013). These instruments have improved and proliferated since that time, and further validation studies are needed. Measurements of total and speciated gas and aerosol-phase organic nitrates, as well as other oxygenated compounds that result from NO_3_-BVOC reactions, have not been the subject of a specific intercomparison study. Their comparison and validation will be a priority in future coordinated chamber studies.

##### Utility of coordinated chamber studies

Because of the need for a better understanding of NO_3_ oxidation and because of the challenges of chamber studies, investigating NO_3_ chemistry, coordination between studies carried out in different chambers, and between chamber and field studies, can augment efforts of single or standalone chamber studies. Coordinated studies that would include several chambers could increase the accuracy and reliability of results and quantify realistic errors associated with product yield estimates. This can be achieved by determining the same quantities in similar experiments in different chambers. Studies could benefit from complementary capabilities and properties of chambers. Chambers that typically operate at higher concentration ranges, and therefore increased oxidation rates, are suitable to perform a larger number of experiments that are useful for screening experiments and a series of experiments with systematic variations of chemical conditions. Other chambers are suited to perform experiments at atmospheric reactant concentrations. Experiments in these chambers may take place on a longer timescale, for example, a scale characteristic of the duration of at least one night. Analytical instrumentation and capability also differs considerably among chambers, so that coordinated chamber studies can make use of the determination of complementary quantities such as product yields of different organic compounds and characterization of various properties of particles for the same chemical system. For instance, it would be invaluable to conduct coordinated studies where a variety of instrument techniques are used to measure total and speciated gas- and particle-phase organic nitrates, as well as aerosol physical and chemical properties in the same chamber.

Substantial insights into aerosol sources, formation, and processing can be gained from coordinated laboratory chamber and field studies. Laboratory chamber experiments provide the fundamental data to interpret field measurements. The analysis of field data in turn can provide important insights for constraining chamber experiment parameters so that the oxidation conditions in chambers can be as representative as possible of those in the atmosphere. Two recent sets of experiments serve as examples of this approach. Fundamental chamber studies on *β*-pinene+NO_3_ in the Georgia Tech Environmental Chamber (GTEC) facility under conditions relevant to the SE US provided constraints on the contribution of monoterpenes + NO_3_ to ambient OA during the 2013 SOAS campaign (Boyd et al., 2015; Xu et al., 2015a). The Focused Isoprene eXperiment at California Institute of Technology (FIXCIT) chamber study following SOAS advanced the understanding of isoprene oxidation chemistry relevant to the SE US (Nguyen et al., 2014). It is important not to consider fundamental laboratory studies as isolated efforts, but they should be an integrated part of field studies. Similarly, having the modeling community involved in early planning stages of laboratory and field studies will greatly aid in the identification of critically needed measurement data.

## 4 Impacts of NO_3_-BVOC chemistry on air quality

The previous sections have demonstrated that understanding how NO_3_ reacts with BVOC, including the ultimate fate of products, encompasses all aspects of atmospheric physics, chemistry, and transport. These sections have raised numerous complex and fascinating scientific questions and highlighted the critical need for much more basic science to fill in unknown aspects of this system. However, “getting this system right” is not just an interesting scientific problem because it has direct implications for policy decisions that governments across the world are taking to protect citizens and ecosystems from harmful effects of air pollutants. Addressing the uncertainties raised in the previous sections is critical for developing efficient, accurate, and cost-effective strategies to reduce the harmful effects of air pollution.

BVOC have long been predicted to be significant contributors to regional and global O_3_ (e.g., Pierce et al., 1998; Curci et al., 2009) and PM_2.5_ (Pandis et al., 1991), with NO_3_ reactions providing a major pathway for loss of ambient BVOC (Winer et al., 1984; Pye et al., 2010; Xie et al., 2013). If BVOC react with NO_3_ instead of OH, the O_3_ production of the BVOC can be reduced relative to reactions through OH, although in some instances they may slightly increase O_3_ by reducing next-day NO*_x_*. For example, measurements in St. Louis (Millet et al., 2016) demonstrate that nights with lower levels of NO_3_ resulted in higher isoprene concentrations the following morning, producing higher and earlier O_3_ peaks. Recent insights into the role of biogenic nitrates, which are produced in large quantities through the reactions of NO_3_ with primary emitted BVOC and subsequent reactions of their stable products, demonstrate that these compounds can substantially alter the availability of NO*_x_* (Perring et al., 2013). This highlights the importance of accurate treatment of fates of organic nitrates that form from nighttime chemistry in models, which will impact the next-day NO*_x_* and ozone levels. Organic nitrates from BVOC + NO_3_ also can contribute to nitrogen deposition (Nguyen et al., 2015), which adversely impacts ecosystems. The ways in which the patterns of deposition for biogenic nitrates affect inorganic nitrate deposition remain poorly understood.

### Implications for spatial distribution of ozone and PM_2.5_

While it is clear that NO_3_-BVOC reactions affect oxidant availability and SOA, it remains unclear how large that role is in the ambient atmosphere relative to other VOC and other oxidants and where it occurs. The extent of O_3_ formation downwind of sources is influenced by the transport of NO*_y_* species, including organic nitrates, which can release NO*_x_* downwind, where O_3_ may be formed more efficiently. Biogenically derived nitrates are the dominant organic nitrates in many places (Pratt et al., 2012). A variety of different organic nitrates are formed from different BVOC, with some being short lived (releasing NO_2_ locally) and others being long lived (releasing NO_2_ downwind unless they are removed in the meantime). Errors in our attribution of the lifetime of individual biogenic nitrate compounds can cause errors in predicted NO*_x_* redistributions regionally and globally, and modify the spatial distributions of O_3_ (Perring et al., 2013). Updates to the chemistry of BVOC-NO_3_ also could alter calculations of the relative role of biogenic species versus anthropogenic pollutants to O_3_ and PM_2.5_ formation.

### Implications for control strategy development

Air quality models are used not only to understand the production of air pollutants in the current atmosphere but also to guide the development of strategies to reduce the future pollution burden. Uncertainties in the chemistry and removal of BVOC can contribute to uncertainties in the sensitivity of O_3_ and PM to emission reduction strategies. This increases the risk of implementing expensive control strategies that are found later to be inefficient (more control specified than needed) or ineffective (do not meet the air quality goals for which they were developed). As noted by Millet et al. (2016), in urban areas downwind of high isoprene emissions, the loss of isoprene by NO_3_ at night can produce the opposite O_3_-NO*_x_* behavior that would normally be expected in urban areas, potentially causing a reassessment of optimum control strategies. In addition, the early O_3_ peaks noted on low NO_3_ nights expands the high ozone time window, resulting in higher 8 h O_3_ averages, on which regulatory compliance in the US is based.

The uncertainties in our understanding of NO_3_-BVOC chemistry propagate into chemical mechanisms, as described in Sect. 3. Past work has shown that vastly different chemical mechanisms may predict similar O_3_ in current atmospheres but show huge differences for intermediate species (e.g., Luecken et al., 2008) and different potential responses to precursor reductions, including different indicators of O_3_ sensitivity to VOC versus NO*_x_* reductions (Knote et al., 2015). The presence of large weekend effects in NO*_x_* makes identifying such errors more likely in current analyses.

Incorporating new information on biogenic chemistry within a chemical mechanism will impact the availability of NO*_x_* (e.g., Archibald et al., 2010; Xie et al., 2013) and modify the predicted effectiveness of anthropogenic NO*_x_* controls. Incorporating new chemical information into models can also impact PM_2.5_ sensitivities to NO*_x_* reductions. In one example, organic PM_2.5_ was almost twice as responsive to a NO*_x_* reduction than in older mechanisms (Pye et al., 2015). Because much of the NO*_x_* dependence of O_3_ and aerosols from NO_3_-BVOC reactions is inadequately accounted for in models, the few examples we have hint that current NO*_x_* control strategies might result in more significant improvements to air quality than currently assumed. Retrospective analyses should focus on elucidating the elements of this hypothesis that are represented in the historical record.

The role of climate change in modifying air quality is also a highly uncertain issue and may be particularly sensitive to the characterization of BVOC. Biogenic emissions may increase or decrease in the future, depending on many factors including increased temperatures, changes in water availability, occurrence of biotic and abiotic stress (e.g., Kleist et al., 2012; Wu et al., 2015), CO_2_ fertilization, CO_2_ inhibition, and land use changes (Chen et al., 2009; Squire et al., 2014). Uncertainties in biogenic reactions may be amplified as they become a larger share of the VOC burden in some places. The predicted response of O_3_ to future climate has been found to be especially sensitive to assumptions about the chemical pathways of BVOC reactions, in particular the treatment of nitrates. Mao et al. (2013) and several earlier researchers found that predictions of the O_3_ response to NO*_x_* reductions change from negative to positive depending solely on how the isoprene chemistry was represented. Similarly, a comparison of several widely used chemical mechanisms with varied descriptions of BVOC-derived nitrates (Squire et al., 2015) found that description of BVOC chemistry significantly alters not only the amount of oxidant change predicted under future scenarios but also the direction of the change. Direct measurements of the key steps in isoprene oxidation should eliminate the ambiguity in such model calculations. Nonetheless, the exquisite sensitivity of model predictions of ozone trends to the representation of isoprene and NO*_x_* indicates that ambient observations of those trends are an excellent strategy for evaluating the accuracy of mechanisms.

The relative distribution of emissions among different types of BVOC may also shift as climate and land use changes, emphasizing the need to understand differences among terpenes in their chemistry, transport, and fate (Pratt et al., 2012). While most of the research to date has been done on isoprene, with some on *α*-pinene and *β*-pinene, little has been done on products or reaction parameters of other terpenes. The previous sections have demonstrated that different terpenoid structures can have vastly different atmospheric chemistry and physical properties, so it is unclear whether assuming one “representative” species or distribution, as is done in most chemical mechanisms, will adequately account for future impacts of BVOC on O_3_ and PM.

### Summary of impacts

This review has illustrated that accurate characterization of NO_3_-BVOC chemistry is critical to our understanding of both the air quality and climate impacts of NO*_x_* emissions. Our knowledge of the complexity of NO_3_-BVOC reaction pathways and multigenerational products has advanced rapidly, especially in the last decade. Despite the fact that much of that information is not yet in a form that can be included in current air quality models, we anticipate improved predictive capabilities in models in the coming years through sustained laboratory and field studies coupled to model development. While the current levels of uncertainty make it difficult to accurately quantify the impact of NO_3_-BVOC chemistry on air pollutant concentrations, we expect that developments in this field will improve the effectiveness of air pollution control strategies going forward. The limited studies available demonstrate that even small changes to BVOC chemistry modify the production of oxidants (NO_3_, OH, and O_3_) and change the transport of NO*_y_*. Therefore, NO_3_-BVOC oxidation modifies the chemical regime in which additional BVOC oxidation occurs. Of most importance will be the studies that indicate changes in the direction of predicted future pollutant concentrations as chemical mechanisms of BVOC are updated. Emission control strategies and attainment of air quality goals rely on the best possible chemical models. Current and future laboratory and field research is critical to the improvement of chemical mechanisms that account for biogenic chemical processes and products which will augment efforts to reduce harmful air pollutants.

## Supplementary Material

supp

## Figures and Tables

**Table 1:** Reaction rate constants of NO_3_+ BVOC.

Compound	*k*(NO_3_+BVOC) (cm^3^ molecule^−1^ s^−1^)^a^	Temperature (K)	Technique/reference

Isoprene 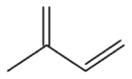	(5.94 ± 0.16)×10^−13^	295	RR/(Atkinson et al., 1984)
	(1.30 ± 0.14)×10^−12^	298	DF-MS/(Benter and Schindler, 1988)
	(3.03 ± 0.45)×10^−12^exp[−(450 ± 70)/T]	251–281	F-LIF/(Dlugokencky and Howard, 1989)
	(6.52 ± 0.78)×10^−13^	297	F-LIF/(Dlugokencky and Howard, 1989)
	(1.21 ± 0.20)×10^−12^	298	RR/(Barnes et al., 1990)
	(7.30 ± 0.44)×10^−13^	298	DF-MS/(Wille et al., 1991)
	(8.26 ± 0.60)×10^−13^	298	DF-MS/(Wille et al., 1991)
	(1.07 ± 0.20)×10^−12^	295	PR-A/(Ellermann et al., 1992)
	(6.86 ± 0.55)×10^−13^	298	RR/(Berndt and Boge, 1997b)
	(7.3 ± 0.2)×10^−13^	298	F-CIMS/(Suh et al., 2001)
	(6.24 ± 0.11)×10^−13^	295	RR/(Zhao et al., 2011b)
	**6.5×10^−13^ (Δlog *k*: ± 0.15)**	**298**	**IUPAC**

α-pinene 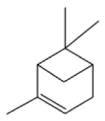	(5.82 ± 0.16)×10^−12^	295	RR/(Atkinson et al., 1984)
	(1.19 ± 0.31)×10^−12^exp[(490 ± 70)/T]	261–383	F-LIF/(Dlugokencky and Howard, 1989)
	(6.18 ± 0.94)×10^−12^	298	F-LIF/(Dlugokencky and Howard, 1989)
	(6.56 ± 0.94)×10^−12^	298	RR/(Barnes et al., 1990)
	(3.5 ± 1.4)×10^−13^exp[(841 ± 144)/T]	298–423	DF-LIF/(Martinez et al., 1998)
	(5.9 ± 0.8)×10^−12^	298	DF-LIF/(Martinez et al., 1998)
	(5.82 ± 0.56)×10^−12^	298	RR/(Kind et al., 1998)
	(4.88 ± 0.46)×10^−12^	298	RR/(Stewart et al., 2013)
	**6.2×10^−12^ (Δlog *k* : ± 0.1)**	**298**	**IUPAC**

β-pinene 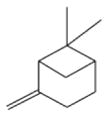	(2.36 ± 0.10)×10^−12^	295	RR/(Atkinson et al., 1984)
	(2.38 ± 0.05)×10^−12^	296	RR/(Atkinson et al., 1988)
	(1.1 ± 0.4)×10^−12^	298	RR/(Kotzias et al., 1989)
	(2.81 ± 0.47)×10^−12^	298	RR/(Barnes et al., 1990)
	(1.6 ± 1.5)×10^−10^exp[(−1248 ± 36)/T]	298–293	DF-LIF/(Martinez et al., 1998)
	(2.1 ± 0.4)×10^−12^	298	DF-LIF/(Martinez et al., 1998)
	(2.81 ± 0.56)×10^−12^	298	RR/(Kind et al., 1998)
	**2.5×10^−12^ (Δlog *k* : ± 0.12)**	**298**	**IUPAC**

Sabinene 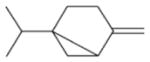	(1.01 ± 0.03)×10^−11^	296	RR/(Atkinson et al., 1990)
	(1.07 ± 0.16)×10^−11^	298	DF-LIF/(Martinez et al., 1999)
	(2.3 ± 1.3)×10^−10^exp[(−940 ± 200)/T]	298–393	DF-LIF/(Martinez et al., 1999)
	**1.0×10^−11^ (Δlog *k* : ± 0.15)**	**298**	**IUPAC**

Camphene 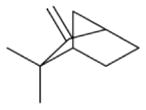	(6.54 ± 0.16)×10^−13^	296	RR/(Atkinson et al., 1990)
	(3.1 ± 0.5)×10^−12^exp[(−481 ± 55)/T]	298–433	DF-LIF/(Martinez et al., 1998)
	(6.2 ± 2.1)×10^−13^	298	DF-LIF/(Martinez et al., 1998)

2-carene 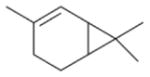	(1.87 ± 0.11)×10^−11^	295	RR/(Corchnoy and Atkinson, 1990)
	(2.16 ± 0.36)×10^−11^	295	RR/(Corchnoy and Atkinson, 1990)
	(1.66 ± 0.18)×10^−11^	298	DF-LIF/(Martínez et al., 1999)
	(1.4 ± 0.7)×10^−12^exp[(741 ± 190)/T]	298–433	DF-LIF/(Martínez et al., 1999)
	**2.0×10^−11^ (Δlog *k* : ± 0.12)**	**298**	**IUPAC**

3-carene 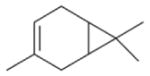	(1.01 ± 0.02)×10^−11^	295	RR/(Atkinson et al., 1984)
	(8.2 ± 1.2)×10^−11^	298	RR/(Barnes et al., 1990)
	**9.1×10^−11^ (Δlog *k* : ± 0.12)**	**298**	**IUPAC**

Δ-limonene 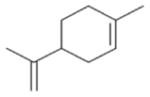	(1.31 ± 0.04)×10^−11^	295	RR/(Atkinson et al., 1984)
	(1.12 ± 0.17)×10^−11^	298	RR/(Barnes et al., 1990)
	(9.4 ± 0.9)×10^−12^	298	DF-LIF/(Martínez et al., 1999)
	**1.2×10^−11^ (Δlog *k* : ± 0.12)**	298	**IUPAC**

α-phellandrene 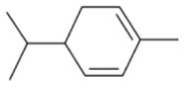	(8.52 ± 0.63)×10^−11^	294	RR/(Atkinson et al., 1985)
	(5.98 ± 0.20)×10^−11^	298	RR/(Berndt et al., 1996)
	(4.2 ± 1.0)×10^−11^	298	DF-LIF/(Martínez et al., 1999)
	(1.9 ± 1.3)×10^−9^exp[−(1158 ± 270)/T]	298–433	DF-LIF/(Martínez et al., 1999)
	**7.3×10^−11^ (Δlog *k* : ± 0.15)**	**298**	**IUPAC**

β-phellandrene 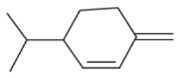	(7.96 ± 2.82)×10^−12^	297	RR/(Shorees et al., 1991)

α-terpinene 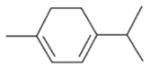	(1.82 ± 0.07)×10^−10^	294	RR/(Atkinson et al., 1985)
	(1.03 ± 0.06)×10^−10^	298	RR/(Berndt et al., 1996)
	**1.8×10^−10^ (Δlog *k* : ± 0.25)**	**298**	**IUPAC**

γ-terpinene 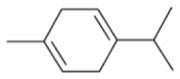	(2.94 ± 0.05)×10^−11^	294	RR/(Atkinson et al., 1985)
	(2.4 ± 0.7)×10^−11^	298	DF-LIF/(Martínez et al., 1999)
	**2.9×10^−11^ (Δlog *k* : ± 0.12)**	**298**	**IUPAC**

Terpinolene 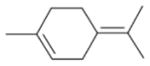	(9.67 ± 0.51)×10^−11^	295	RR/(Corchnoy and Atkinson, 1990)
	(5.2 ± 0.9)×10^−11^	298	DF-LIF/(Martínez et al., 1999)
	(6.12 ± 0.52)×10^−11^	298	RR/(Stewart et al., 2013)
	**9.7×10^−11^ (Δlog *k* : ± 0.25)**	**298**	**IUPAC**

Ocimene (*cis, trans*) 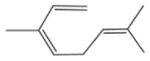	(2.23 ± 0.06)×10^−11^	294	RR/(Atkinson et al., 1985)
	**2.2×10^−11^ (Δlog *k* : ± 0.15)**	**298**	**IUPAC**

Myrcene 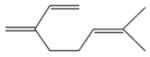	(1.06 ± 0.02)×10^−11^	294	RR/(Atkinson et al., 1985)
	(1.28 ± 0.11)×10^−11^	298	DF-LIF/(Martínez et al., 1999)
	(2.2± 0.2)×10^−12^exp[(523 ± 35)/T]	298–433	DF-LIF/(Martínez et al., 1999)
	**1.1×10^−11^ (Δlog *k* : ± 0.12)**	**298**	**IUPAC**

α-cedrene 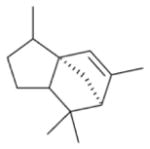	(0.82 ± 0.30)×10^−11^	296	RR/(Shu and Atkinson, 1995)

α-copaene 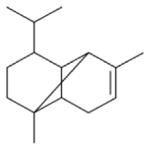	(1.6 ± 0.6)×10^−11^	296	RR/(Shu and Atkinson, 1995)

β-caryophyllene 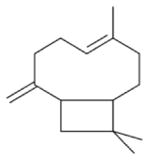	(1.9 ± 0.8)×10^−11^	296	RR/(Shu and Atkinson, 1995)

α-humulene 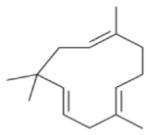	(3.5 ± 1.3)×10^−11^	296	RR/(Shu and Atkinson, 1995)

Longifolene 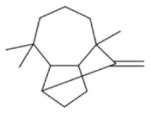	(6.8 ± 2.1)×10^−13^	296	RR/(Shu and Atkinson, 1995)

Isolongifolene 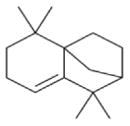	(3.9 ± 1.6)×10^−12^	298	RR/(Canosa-Mas et al., 1999b)

Alloisolongifolene 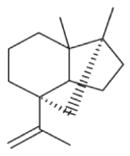	(1.4 ± 0.7)×10^−12^	298	RR/(Canosa-Mas et al., 1999b)

α-neoclovene 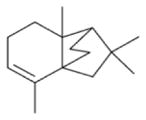	(8.2 ± 4.6)×10^−12^	298	RR/(Canosa-Mas et al., 1999b)

2-methyl-3-buten-2-ol 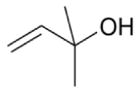	4.6×10^−14^exp[−(400 ± 35)/T]	267–400	F-A/(Rudich et al., 1996)
	(1.21 ± 0.09)×10^−14^	298	F-A/(Rudich et al., 1996)
	(2.1 ± 0.3)×10^−14^	294	DF-A/(Hallquist et al., 1996)
	(1.55 ± 0.55)×10^−14^	294	RR/(Hallquist et al., 1996)
	(8.7 ± 3.0)×10^−14^	298	RR/(Fantechi et al., 1998b)
	(1.0 ± 0.2)×10^−14^	297	RR/(Noda et al., 2002)
	(1.1 ± 0.1)×10^−14^	297	RR/(Noda et al., 2002)
	**1.2×10^−14^ (Δlog *k* : ± 0.2)**	**298**	**IUPAC**

3-methyl-2-buten-1-ol 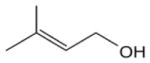	(1.0 ± 0.1)×10^−12^	297	RR/(Noda et al., 2002)

3-methyl-3-buten-1-ol 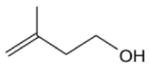	(2.7 ± 0.2)×10^−13^	297	RR/(Noda et al., 2002)

*cis*-3-hexen-1-ol 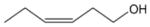	(2.72 ± 0.83)×10^−13^	296	RR/(Atkinson et al., 1995)
	(2.67 ± 0.42)×10^−13^	298	DF-CEAS/(Pfrang et al., 2006)

*trans*-3-hexen-1-ol 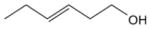	(4.43 ± 0.91)×10^−13^	298	DF-CEAS/(Pfrang et al., 2006)

*cis*-4-hexen-1-ol 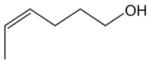	(2.93 ± 0.48)×10^−13^	298	DF-CEAS/(Pfrang et al., 2006)

*trans*-2-hexen-1-ol 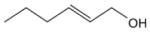	(1.30 ± 0.24)×10^−13^	298	DF-CEAS/(Pfrang et al., 2006)

*cis*-2-hexen-1-ol 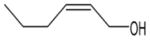	(1.56 ± 0.24)×10^−13^	298	DF-CEAS/(Pfrang et al., 2006)

*trans*-2-hexenal 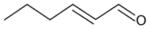	(1.21 ± 0.44)×10^−14^	296	RR/(Atkinson et al., 1995)
	(1.36 ± 0.29)×10^−14^)	295	RR/(Zhao et al., 2011b)
	(4.7 ± 1.5)×10^−15^	294	AR/(Kerdouci et al., 2012)

4-methylenehex-5-enal 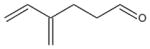	(4.75 ± 0.35)×10^−13^	296	RR/(Baker et al., 2004)

(3Z)-4-methylhexa-3,5-dienal 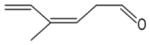	(2.17 ± 0.30)×10^−12^	296	RR/(Baker et al., 2004)

(3E)-4-methylhexa-3,5-dienal 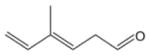	(1.75 ± 0.27)×10^−12^	296	RR/(Baker et al., 2004)

4-methylcyclohex-3-en-1-one 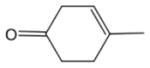	(1.81 ± 0.35)×10^−12^	296	RR/(Baker et al., 2004)

*cis*-3-hexenyl acetate 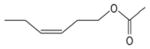	(2.46 ± 0.75)×10^−13^	296	RR/(Atkinson et al., 1995)

Methyl vinyl ketone 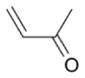	< 1.2×10^−16^	298	F-A/(Rudich et al., 1996)
	< 6×10^−16^	296	DF- RR/(Kwok et al., 1996)
	(3.2 ± 0.6)×10^−16^	296	LIF/(Canosa-Mas et al., 1999a)
	(5.0 ± 1.2)×10^−16^	296	RR/(Canosa-Mas et al., 1999a)
	**< 6×10^−16^**	**298**	**IUPAC**

Methacrolein 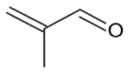	(4.46 ± 0.58)×10^−15^	296	RR/(Kwok et al., 1996)
	(3.08 ± 0.18)×10^−15^	298	RR/(Chew et al., 1998)
	(3.50 ± 0.15)×10^−15^	298	RR/(Chew et al., 1998)
	(3.72 ± 0.47)×10^−15^	296	RR/(Canosa-Mas et al., 1999a)
	**3.4×10^−15^ (Δlog *k* : ± 0.15)**	**298**	**IUPAC**

Pinonaldehyde 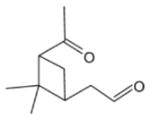	(2.40 ± 0.38)×10^−14^	299	RR/(Hallquist et al., 1997a)
	(6.0 ± 2.0)×10^−14^	300	RR/(Glasius et al., 1997)
	(2.0 ± 0.9)×10^−14^	296	RR/(Alvarado et al., 1998)
	**2.0×10^−14^ (Δlog *k* : ± 0.25)**	**298**	**IUPAC**

Linalool 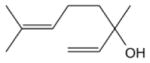	(1.12 ± 0.40)×10^−11^	296	RR/(Atkinson et al., 1995)

α-terpineol 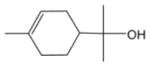	(1.6 ± 0.4)×10^−11^	297	RR/(Jones and Ham, 2008)

Sabinaketone 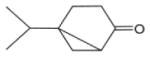	(3.6 ± 2.3)×10^−16^	296	RR/(Alvarado et al., 1998)

Caronaldehyde 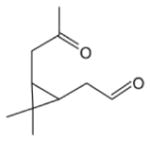	(2.5 ± 1.1)×10^−14^	296	RR/(Alvarado et al., 1998)

a Given uncertainties are those provided by the authors of the kinetic studies. The procedures used to calculate them are not detailed here, as they often differ from one study to another. Readers are referred to the original papers for more information on the uncertainties’ determination.

RR: relative rate; DF-MS: discharge flow–mass spectrometry; DF-LIF: discharge flow–laser–induced fluorescence; DF-A: discharge flow–absorption; DF-CEAS: discharge flow–cavity–enhanced absorption spectroscopy; F-LIF: flow system–laser–induced fluorescence; F-CIMS: flow system–chemical ionization mass spectrometry; F-A: flow system–absorption; PR-A: pulse radiolysis–absorption; AR: absolute rate in simulation chamber.

**Table 2:** Oxidation products and SOA yields observed in previous studies of NO_3_-BVOC reactions. Except where noted, carbonyl and organic nitrate molar yields represent initial gas-phase yields measured by FTIR spectroscopy (carbonyl and organic nitrate) or thermal desorption laser-induced fluorescence (TD-LIF) (organic nitrate only; Rollins et al., 2010; Fry et al., 2013). In some cases, the ranges reported correspond to wide ranges of organic aerosol loading, listed in the rightmost column. Where possible, the mass yield at 10 μg m^−3^ is reported for ease of comparison.

BVOC	Carbonyl molar yield	Organic nitrate molar yield	SOA mass yield	Corresponding OA loading or other relevant information
Isoprene		62–78 % (Rollins et al., 2009)	2 % (14 % after further oxidation) (Rollins et al., 2009)	Nucleation (1 μg m^−3^)
			4–24 % (Ng et al., 2008)	3–70 μg m^−3^; 12 % at 10 μg m ^−3^

*α*-pinene	58–66 % (Wangberg et al., 1997); 69–81 % (Berndt and Boge, 1997a); 65–72 % (Hallquist et al., 1999); 39–58 % (Spittler et al., 2006)	14 % (Wangberg et al., 1997); 12–18 % (Berndt and Boge, 1997b); 18–25 % (Hallquist et al., 1999); 11–29 % (Spittler et al., 2006); 10 % (Fry et al., 2014)	0.2–16 % (Hallquist et al., 1999)	Nucleation; 0.5 % at 10 ppt N_2_O_5_ reacted, 7 % at 100 ppt N_2_O_5_ reacteda
			4 or 16 % (Spittler et al., 2006)	Values for 20 % RH and dry conditions, respectively, at *M*_∞_^b^
			1.7–3.6 % (Nah et al., 2016a)	1.2–2.5 μg m^−3^
			0 % (Fry et al., 2014)	Both nucleation and ammonium sulfate seeded
			9 % (Perraud et al., 2010)	Nucleation at 1 ppm N_2_O_5_ and 1 ppm *α*-pinene; OA is 480 μg m^−3^ assuming density is1.235 g cm^−3^

*β*-pinene	0–2 % (Hallquist et al., 1999)	51–74 % (Hallquist et al., 1999); 40 % (Fry et al., 2009); 22 % (Fry et al., 2014); 45–74 % of OA mass (Boyd et al., 2015)	32–89 % (Griffin et al., 1999)	32–470 μg m^−3^ ; low end closest to 10 μg m^− 3^
			7–40 % (Moldanova and Ljungstrom, 2000) using new model to reinterpret data from Hallquist et al. (1999) (10–52 %)	7–10 % at 7 ppt N_2_O_5_ reacted; 40–52 % at 39 ppt N_2_O_5_ reacted
			50 % (Fry et al., 2009)	40 μg m^−3^; same yield at both 0 and 60 % RH
			33–44 % (Fry et al., 2014)	10 μg m^−3^ c
			27–104 % (Boyd et al., 2015)	5–135 μg m^−3^, various seeds and RO_2_ fate regimes; 50 % for experiments near 10 μg m ^−3^

Δ-carene	0–3 % (Hallquist et al., 1999)	68–74 % (Hallquist et al., 1999); 77 % (Fry et al., 2014)	13–72 % (Griffin et al., 1999)	24–310 μg m^−3^ ; low end closest to 10 μg m^− 3^
			12–49 % (Moldanova and Ljungstrom, 2000) using new model to reinterpret data from Hallquist et al. (1999) (15–62 %)	7–395 ppt N_2_O _5_ reacted; 12–15 % at 6.8 ppt N_2_O_5_ reacted
			38–65 % (Fry et al., 2014)	10 μg m^−3^ c

Limonene	69 % (Hallquist et al., 1999); 25–33 % (Spittler et al., 2006)	48 % (Hallquist et al., 1999); 63–72 % (Spittler et al., 2006); 30 % (Fry et al., 2011); 54 % (Fry et al., 2014)	14–24 % (Moldanova and Ljungstrom, 2000) using new model to reinterpret data from Hallquist et al. (1999) (17 %)	10 ppt N_2_O_5_ reacted; higher number in Moldanova and Ljungstrom (2000) from an additional injection of 7 ppt N_2_O_5_ and accounting for secondary reactions
			21 or 40 % (Spittler et al., 2006)	Ammonium sulfate or organic seed, respectively, at *M*_∞_^b^
			25–40 % (Fry et al., 2011)	Nucleation to 10 μg m^−3^ (second injection of oxidant)
			44–57 % (Fry et al., 2014)	10 μg m^−3^ c

Sabinene			14–76 % (Griffin et al., 1999)	24–277 μg m^−3^ ; low end closest to 10 μg m^− 3^
			25–45 % (Fry et al., 2014)	10 μg m^−3^ c

*β*-caryophyllene			91–146 % (Jaoui et al., 2013)	60–130 μg m^−3^ ; low end closest to 10 μg m^− 3^
			86 % (Fry et al., 2014)	10 μg m^−3^

a The authors assume that N_2_O_5_ reacted is equal to BVOC reacted. The anomalously low 0.2 % yield observed at 390 ppt N_2_O_5_ reacted is a lower limit; Hallquist et al. note that the number–size distribution for that experiment fell partly outside the measured range.

b *M*_∞_ corresponds to extrapolated value at highest mass loading. Organic seed aerosol in these experiments was generated from O_3_ + BVOC. Full dataset was shown only for limonene, where asymptote is 400 μg m^−3^.

c Yield range corresponds to two different methods of calculating ΔBVOC.

**Table 3:** **(a)**Selected CRDS and CEAS instruments used to quantify NO_3_ mixing ratios in ambient air. **(b)** Selected instruments used to quantify NO_3_ and N_2_O_5_ mixing ratios in ambient air other than by cavity-enhanced absorption spectroscopy.

(a)		
Principle of measurement (laser pulse rate)	LOD or precision (integration time)	Reference
BB-CEAS	2.5 pptv (8.6 min)	Ball et al. (2004)
BB-CRDS	1 pptv (100 s)	Bitter et al. (2005)
Off-axis cw CRDS (500 Hz)	2 pptv (5 s)	Ayers and Simpson (2006)
On-axis pDL-CRDS (33 Hz)	<1 pptv (1 s)	Dubé et al. (2006)
BB-CEAS	4 pptv (60 s)	Venables et al. (2006)
pDL-CRDS (10 Hz)	2.2 pptv (100 s)	Nakayama et al. (2008)
Off-axis cw CRDS (200 Hz)	2 pptv (5 s)	Schuster et al. (2009), Crowley et al. (2010)
CE-DOAS	6.3 pptv (300 s)	Platt et al. (2009), Meinen et al. (2010)
BB-CEAS	2 pptv (15 s)	Langridge et al. (2008), Benton et al. (2010)
BB-CEAS	< 2 pptv (1s)	Kennedy et al. (2011)
On-axis cw-CRDS (500 Hz)	<1 pptv (1 s)	Wagner et al. (2011)
On-axis cw-CRDS (300 Hz)	8 pptv (10 s)	Odame-Ankrah and Osthoff (2011)
BB-CEAS	1 pptv (1 s)	Le Breton et al. (2014)
BB-CEAS	7.9 pptv (60 s)	Wu et al. (2014)

(b)			
Principle of measurement	LOD or precision e(integration time)	Species detected	Reference
MIESR	< 2 pptv (30 min)	NO_3_	Geyer et al. (1999)
CIMS	12 pptv (1 s)	NO_3_ + N_2_O_5_	Slusher et al. (2004)
LIF	11 pptv (10 min)	NO_3_	Matsumoto et al. (2005a), Matsumoto et al. (2005b)
LIF	28 pptv (10 min)	NO_3_	Wood et al. (2005)
CIMS	30 pptv (30 s)	N_2_O_5_	Zheng et al. (2008)
CIMS	5 pptv (1 min)	N_2_O_5_	Kercher et al. (2009)
CIMS	7.4 pptv (1 s)	N_2_O_5_	Le Breton et al. (2014)
CIMS	39 pptv (6 s)	N_2_O_5_	Wang et al. (2014)

CEAS = cavity-enhanced absorption spectroscopy; CRDS = cavity ring-down spectroscopy; BB = broadband; pDL = pulsed dye laser; CE-DOAS = cavity-enhanced differential optical absorption spectroscopy; cw = continuous-wave diode laser. MIESR = matrix isolation electron spin resonance; CIMS = chemical ionization mass spectrometry; LIF = laser-induced fluorescence; LOD = limit of detection.

**Table 4:** Gas-phase organic nitrate yields (in percent) from BVOC + NO_3_ systems in current chemical mechanisms. Gas-phase organic nitrate yields depend on RO_2_ fate as indicated in the ternary diagrams; clockwise from the top: RO_2_ reacts with NO_3_, RO_2_, and HO_2_.

Chemical mechanism	Gas-phase yield of organic nitrates from isoprene+NO_3_	Gas-phase yield of organic nitrates from monoterpenes+NO_3_	References
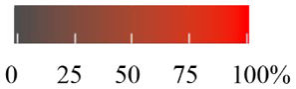			
CB05	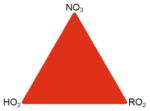	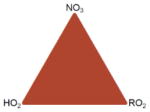	(Yarwood et al., 2005)
CB6r2	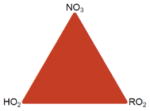	same as CB05	(Perring et al., 2009; Hildebrandt Ruiz and Yarwood, 2013)
GECKO-A	up to 100% (see supporting information)	same as isoprene	(Aumont et al., 2005)
GEOS-Chem v10-01	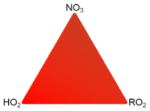	NA (monoterpene oxidation is offline)	(Mao et al., 2013)
GFDL AM3	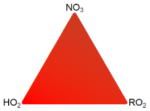	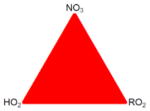	
MCM v3.3.1	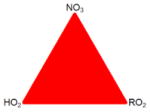	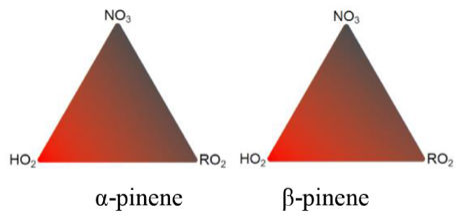	(Jenkin et al., 1997; Saunders et al., 2003; Jenkin et al., 2015)
MOZART	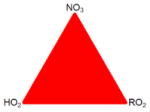	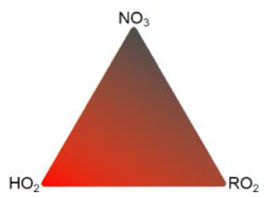	(Emmons et al., 2010) with updates on organic nitrates
SAPRC07	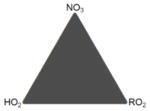	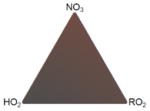	(Carter, 2010b; Carter, 2010a) Plots of RO_2_+RO_2_ based on RO2C
SAPRC07tic	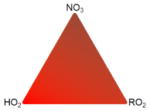	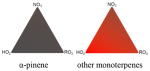	(Rollins et al., 2009; Xie et al., 2013) α-pinene (same as SAPRC07) Other monoterpenes: (Pye et al., 2015)

**Table 5:** Treatment of SOA formation from BVOC-NO_3_ systems in current 3-D models.

Model	Gas-phase chemistry	Isoprene + NO_3_ parameterization	Monoterpene + NO_3_ parameterization
CAMx v6.20 with SOAP	CB05, CB6, or SAPRC99	No SOA from this path	NO_3_ SOA yields same as photooxidation (OH + ozone) yields^1^
CAMx v6.20 with 1.5-D VBS	CB05, CB6, or SAPRC99	NO_3_ SOA yields same as photooxidation (OH + ozone) yields^2^	NO_3_ SOA yields same as photooxidation (OH + ozone) yields^2^
CMAQ v5.1 cb05e51-AERO6	CB05 with additional modification^3^	Odum two-product approach based on Kroll et al. (2006) photooxidation (OH) yields^3^	Odum two-product approach based on Griffin et al. (1999a) photooxidation (OH + ozone) yields^4^
CMAQ v5.1 SAPRC07tc-AERO6	SAPRC07^5^ with two monoter- penes: *α*-pinene (APIN) and other monoterpenes (TERP)	Odum two-product approach based on Kroll et al. (2006) photooxidation (OH) yields^3^	Odum two-product approach based on Griffin et al. (1999a) photooxidation (OH + ozone) yields^4^
CMAQ v5.1 SAPRC07tic-AERO6i	SAPRC07tic^6^,^7^	based on semivolatile organic nitrate from isoprene dinitrate^8^	no SOA from *α*-pinene + NO_3_; SOA from other monoterpenes based on semivolatile organic nitrates^8^
EURAD-IM	RACM	Odum two-product approach 9	Odum two-product approach 10 with *T* dependence^11^,^12^
GEOS-Chem v10-01	GEOS-Chem v10-01 with speciated isoprene nitrates^6^,^7^	VBS fit^9^,^13^	VBS fit to *β*-pinene + NO_3_ experiment^10^,^13^
GFDL AM3	GFDL AM3	no SOA from this pathway	Odum two-product approach based on *β*-pinene + NO_3_^10^,^14^
GISS-GCM II	NA (offline oxidants)	no SOA from this pathway	Odum two-product approach based on *β*-pinene + NO_3_^10^,^14^
GLOMAP/ UKESM-1	VOC + NO_3_	Based on Kroll et al. experiments (2006), set to 3 %^15^	Based on Tunved et al. (2004), set to 13 %^15^
STOCHEM-CRI	MCM	CRI species fit to MCMv3.1 simulations^16^,^17^,^18^	CRI species fit to MCMv3.1 simulations 16,17,18
WRF-Chem v3.6.1	MOZART-MOSAIC	no SOA from this pathway	VBS fit to *β*-pinene + NO_3_ experiment^10^,^19^

1 Strader et al. (1999).

2 Koo et al. (2014).

3 Appel et al. (2016).

4 Carlton et al. (2010a).

5 Hutzell et al. (2012).

6 Rollins et al. (2009).

7 Xie et al. (2013).

8 Pye et al. (2015).

9 Ng et al. (2008).

10 Griffin et al. (1999).

11 Li et al. (2013).

12 Kiendler-Scharr et al. (2016).

13 Pye et al. (2010).

14 Chung and Seinfeld (2002).

15 Scott et al. (2014).

16 Utembe et al. (2009).

17 Johnson et al. (2006).

18 Khan et al. (2015).

19 Knote et al. (2014).

## Appendix A

Glossary of abbreviations and common chemical formulas.

ACSM: Aerosol chemical speciation monitor
AM3: Atmospheric Model 3
AMS: Aerosol mass spectrometry/spectrometer
AQM: Air quality model
AR: Absolute rate in simulation chamber
BB-CEAS: Broadband cavity-enhanced absorption spectroscopy
BB-CRDS: Broadband cavity ring-down spectroscopy
BDE: Bond dissociation energy
BEACHON-RoMBAS: Rocky Mountain Biogenic Aerosol Study
BERLIOZ: Berliner Ozonexperiment
BEWA: Regional Biogenic Emissions of Reactive Volatile Organic Compounds from Forests
BC: Black carbon
BrC: Brown carbon
BVOC: Biogenic volatile organic compound(s)
CalNex: California Research at the Nexus of Air Quality and Climate Change
CAMx: Comprehensive Air Quality Model with extensions
CARES: Carbonaceous Aerosol and Radiative Effects Study
CAPRAM: Chemical aqueous-phase radical mechanism
CAPS: Cavity-attenuated phase shift spectroscopy/spectrometer
CB05: Carbon Bond 2005 chemical mechanism
CCN: Cloud condensation nuclei
CEAS: Cavity-enhanced absorption spectroscopy/spectrometer
CE-DOAS: Cavity-enhanced differential optical absorption spectroscopy
CIMS: Chemical ionization mass spectrometry/spectrometer
CMAQ: Community Multiscale Air Quality
CRDS: Cavity ring-down spectroscopy/spectrometer
DF-A: Discharge flow – absorption
DF-CEAS: Discharge flow – cavity-enhanced absorption spectroscopy
DF-LIF: Discharge flow – laser-induced fluorescence
DF-MS: Discharge flow – mass spectrometry
DMS: Dimethyl sulfide
DOAS: Differential optical absorption spectroscopy/spectrometer
ELVOC: Extremely low volatility organic compounds
EMEP: European Monitoring and Evaluation Program
EPA: Environmental Protection Agency
ESI: Electrospray ionization
EUCAARI: European Integrated project on Aerosol, Cloud, Climate, and Air Quality Interactions
EURAD-IM: EURopean Air pollution Dispersion – Inverse Model
F-A: Flow system – absorption
F-CIMS: Flow system – chemical ionization mass spectrometry
F-LIF: Flow system – laser-induced fluorescence
FIGAERO: Filter inlet for gases and aerosols
FIXCIT: Focused Isoprene eXperiment at California Institute of Technology
FTICR: Fourier transform ion cyclotron
FTIR: Fourier transform infrared spectroscopy
GC: Gas chromatography
GC-MS: Gas chromatography mass spectrometry
GCM: Global climate model
GECKO-A: Generator of Explicit Chemistry and Kinetics of Organics in the Atmosphere
GEOS-Chem: Goddard Earth Observing System – Chemistry
GLOMAP: Global Model of Aerosol Processes
GTEC: Georgia Tech Environmental Chamber
HAP: Hazardous air pollutant(s)
HOHPEX: HOHenpeissenberg Photochemistry Experiment
HPLC: High-performance liquid chromatography
HO_2_: Hydroperoxy radical
HR-ToF: High-resolution time-of-flight
IC: Ion chromatography
ICARTT: International Consortium for Atmospheric Research on Transport and Transformation
IGAC: International Global Atmospheric Chemistry
IN: Ice nuclei
IUPAC: International Union of Pure and Applied Chemistry
LC: Liquid chromatography
LED: Light-emitting diode
LIF: Laser-induced fluorescence
LO-OOA: Less-oxidized oxygenated organic aerosol
LV-OOA: Low-volatility oxygenated organic aerosol
MAC: Mass absorption coefficient
MCM: Master chemical mechanism
MBO: 2-methyl-3-buten-2-ol
MIESR: Matrix isolation electron spin resonance
MO-OOA: More-oxidized oxygenated organic aerosol
MOSAIC: Model for Simulating Aerosol Interactions and Chemistry
MOZART: Model for OZone and Related chemical Tracers
NARSTO: North American Research Strategy for Tropospheric Ozone
NEAQS: New England Air Quality Study
NBL: Nocturnal boundary layer
NEI: National Emissions Inventory
NO: Nitric oxide
NO_2_: Nitrogen dioxide
NO_3_: Nitrate radical
N_2_O_5_: Dinitrogen pentoxide
NO*_x_*: Nitrogen oxides, NO + NO_2_
NO*_y_*: Total reactive nitrogen
NOS: Nitrooxy organosulfate
NSF: National Science Foundation
O_3_: Ozone
OA: Organic aerosol
OC: Organic carbon
OH: Hydroxyl radical
OS: Organosulfate
PACIFIC: Pacific Air Quality Study
PAHs: Polyaromatic (or polycyclic aromatic) hydrocarbons
PiLS: Particle-into-liquid sampler
PiG: Plume-in-grid
PM: Particulate matter
PM_1_*_/_*_2.5_: Particulate matter smaller than 1*/*2.5 μ
PMF: Positive matrix factorization
PROPHET: Program for Research on Oxidants: Photochemistry, Emissions and Transport
PTR-MS: Proton transfer reaction mass spectrometry
PR-A: Pulse radiolysis – absorption
RH: Relative humidity
RI: Refractive index
RL: Residual layer
RONO_2_: Organic nitrate
RO_2_: Organic peroxy radical
RR: Relative rate
SAPRC: Statewide Air Pollution Research Center
SAR: Structure activity relationship
SEAC^4^RS: Studies of Emissions and Atmospheric Composition, Clouds and Climate Coupling by Regional Surveys
SEARCH: Southeastern Aerosol Research and Characterization
SOA: Secondary organic aerosol
SOAS: Southern Oxidant and Aerosol Study
SOS: Southern Oxidant Study
STOCHEM-CRI: STOchastic lagrangian CHEMistry model using Common Representative Intermediates
SV-OOA: Semi-volatile oxygenated organic aerosol
TD-CAPS: Thermal dissociation cavity-attenuated phase shift spectroscopy
TD-CRDS: Thermal dissociation cavity ring-down spectroscopy
TD-LIF: Thermal dissociation laser-induced fluorescence
UKESM-1: UK Earth System Model 1
VBS: Volatility basis set
VOC: Volatile organic compound(s)
WRF-Chem: Weather Research and Forecasting Model with Chemistry
