# Supplementary material for: Nitrate radicals and biogenic volatile organic compounds: oxidation, mechanisms, and organic aerosol
Source: Atmos Chem Phys. Author manuscript; Available in PMC 2018 Aug 22. (PMC6104845; doi:10.5194/acp-17-2103-2017)
Supplement: supp [file NIHMS982684-supplement-supp.pdf]

Supplement of Atmos. Chem. Phys., 17, 2103–2162, 2017
http://www.atmos-chem-phys.net/17/2103/2017/
doi:10.5194/acp-17-2103-2017-supplement
© Author(s) 2017. CC Attribution 3.0 License.

Atmospheric Chemistry and Physics
Open Access

Supplement of

Nitrate radicals and biogenic volatile organic compounds: oxidation, mechanisms, and organic aerosol

Nga Lee Ng et al.

Correspondence to: Nga Lee Ng (ng@chbe.gatech.edu) and Steven S. Brown (steven.s.brown@noaa.gov)

The copyright of individual parts of the supplement might differ from the CC-BY 3.0 licence.

Supplementary Info to section 2.3

The lifetime (τ) of a single component, liquid organic particle with respect to loss by reaction with NO_3 at concentration $[NO_3]$ is partially governed by the uptake coefficient (γ) (Robinson et al., 2006;Gross et al., 2009b):

$$\tau_{liquid} = \frac{2\rho_{\mathrm{org}} N_{\mathrm{A}} D_{\mathrm{p}}}{3M_{\mathrm{org}}\bar{c}\gamma[\mathrm{NO_3}]} \quad (1)$$

Where D_p is the particle diameter, ρ_{org} and M_{org} are the density and molecular weight of the organic component, respectively and $\bar{c}$ is the mean molecular velocity of gas-phase NO_3. Thus defined, τ is the time required for all the organic molecules in a spherical (i.e. liquid) particle to be oxidised once. The lifetimes of some particle bound, low volatility organics can be reduced to seconds at night time due to the reaction of NO_3 whilst daytime lifetimes are significantly longer (Kaiser et al., 2011).

For a solid particle, the following expression has been used (Gross et al., 2009a)

$$\tau_{solid} = \frac{N_{\mathrm{tot}}}{\bar{c}\gamma[\mathrm{NO_3}]} \quad (2)$$

Where N_{tot} is the number of organic molecules on the surface.

For a reactive organic coating on a non-reactive core the following expression has been used to derive the uptake coefficient:

$$\gamma = \frac{(R_p^3 - R_c^3)\rho_{org} N_A \eta_{org}}{3M_{org} R_p D\bar{C}t} \quad (3)$$

Where R_p and R_c are the radii of the particle and the non-reactive core, respectively. η_{org} is the ratio of consumption of the organic species per NO_3 reaction, D is the diffusion coefficient of NO_3 to the particle and t is the average NO_3 exposure (molecule cm^{-3} s^{-1}).

If the reaction takes place throughout the particle, the uptake of NO_3 may be parameterised in terms of bulk accommodation and reaction within a resistor framework (Hanson et al., 1994):

$$\frac{1}{\gamma} = \left\{\frac{1}{\alpha_b} + \frac{\bar{c}}{4HRT(D_b k'_{org})^{0.5}}\right\} \quad (4)$$

Where α_b is the bulk accommodation coefficient, H is the solubility, D_b is the bulk-phase diffusion coefficient of NO_3 and k'_{org} is the pseudo first order rate coefficient for reaction of NO_3 with the organic component of interest and is usually proportional to its concentration.

For aqueous particles and surfaces, the diffusion of NO_3 though the liquid is characterised by a value of D_b which is close to 1×10^{-5} cm^2 s^{-1}. For gas uptake by amorphous (semi-)solid organics, inhomogeneous mixing with strong concentration gradients is expected in the bulk phase. In such case, more complex depth-resolved kinetic models (Pöschl et al., 2007;Pfrang et al., 2010;Shiraiwa et al., 2010;Shiraiwa et al., 2012;Roldin et al., 2014) are required that treat bulk diffusion and chemical reactions at the surface and in the bulk to fully evaluate the uptake and reaction of NO_3. The key kinetic input parameters include the second-order surface and bulk reaction rate coefficients between NO_3 and organics (see section 2c2), the bulk diffusion coefficients of NO_3 and organics as well as the surface accommodation coefficient of NO_3 (α_s), desorption lifetime of NO_3 (τ_d), and Henry's law coefficient for NO_3 ($K_{sol,cc}$). Typical ranges for these parameters the interactions between organic surfaces and NO_3 are: $\alpha_s = 10^{-3} - 1$, $\tau_d = 10^{-10} - 1$, and $K_{sol,cc} = 10^{-4} - 10^{-3}$ mol cm^{-3} atm^{-1} (Shiraiwa et al., 2012). These parameters can be estimated by quantum chemical calculations such as molecular dynamic simulations and density functional theory. Otherwise, they need to be constrained by fitting experimental data using the depth-resolved kinetic models.

Table S1 Summary of experimental measurements of uptake coefficients and products formed in the interaction of NO_3 with organic surfaces.

Organic surface or particle	Methods	γ (10^{-3})	Products / Comments	Reference
**Alkanes**				
n-hexadecane [1]	CWFT, (*l*)	2.6 ± 0.8 (293 K)	Uptake coefficient greatly reduced for frozen samples. **N1**	(Moise et al., 2002)
n-hexadecane [2]	CWFT, (SAM)	14 ± 2 (293 K)	RH ~0 %, In the absence of O_2	(Knopf et al., 2006)
squalane [3]	CWFT, (*l*)	5.2 ± 0.4 (278 K)		(Xiao and Bertram, 2011)
squalane [4]	AFT, in N_2 VUV-MS	< 7.8 (293 K)	100 nm diameter particles of pure squalene. Gas-phase NO_2 observed at close to unit yield.	(Lee et al., 2013)
1-octadecanethiol	CWFT, (SAM) IR, SIMS	298 K	$RONO_2$ formation on the surface observed. **N2**	(Gross and Bertram, 2009)
1-octadecanethiol [5]	CWFT, (SAM) XPS,	0.88 ± 0.225 (298 K)	Conversion of alkane to oxidized, condensed phase organics.	(Knopf et al., 2006)
heptamethyl nonane [6]	CWFT, (*l*)	2.1 ± 0.8 (253 K)	Uptake coefficient greatly reduced for frozen samples. **N1**	(Moise et al., 2002)
**Saturated alcohols and carbonyls**				
n-octanoic [8]	CWFT, (*l*)	2.1 ± 0.3 (290 K)		(Moise et al., 2002)
1-octanol [9]	CWFT, (*l*)	7.1 ± 1.6 (258 K)		(Moise et al., 2002)
glycerol [10]	CWFT, (*l*)	0.8 – 1.7 (268-293 K)		(Gross and Bertram, 2009)
16-mercapto-1-hexadecanol [11]	UHV, (SAM) RAIRS	6 ± 1 **N3**	Organic nitrates formed simultaneously with loss of terminal CH_2OH groups.	(Zhang and Morris, 2015)
Levoglucosan [12]	CWFT, (*s*)	~1.4 (298 K) **N4**	RH < 0.5 %, O_2 present.	(Knopf et al., 2011) (Shiraiwa et al., 2009)
Diethyl sebacate (DES) [13]	CWFT, (*l*)	3.5 – 4.5 (278-298 K)		(Gross and Bertram, 2009)
Diethyl sebacate (DES) [14]	CWFT, (*l*)	4.4 ± 0.4 (278 K)		(Xiao and Bertram, 2011)
Dioctyl sebacate (DOS) [15]	CWFT, (*l*)	3.9 ± 0.3 (278 K)		(Xiao and Bertram, 2011)
Tridecanal (in DES)	CWFT, (*l*)	~1.4-12 (275 K)	~0.06-0.27 mol L^{-1} tridecanal	(Iannone et al., 2011)
Tridecanal (in DOS)	CWFT, (*l*)	~0.5-5 (275 K)	~0.02-0.11 mol L^{-1} tridecanal	(Iannone et al., 2011)
Tridecanal (in squalene)	CWFT, (*l*)	~0-1 (275 K)	~0.02-0.1 mol L^{-1} tridecanal	(Iannone et al., 2011)

**Alkenes**				
1-octadecene [17]	CWFT, (*l*)	1.6 ± 0.3 (293 K)		(Moise et al., 2002)
1-hexadecene [18]	CWFT, (*l*)	2.3 ± 0.9 (277 K)		(Moise et al., 2002)
7-tetradecene [19]	CWFT, (*l*)	5.8 ± 2.0 (246 K)		(Moise et al., 2002)
Methyl oleate [20]	CWFT, (*l*)	140^{+860}_{-50} (278 K)		(Xiao and Bertram, 2011)
Methyl oleate (in DES)	CWFT, (*l*)	15-95 (278 K)	~ 0.3- 2.3 wt % methyl oleate	(Xiao and Bertram, 2011)
Methyl oleate (in DOS)	CWFT, (*l*)	15 – 30 (278 K)	~ 0.6 – 2.6 wt % methyl oleate	(Xiao and Bertram, 2011)
methyl oleate (in squalane)	CWFT, (*l*)	10-25 (278 K)	~ 0.7 – 3.1 wt % methyl oleate	(Xiao and Bertram, 2011)
Undec-10-ene-1-thiol [21]	CWFT, (SAM)	34^{+44}_{-18} (298 K)	γ decreased (> factor 10) with reaction time as number of double bonds were depleted. Condensed phase products were organonitrates and carbonyls.	(Gross and Bertram, 2009)
Squalene [22]	AFT, in N_2 (*lp*) VUV-MS	180 ± 30 (293 K)	γ (calculated assuming that only NO_3 was lost to surface) increased (to 820 ± 110) with reaction time.	(Lee et al., 2013)
17-octadecene-1-thiol [23]	UHV, (SAM) RAIRS, XPS	2.3 ± 0.5	Reaction at terminal double bond to form organic nitrate via addition. **N3**	(Zhang et al., 2014c)
**Unsaturated Acids**				
Abietic [25]	CWFT, (*s*)	~3 (298 K)	RH < 0.5 % **N5**	(Knopf et al., 2011) (Shiraiwa et al., 2012)
Linoleic (conj.) [26]	CWFT, (*l*)	7.9 ± 1.2 (273 K)		(Moise et al., 2002)
Linoleic (unconj.) [27]	CWFT, (*l*)	15.0 ± 2.0 (288 K)		(Moise et al., 2002)
Linoleic (conj.) [28]	AFT, (*lp*)	1010 ± 180 (295 K)		(Zhao et al., 2011)
Linoleic [29]	CWFT, (*l*)	400^{+450}_{-310} (298 K)	RH ~ 0 %, In the absence of O_2	(Knopf et al., 2011)
Linoleic (unconj.) [30]	AFT, (*lp*)	530 ± 120 (295 K)		(Zhao et al., 2011)
Linoleic (conj.) [31]	CWFT, (*l*)	>120 (278-298 K)		(Gross et al., 2009a)
Linoleic (unconj.) [32]	CWFT, (*l*)	>130 (288 K)		(Gross et al., 2009a)
Linolenic [33]	AFT, (*lp*)	720 ± 170 (295 K)		(Zhao et al., 2011)
Oleic	ATR-IR, GC-MS, LC-MS (*l*)	(298 K)	Products are carbonyl compounds which contain $-ONO_2$, $-O_2NO_2$ and $-NO_2$ functional groups. Also high molecular weight products formed.	(Hung et al., 2005)
Oleic [34]	AFT, (*lp*)	270 ± 60 (295 K)	In the presence of O_2, products are organo-nitrate (acid) with alcohol and carbonyl functional groups.	(Zhao et al., 2011)
Oleic [35]	AFT, (*lp*)	140 ± 30	Experiment conducted at room temperature	(Renbaum-Wolff et al., 2013)
Oleic [36]	CWFT, (*l*)	290^{+710}_{-210} (288 K)	RH ~0 %, In the absence of O_2	(Knopf et al., 2011)
Oleic [37]	CWFT, (*l*)	>70 (285-302 K)		(Gross et al., 2009a)
**Aromatics and PAHs**				

Pyrene	Coated Glass Fibre Filter (*s*)		Formation (yield of ~ 100%) of 1-nitropyrene attributed to reaction with N_2O_5.	(Pitts et al., 1984)
Pyrene [39]	CWFT, (*s*)	>100 (293 K)		(Atkinson et al., 1995)
Pyrene [40]	CWFT, (*s*)	380^{+620}_{-300} (273 K)<br>790^{+210}_{-670} (293-297 K)	Initial uptake coefficient on a fresh surface. γ decreased with NO_3 exposure at 263 K but not at 297 K. HNO_3 and NO_2 observed as gas-phase products.	(Gross and Bertram, 2008b)
Pyrene	XPS, DRIFTS, GC-MS		Pyrene on a Teflon disc. Nitropyrene observed and attributed to reaction with NO_3 /and/or N_2O_5.	(Atkinson et al., 1990)
Pyrene	AFT, (*lp*)<br>VUV-AMS<br>GC-MS		Pyrene coated on an azelaic acid particle.<br>Bulk samples coated on glass. Main product is 1-nitro-pyrene. **N6**	(Atkinson et al., 1988)
Pyrene [41]	Chamber, (*lp*)<br>VUV-AMS	370 (287 K)	Pyrene coated on an azelaic acid particle. 1-nitropyrene. **N7**	(Atkinson et al., 1984)
Pyrene	Chamber, (*lp*)<br>VUV-AMS<br>GC-MS		2-nitropyrene formed at RH = 0.5 %, 1-nitropyrene observed at RH = 43 % **N8**	(Atkinson et al., 1985;Zhang et al., 2014a)
Benz[a]pyrene	10L glass bulb<br>AMS		Nitrated benz[a]pyrene. **N9**	(Lu et al., 2011)
1-nitro-pyrene [42]	Chamber, (*lp*)<br>VUV-AMS	60 (287 K)	1-nitro-pyrene coated on an azelaic acid particle. 1,3-, 1,6-, and 1,8-dinitropyrenes observed. **N7**	(Atkinson et al., 1984)
1-hydroxy-pyrene [43]	Chamber, (*lp*)<br>VUV-AMS	570 (287 K)	1-hydroxy-pyrene coated on an azelaic acid particle. **N7**	(Atkinson et al., 1984)
Anthracene	AFT, (*lp*)<br>VUV-AMS<br>GC-MS		Anthracene coated on an azelaic acid particle. Bulk samples coated on glass. Main product is 9-nitro-anthracene. **N10**	(Atkinson et al., 1988)
Anthracene	XPS, DRIFTS, GC-MS XPS,		Anthracene on a Teflon disc. 9-Nitroanthracene observed and attributed to reaction with NO_3 /and/or N_2O_5.	(Atkinson et al., 1990)
Benz[a]anthracene [44]	CWFT, (*s*)	59^{+110}_{-49} (273 K)<br>130^{+530}_{-96} (293-297 K)	Initial uptake coefficient on a fresh surface. γ decreased with NO_3 exposure rapidly at 263 K, more slowly at 297 K.	(Gross and Bertram, 2008b)
Benz[a]anthracene	AFT, (*lp*)<br>VUV-AMS<br>GC-MS		Benz[a]anthracene coated on an azelaic acid particle. Bulk samples coated on glass. Products are benz[a]anthracene-7,12-dione and mono-nitrobenz[a]anthracene.	(Atkinson et al., 1988)
Benz[a]anthracene [45]	Chamber, (*lp*)<br>VUV-AMS	290 (287 K)	Benz[a]anthracene coated on an azelaic acid particle. 7-Nitrobenzo(a)anthracene and benzo(a)anthracene-7,12-dione observed. **N7**	(Atkinson et al., 1984)
Perylene	Coated Glass Fibre Filter Filter (*s*)		No reaction products attributable to N_2O_5 or NO_3 uptake observed.	(Baker et al., 2004)

Fluoranthene [46]	CWFT, (*s*)	87^{+280}_{-63} (273 K)	Initial uptake coefficient on a fresh surface. γ decreased with NO_3 exposure at 263 K but not at 297 K.	(Gross and Bertram, 2008b)
Fluoranthene	Chamber, (*lp*)<br>VUV-AMS<br>GC-MS		2-nitro-fluoranthene formed at RH = 0.5 %<br>1-,3-, 7-, and 8-nitrofluoranthene observed at RH = 43 %, **N8**	(Zhang et al., 2014b)
Phenanthrene	AFT, (*lp*)<br>VUV-AMS<br>GC-MS		Phenanthrene coated on an azelaic acid particle. Bulk samples coated on glass. Main products are mono-nitrophenanthracenes. **N11**	(Atkinson et al., 1988)
Carbaryl	AFT, (*lp*)<br>VUV-AMS		Carbaryl coated on an azelaic acid particle. Initial product is (nitro-1-naphthyl) M-methylcarbamate. **N12**	(Barnes et al., 1990)
Syringaldehyde [47]	Chamber, (*lp*)<br>VUV-AMS<br>GC-MS	330	Syringaldehyde coated on an azelaic acid particle. 2,6-dimethoxybenzoquinone produced. seen as product following H-abstraction by NO_3. **N13**	(Atkinson et al., 1984)
Vanillic acid [48]	Chamber, (*lp*)<br>VUV-AMS	310	Vanillic acid coated on an azelaic acid particle. 4,6-dinitrogaiacol and protocatechuic acid acid observed. **N14**	(Atkinson et al., 1984)
Coniferyl aldehyde [49]	Chamber, (*lp*)<br>VUV-AMS	280	Coniferyl aldehyde coated on an azelaic acid particle. Products observed formed via a combination of abstraction and addition reactions of NO_3. **N15**	(Atkinson et al., 1984)
Chysrene [50]	Chamber, (*lp*)<br>VUV-AMS<br>GC/MS	180 (287 K)	Chrysene coated on an azelaic acid particle. 6-nitrochrysene and dinitrochyrsene observed. **N7**	(Atkinson et al., 1984)
Triphenylene	AFT, (*lp*)<br>VUV-AMS<br>GC-MS		Triphenylene coated on an azelaic acid particle. Bulk samples coated on glass. Products are 1-nitroreiphenylene (major) and 2-nitrotriphenylene.	(Benter and Schindler, 1988)
Nitroguaiacol [51]	CWFT, (*s*)	~22 (298 K) **N16**	RH < 0.5 %	(Knopf et al., 2011)
Deuterated PAHs	Filter<br>GC-MS		Equal parts mixture of deuterated Pyrene, Fluoranthene and Triphenylene. Deuterated 1-nitro-pyrene and 8-nitro-fluoranthene were major products. **N10**	(Berndt and Boge, 1997)
Ambient PAHs	Filter<br>GC-MS		Collection of ambient particles and subsequent exposure to NO_3/N_2O_5. Nitropyrenes, nitrofluoranthenes, anthracene, nitro-chrysene and nitrobenzo(a)pyrene observed.	(Berndt and Boge, 1997)
**Other**				
SOA (limonene)	Chamber , (*lp*)	50-200 (295 K)	γ derived by modelling NO_3 and particle composition	(Fry et al., 2011)

Numbering: The number in square brackets following the organic name refers to the number on the x-axis of Figure 3 in the manuscript.

Surface state: *l*= liquid, *lp* = liquid particle, *s* = solid, SAM = self-assembled monolayer.

Methods: AFT = Aerosol flow tube, IR = Infra-red, ATR = Attenuated total reflection, CWFT = coated wall flow tube, SIMS = secondary ion mass spectrometry, VUV-AMS = vacuum ultraviolet photoionisation aerosol mass spectrometry, UHV = ultra high vacuum chamber, RAIRS = reflection absorption infrared spectroscopy, XPS = X-ray photoelectron spectroscopy.

Chemicals: 1-octadecanethiol is $C_{18}H_{38}S$. Abeitic acid ($C_{20}H_{30}O_2$) is Abieta-7,13-dien-18-oic acid. Anthracene is $C_{14}H_{10}$ (Tricyclo[8.4.0.0^{3,8}]tetradeca-1,3,5,7,9,11,13-heptaene). Anthrone is $C_{14}H_{10}O$ (10*H*-Anthracen-9-one). Benzo[a]anthracene is $C_{18}H_{12}$. Benzo[a]pyrene is $C_{20}H_{12}$. Carbaryl is $C_{12}H_{11}NO_2$ (1-naphthyl methylcarbamate). Chyrsene ($C_{18}H_{12}$) is 1,2-Benzophenanthrene. Coniferyl aldehyde, $C_{10}H_{10}O_3$, is a ^{methoxyphenol} ((Z or E)-3-(4-hydroxy-3-methoxyphenyl)prop-2-enal). DES (di-ethyl sebacate) is $C_2H_5OC(O)(CH_2)_8C(O)OC_2H_5$). DOS (di-octyl sebacate) is $(CH_2)_8(C(O)OC_8H_{17})_2$). Fluoranthene is $C_{16}H_{10.}$ Levoglucosan ($C_6H_{10}O_5$) is (1*R*,2*S*,3*S*,4*R*,5*R*)-6,8-Dioxabicyclo[3.2.1]octane-2,3,4-triol. Linoleic acid is $C_{18}H_{32}O_2$ ((9*Z*,12*Z*)-9,12-octadecadienoic acid). Linoleic acid (conj.) is a mixture of cis- and trans-9,11 octadecadienoic acid and -10,12 octadecadienoic acid. Linolenic acid is $C_{18}H_{30}O_2$ ((9*Z*,12*Z*,15*Z*)-9,12,15-octadecatrienoic acid). Methyl oleate is $CH_3(CH_2)_7CH{=}CH(CH_2)_7CO_2CH_3$. Nitroguaiacol ($C_7H_7NO_4$) is 2-methoxy-5-nitrophenol. Oleic acid is $C_{18}H_{34}O_2$, ((9*Z*)-Octadec-9-enoic acid). PAHs = polycyclic aromatic hydrocarbons. Phenanthrene is $C_{14}H_{10}$ (Tricyclo[8.4.0.0^{2,7}]tetradeca-1,3,5,7,9,11,13-heptaene). Pyrene is $C_{16}H_{10}$ (benzo[def]phenanthrene). SOA (limonene) is secondary organic aerosol formed by reaction with limonene with both O_3 and NO_3. Squalane is $C_{30}H_{62}$ (2,6,10,15,19,23-Hexamethyltetracosane). Squalene (triterpene with 6 double bonds) is (6*E*,10*E*,14*E*,18*E*)-2,6,10,15,19,23-hexamethyltetracosa-2,6,10,14,18,22-hexaene. Syringaldehyde, $C_9H_{10}O_4$, is a methoxyphenol (4-Hydroxy-3,5-dimethoxybenzaldehyde). Triphenylene ($C_{18}H_{12}$) is 9,10-benzophenanthrene. Undec-10-ene-1-thiol is $C_{11}H_{22}S$. Vanillic acid, $C_8H_8O_4$, is a methoxyphenol (4-Hydroxy-3-methoxybenzoic acid).

Comments: **N1**. n-hexadecane: Uptake coefficient decreased by a factor ~7 when the surface was frozen at 283-289 K, which was attributed to the reduced rate of diffusion of NO_3 into the bulk sample. **N2**. Reaction proceeds via abstraction to form an alkyl radical, R which reacts with O_2 to form RO_2. The peroxy radical reacts with NO_3 to form RO, which is converted to $RONO_2$ via reaction with NO_2 present. **N3**. Experiment in UHV (no O_2 present). Derivation of γ required calculation of NO_3 concentration close to the surface from a N_2O_5 dosing pressure and the NO_2-NO_3-N_2O_5 equilibrium. **N4**. Uptake coefficient increased (to ~ 3×10^{-3}) when the NO_3 concentration was reduced but decreased (~ factor of 10) with exposure time as reactive sites were depleted on the solid surface of levoglucosan. The NO_3 concentration- and time-dependence of the uptake coefficient could be described with a multilayer model of surface and bulk reactions and bulk diffusion of NO_3 with a diffusion coefficient of $10^{-8} – 10^{-7}$ cm^2 s^{-1}. **N5**. Uptake coefficient increased (to ~ 6×10^{-3}) when the NO_3 concentration was reduced but decreased (~ factor of 10) with exposure time as reactive sites were depleted on the solid surface of abeitic acid. The NO_3 concentration- and time-dependence of the uptake coefficient could be described with a multilayer model of surface and bulk reactions and bulk diffusion of NO_3 with a diffusion coefficient of $10^{-8} – 10^{-7}$ cm^2 s^{-1}. **N6**. Three dinitropyrenes (1,3-, 1,6- and 1,8-dinitropyrene) were also observed. **N7**. γ needed to be corrected to take non-diffusive transport of NO_3 to the particles into account, which was achieved by normalising γ to values obtained by (Gross and Bertram, 2008a) and (Mak et al., 2007). **N8**. Mixture of NO_3/N_2O_5 and NO_2. RH dependence of yields of the different nitro-PAH isomers interpreted in terms of switch from NO_3 as nitration agent at low RH to N_2O_5 (via ionisation to NO_2^+) at high RH. Similar change could be induced by changing the NO_3 to N_2O_5 ratio. At low RH the initial addition of radical to site with most electron density is followed by

NO_2 addition to the 2-position (ortho). At high RH the nitration occurs by electrophilic nitration by NO_2^+. **N9**. Changes in particle optical properties observed following nitration. **N10**. Other products were anthraquinone, 1,8-dihydroxyanthraquinone, 9-10-dinitroanthracene and anthrone. **N11**. Other products were hydroxynitrates of phenanthrene. **N12**. Also observed as secondary products: (dinitro-1-naphthyl)N-methylcarbamate, (hydroxy-1-naphthyl)N-methylcarbamate and (hydroxy-nitro-1-naphthyl)N-methylcarbamate. Relative loss rate of particulate carbaryl and isoprene (g) monitored to derive an effective rate constant for NO_3 + particulate carbaryl of 4.4×10^{-13} cm^3 $molecule^{-1}$ s^{-1}. **N13**. 2,6-dimethoxybenzoquinone produced following H-abstraction by NO_3 and addition of O_2 following by further elimination of CO_2 and addition of NO_3. **N14**. 4,6-dinitrogaiacol proposed to be formed via NO_3 addition followed by reactions and eliminations involving NO_2, HNO_3 and a further NO_3 radical and with 5-nitrovanillic acid as intermediate product. **N15**. Oxalic acid ($C_2H_2O_4$, ethanedioic acid), Vanillin (4-Hydroxy-3-methoxybenzaldehyde), 4,6.dinitrogaiacol, 5-nitrovanillin and vanillin observed. **N16**. Uptake coefficient increased (to ~ 30×10^{-3}) when the NO_3 concentration was reduced. γ was independent of exposure time, possibly due to volatilisation of surface products exposing fresh nitroguaiacol. **N17**. Also observed (in order of decreasing concentration): 3-nitro-fluoranthene, 8-nitro-fluoranthene, 7-nitro-fluoranthene and 1-nitro-fluoranthene. Authors suggest that these products are formed from N_2O_5 rather than NO_3 interactions as the main gas-phase product of NO_3 reaction (2-nitro-fluoranthene) was only very minor. This may have been a result of the large concentration of N_2O_5 and NO_2 relative to NO_3 used. Detection of 2-nitro-fluoranthene as dominant nitro-PAH in ambient particles led the authors to suggest that gas-phase processes were more important that particle reactions for its formation.

Table S2. Compilation of kinetic data on reactions of NO_3 with aliphatic VOCs and OVOCs in the aqueous phase taken from the CAPRAM kinetic database (Bräuer et al., 2016).

Compound	SMILES string	k_{298} (a)	$A^{(a)}$	$E_A/R^{(b)}$	Reference/Comment
Alkenes					
Isoprene	C=C(C)C=C	$1.0\cdot10^9$			(Raabe, 1996)
Monoalcohols					
Methanol	CO	$5.1\cdot10^5$	$9.4\cdot10^{11}$	4300	Average of (Exner et al., 1993 and {Rousse, 2004 #276)
Ethanol	CCO	$2.2\cdot10^6$	$1.4\cdot10^{11}$	3300	(Herrmann and Zellner, 1998)
Propanol	CCCO	$3.2\cdot10^6$			(Herrmann et al., 1994)
Butanol	CCCCO	$1.9\cdot10^6$			(Shastri and Huie, 1990)
Pentanol	CCCCCO	$2.4\cdot10^6$			(Shastri and Huie, 1990)
Hexanol	CCCCCCO	$3.3\cdot10^6$			(Shastri and Huie, 1990)
Heptanol	CCCCCCCO	$3.6\cdot10^6$			(Shastri and Huie, 1990)
Octanol	CCCCCCCCO	$5.8\cdot10^6$			(Shastri and Huie, 1990)
Iso-propanol	CC(O)C	$3.7\cdot10^6$	$3.1\cdot10^8$	1323	(Herrmann et al., 1994) with E_A/R of (Ito et al., 1989)
Iso-butanol	CC(C)CO	$1.6\cdot10^6$			(Shastri and Huie, 1990)
Tert-butanol	CC(C)CO	$6.6\cdot10^4$			(Herrmann et al., 1994)
Allyl alcohol	C=CCO	$2.2\cdot10^8$			Average of (Alfassi et al., 1993) and (Ito et al., 1989)
2-Butenol	CC=CCO	$2.1\cdot10^9$			(Alfassi et al., 1993)
3-Methyl 3-Buten-1-ol	C=C(C)CCO	$2.4\cdot10^9$			(Alfassi et al., 1993)
**Diols and polyols**					
Ethylene glycol	OCCO	$6.6\cdot10^6$	$7.1\cdot10^9$	2117	(Hoffmann et al., 2009)
1,2-Propanediol	CC(O)CO	$9.9\cdot10^6$	$6.8\cdot10^{10}$	2622	(Hoffmann et al., 2009)
Glycerol	OCC(O)CO	$1.3\cdot10^7$	$1.4\cdot10^{12}$	3452	(Hoffmann et al., 2009)
Aldehydes and gem-diols					
Formaldehyde	C=O	$3.5\cdot10^6$	$3.4\cdot10^6$	674	Average of (Ito et al., 1989)[1] and (Wayne et al., 1991) with E_A/R of (Wayne et al., 1991)
Hydrated formaldehyde	OCO	$1.0\cdot10^6$	$3.6\cdot10^{12}$	4500	(Exner et al., 1993)
Acetaldehyde	CC=O	$1.9\cdot10^6$			(Zellner et al., 1996)
Hydrated acetaldehyde	CC(O)O	$2.0\cdot10^6$			Average of (Zellner et al., 1996)[3] and (Rousse and George, 2004)
Propionaldehyde	CCC=O	$5.8\cdot10^7$	$3.2\cdot10^{11}$	2646	(de Semainville et al., 2007)
Butyraldehyde	CCCC=O	$5.6\cdot10^7$	$4.9\cdot10^{10}$	2045	(de Semainville et al., 2007)
Iso-butyraldehyde	CC(C)C=O	$6.3\cdot10^7$	$3.7\cdot10^8$	529	(Wayne et al., 1991)
2,2-Dimethyl propanal	CC(C)(C)C=O	$7.0\cdot10^7$	$3.8\cdot10^8$	505	(Wayne et al., 1991)
Methacrolein	C=C(C)C=O	$4.0\cdot10^7$	$5.8\cdot10^8$	842	(Schöne et al., 2014)
Methyl vinyl ketone		$9.7\cdot10^6$	$6.2\cdot10^8$	1203	(Schöne et al., 2014)
Glyoxal	O=CC=O	$4.5\cdot10^6$	$6.2\cdot10^{12}$	9897	(Schaefer et al., 2015)
Hydrated glyoxal	OC(O)C(O)O	$1.1\cdot10^6$	$8.9\cdot10^{10}$	3368	(Raabe, 1996)
Ketones					
Acetone	CC(=O)C	$3.7\cdot10^3$	$7.6\cdot10^9$	4330	(Herrmann and Zellner, 1998)
Hydroxy acetone	CC(=O)CO	$1.8\cdot10^7$	$4.0\cdot10^9$	1564	(de Semainville et al., 2007)

Compound	SMILES string	k_{298} [(a)]	$A^{(a)}$	$E_A/R^{(b)}$	Reference/Comment
Methyl ethyl ketone	CC(=O)CC	$9.7\cdot10^6$	$6.2\cdot10^8$	1200	(de Semainville et al., 2007)
Monocarboxylic acids					
Formic acid	C(=O)O	$3.8\cdot10^5$	$3.4\cdot10^{10}$	3400	(Exner et al., 1994)
Formate	C(=O)[O-]	$5.1\cdot10^7$	$8.2\cdot10^{10}$	2200	(Exner et al., 1994)
Acetic acid	CC(=O)O	$1.3\cdot10^4$	$4.9\cdot10^9$	3800	(Exner et al., 1994)
Acetate	CC(=O)[O-]	$2.9\cdot10^6$	$1.0\cdot10^{12}$	3800	(Exner et al., 1994)
Propionic acid	CCC(=O)O	$7.7\cdot10^4$			(Rousse and George, 2004)
Crotonic acid	CC=CC(=O)O	$5.1\cdot10^7$			(Neta and Huie, 1986)
Glycolic acid	OCC(=O)O	$9.1\cdot10^5$	$4.5\cdot10^{11}$	3969	(de Semainville et al., 2007)
Glycolate	OCC(=O)[O-]	$1.0\cdot10^7$	$1.8\cdot10^{11}$	3007	(de Semainville et al., 2007)
Lactic acid	CC(O)C(=O)O	$2.1\cdot10^6$	$1.0\cdot10^{11}$	3248	(de Semainville et al., 2007)
Lactate	CC(O)C(=O)[O-]	$1.0\cdot10^7$	$8.3\cdot10^{10}$	2646	(de Semainville et al., 2007)
Pyruvic acid	CC(=O)C(=O)O	$2.4\cdot10^6$	$8.8\cdot10^8$	1804	(de Semainville et al., 2007)
Pyruvate	CC(=O)C(=O)[O-]	$1.9\cdot10^7$	$3.7\cdot10^{11}$	2887	(de Semainville et al., 2007)
Acrylic acid	C=CC(=O)O	$6.9\cdot10^6$	$2.2\cdot10^{13}$	4450	(Schöne et al., 2014)
Acrylate	C=CC(=O)[O-]	$4.4\cdot10^7$	$2.2\cdot10^9$	1200	(Schöne et al., 2014)
Methacrylic acid	C=C(C)C(=O)O	$9.2\cdot10^7$			(Schöne et al., 2014)
Methacrylate	C=C(C)C(=O)[O-]	$1.7\cdot10^8$			(Schöne et al., 2014)
Dicarboxylic acids					
Oxalic acid	OC(=O)C(=O)O	$2.4\cdot10^4$			(Yang et al., 2004)
Oxalate monoanion	OC(=O)C(=O)[O-]	$6.1\cdot10^7$	$8.4\cdot10^9$	−2180	Average of (Yang et al., 2004) and (de Semainville et al., 2010) with E_A/R of (Raabe, 1996)
Oxalate dianion	[O-]C(=O)C(=O)[O-]	$2.2\cdot10^8$	$2.2\cdot10^{12}$	2766	Average of (Yang et al., 2004) and (de Semainville et al., 2010) with E_A/R of (de Semainville et al., 2010)
Malonic acid	OC(=O)CC(=O)O	$5.1\cdot10^4$			(de Semainville et al., 2010)
Malonate monoanion	OC(=O)CC(=O)[O-]	$5.6\cdot10^6$	$5.0\cdot10^{11}$	3368	(de Semainville et al., 2010)
Malonate dianion	[O-]C(=O)CC(=O)[O-]	$2.3\cdot10^7$	$6.3\cdot10^{11}$	3007	(de Semainville et al., 2010)
Succinic acid	OC(=O)CCC(=O)O	$5.0\cdot10^3$			(de Semainville et al., 2010)
Succinate monoanion	OC(=O)CCC(=O)[O-]	$1.1\cdot10^7$			(de Semainville et al., 2010)
Succinate dianion	[O-]C(=O)CCC(=O)[O-]	$1.8\cdot10^7$	$6.2\cdot10^{11}$	3127	(de Semainville et al., 2010)
Mesoxalic acid	OC(=O)C(=O)C(=O)O	$1.7\cdot10^6$	$5.1\cdot10^8$	1564	(de Semainville et al., 2010)
Mesoxalate monoanion	OC(=O)C(=O)C(=O)[O-]	$2.3\cdot10^7$			(de Semainville et al., 2010)
Mesoxalate dianion	[O-]C(=O)C(=O)C(=O)[O-]	$4.9\cdot10^7$	$1.4\cdot10^{12}$	3127	(de Semainville et al., 2010)
Fumaric acid	OC(=O)/C=C/C(=O)O	$<1.0\cdot10^6$			(Neta et al., 1988)
**Ethers and esters**					
Methyl tert-butyl ether	CC(C)(C)OC	$3.9\cdot10^5$			(Rousse and George, 2004)
Methyl formate	COC=O	$3.5\cdot10^6$			(Buxton et al., 2001)[6]
Ethyl formate	CCOC=O	$4.7\cdot10^6$			(Buxton et al., 2001)
Methyl acetate	CC(O)OC	$<10^4$			(Buxton et al., 2001)
Ethyl acetate	CC(=O)OCC	$<10^4$			(Buxton et al., 2001)
Dimethyl malonate	COC(=O)CC(=O)OC	$2.6\cdot10^4$			(Rousse and George, 2004)
Dimethyl succinate	COC(=O)CCC(=O)OC	$3.4\cdot10^4$			(Rousse and George, 2004)
Dimethyl carbonate	COC(=O)OC	$8.4\cdot10^4$			(Rousse and George, 2004)

Compound	SMILES string	k_{298} [(a)]	$A^{(a)}$	$E_A/R^{(b)}$	Reference/Comment
Diethyl carbonate	CCOC(=O)OCC	$1.5\cdot10^{4}$			(Rousse and George, 2004)
**Sugars**					
Erythritol	OCC(O)C(O)CO	$1.4\cdot10^{7}$	$3.4\cdot10^{10}$	2321	(Hoffmann et al., 2009)
Arabitol	OCC(O)C(O)C(O)CO	$1.5\cdot10^{7}$	$1.1\cdot10^{10}$	1997	(Hoffmann et al., 2009)
Mannitol	OCC(O)C(O)C(O)C(O)CO	$1.4\cdot10^{7}$	$5.1\cdot10^{10}$	2466	(Hoffmann et al., 2009)
Levoglucosan	OC1C(O)C(O)C2OC1OC2	$1.6\cdot10^{7}$	$2.3\cdot10^{10}$	2150	(Hoffmann et al., 2010)

[(a)] in M^{-1} s^{-1} ; [(b)] in K

[1]as given in (Wayne et al., 1991); [2]as given in (Toyota et al., 2004); [3]as given in CAPRAM database available at http://projects.tropos.de/capram/; [4]as given in NIST database (Ross et al., 1998); [5]as given in (de Semainville et al., 2007); [6] as given in (Herrmann, 2003)

Table S3. Correlation of OH and NO_3 rate expressions and statistical analysis data calculated from kinetic data of hydroxyl radical and nitrate radical of the reactions with organic compounds for the various compound classes. Kinetic data are based on the CAPRAM4.0 database (Bräuer et al., 2016) and recent data presented in the review of (Herrmann et al., 2015).

Compound class	A	B	R^2	N
Alcohols	1.283	-5.935	0.741	11
Di- and polyols	4.603	-35.44	0.859	10
Carbonyl compounds	1.097	-3.179	0.372	11
Monocarboxylic acids	1.151	-3.584	0.642	14
Dicarboxylic acids	0.701	0.848	0.161	13
Overall	0.599	1.247	0.205	59

Notes: Parameters of the regression equations $\log (k_{NO3} = M^{-1}s^{-1}) = A\cdot\log (k_{OH} = M^{-1}s^{-1}) + B$. N = Number of molecules in the regression.

Table S4. Summary of studies (offline methods) reporting the compounds originating from the reactions of BVOC and NO_3 in ambient samples

Location	Sample	Reported as	Technique	Reference
Europe				
Waldstein, Germany	$PM_{2.5}$	Monoterpene OS and NOS	HPLC/(-)ESI-TOFMS	(Iinuma et al., 2007)
K-Puszta, Hungary	$PM_{1.5}$	CHOS and CHONS compounds	Direct infusion (-)ESI-FTICR-MS	(Schmitt-Kopplin et al., 2010)
Silkeborg, Denmark	PM_1	Monoterpene OS and NOS	HPLC/(-)ESI-TOFMS	(Kristensen and Glasius, 2011)
Brasschaat, Belgium	$PM_{2.5}$	Monoterpene OS and NOS	HPLC/(-)ESI-Ion Trap MS	(Gómez-González et al., 2012)
Hamme, Belgium	PM_{10}	Monoterpene OS and NOS	HPLC/(-)ESI-Ion Trap MS	(Kahnt et al., 2013)
Hyytiälä, Finland	PM_1	Monoterpene OS and NOS	HPLC/(-)ESI-Orbitrap MS	(Kourtchev et al., 2013)
Greenland and Svalbard, Arctic region	PM_{10}	Monoterpene OS and NOS	HPLC/(-)ESI-TOFMS	(Hansen et al., 2014)
Copenhagen, Denmark	PM_1	Monoterpene OS and NOS	HPLC/(-)ESI-TOFMS	(Nguyen et al., 2014)
Cork, Ireland	PM_1	Monoterpene OS and NOS	HPLC/(-)ESI-Orbitrap MS	(Kourtchev et al., 2014)
North America				
Riverside, California, USA	$PM_{2.5}$	(Monoterpene) OS and NOS[a]	Direct infusion (-)ESI-FTICR-MS	(Reemtsma et al., 2006)
Southeastern USA	$PM_{2.5}$	Monoterpene OS and NOS	HPLC/(-)ESI-Ion Trap MS	(Gao et al., 2006)
Southeastern USA	$PM_{2.5}$	(Isoprene) and monoterpene OS and NOS[a]	UPLC/(-)ESI-TOFMS	(Surratt et al., 2008)
New Jersey, USA	Rainwater	Monoterpene OS and NOS[b]	Direct infusion (-)ESI-FTICR-MS	(Altieri et al., 2009a, b)
Fresno, California, USA	Fog	(Monoterpene) OS and NOS[b]	Direct infusion (-)ESI-FTICR-MS	(Mazzoleni et al., 2010)
Steamboat Springs,	PM	CHOS and CHONS	Direct infusion ESI-	(Mazzoleni et al.,

Colorado, USA		compounds	FTICR-MS	2012)
Steamboat Springs, Colorado, USA	Cloud water	CHOS and CHONS compounds	Direct infusion ESI-FTICR-MS	(Zhao et al., 2013)
Toronto, Canada	Rainwater	CHOS and CHONS compounds	Direct infusion ESI-FTICR-MS	(Cottrell et al., 2013)
Bakersfield, California, USA	PM_{10} (MOUDI) and $PM_{2.5}$	Monoterpene OS and NOS	Nano DESI-Orbitrap MS UPLC/(-)ESI-TOFMS	(O'Brien et al., 2014)
Los Angeles, USA	0.32-0.56 µm PM	CHOS compounds	Nano DESI-Orbitrap MS	(Tao et al., 2014)
North Atlantic Ocean	TSP (WSOC)[c]	CHOS and CHONS compounds	Direct infusion (-)ESI-FTICR-MS	(Wozniak et al., 2014)
North Atlantic Ocean	TSP (WISOC)[d]	CHOS and CHONS compounds	Direct infusion (-)ESI-FTICR-MS	(Willoughby et al., 2014)
North Carolina, USA	Rainwater	CHOS compounds	Direct infusion (-)ESI-FTICR-MS	(Mead et al., 2015)
Pico Mountain, Vermont, USA	Free tropospheric $PM_{2.5}$	CHOS compounds	Direct infusion (-)ESI-FTICR-MS	(Dzepina et al., 2015)
Asia				
Beijing, China	PM_{10} (Berner)	Monoterpene NOS	HPLC/(-)ESI-TOFMS	(van Pinxteren et al., 2009)
Pearl River Delta, China	$PM_{2.5}$	CHOS and CHONS compounds	Direct infusion (-)ESI-Orbitrap MS	(Lin et al., 2012)
Pearl River Delta, China	$PM_{2.5}$	CHOS and CHONS compounds	Direct infusion (-) or (+)ESI-Orbitrap MS	(Lin et al., 2012a)
Taiwan	$PM_{2.5}$	CHOS and CHONS compounds	Direct infusion (-)ESI-Orbitrap MS	(Lin et al., 2012b)
Pearl River Delta, China	$PM_{2.5}$	Isoprene and monoterpene NOS	HPLC/(-)ESI-QqQMS	(He et al., 2014)
Shanghai, China	0.4-0.7 µm PM	CHOS compounds	Nano DESI-Orbitrap MS	(Tao et al., 2014)
Three mega cities along Yangtze River, China	$PM_{2.5}$	Monoterpene OS and NOS	UHPLC/(-)ESI-Orbitrap MS	(Wang et al., 2015)
Oceania				

Melbourne, Australia	PM_{10}	Monoterpene NOS	HPLC/(-)ESI-TOFMS<br>UPLC/(-)ESI-TOFMS	(Iinuma et al., 2016)

[a]: isoprene OS and NOS were not attributed to the products originating from nitrate radical reactions

[b]: reported as chemical formulas that correspond to OS and NOS reported in Surratt et al., (2008)

[c]: Water Soluble Organic Carbon

[d]: Water Insoluble Organic Carbon

Table S5 Campaign average mass concentrations for submicrometer Organics (Org), $NO_{3,meas}$, $NO_{3,org}$, $NO_{3,inorg}$, Organic Nitrate Aerosols (ON), and ON/Org ratio. See references for measurement details.

Sample Site	Sample Period	Organics, Org ($\mu g\ m^{-3}$) [a]	$NO_{3,\ meas}$ ($\mu g\ m^{-3}$) [c]	$NO_{3,\ org}$ ($\mu g\ m^{-3}$)	$NO_{3,\ inorg}$ ($\mu g\ m^{-3}$) [d]	Organic Nitrate Aerosols, ON ($\mu g\ m^{-3}$)	ON/Org ratio	Reference/Notes
Bakersfield, CA, USA	15 May - 29 June, 2010	3.38	0.58	0.16	0.42	0.79	0.23	• Rollins et al. (2012; 2013)<br>• $NO_{3,org}$ measured by TD-LIF<br>• Assume MW of organic nitrate = 300 g/mol when converting $NO_{3,org}$ to ON<br>• ON corresponds to the sum of the mass of both the org portion and nitrate portion of organic nitrates<br>• ON/OA = 0.17 when $NO_{3,org}$ measured by FTIR is used
San Diego, CA, USA	23 February - 30 March, 2009	1.60[b]	0.69	0.05	0.64	0.17	0.11	• Day et al. (2010) Table 2<br>• $NO_{3,org}$ measured by FTIR and the value shown in this table is calculated by scaling by 62/68 to obtain $-ONO_2$ mass since FTIR is reported as 0.5C + $-ONO_2$<br>• Assume MW of organic nitrate = 230 g/mol when converting $NO_{3,org}$ to ON<br>• ON corresponds to the sum of the mass of both the org portion and nitrate portion of organic nitrates<br>• ON/OA ratio is calculated by scaling the organic nitrate functional group to OM measured by FTIR, 3.2% x (230/68) = 11%

Woodland Park, CO, USA	July - August, 2011	1.86	0.07	0.06	0.01	0.16	0.09	• Fry et al. (2013)<br>• Use NO_2^+/NO^+ method (R_{ON} = 0.13) to obtain $NO_{3,org}$<br>• Assume MW of organic nitrate = 230 g/mol when converting $NO_{3,org}$ to ON<br>• ON corresponds to the mass of the org portion of organic nitrates
Atlanta (Jefferson Street), GA, USA	10 May - 2 June, 2012	9.10	0.36	0.27	0.09	1.31	0.14	• Xu et al. (2015b) Tables 1 and 2<br>• Combine PMF method and NO^+/NO_2^+ method (R_{ON} = 5 and 10) to obtain upper bound for $NO_{3,org}$; thus the $NO_{3,inorg}$ values in the table are lower bound values<br>• Assume MW of organic nitrate = 300 g/mol when converting $NO_{3,org}$ to ON<br>• ON corresponds to the sum of the mass of both the org portion and nitrate portion of organic nitrates<br>• Data also available from Georgia Tech site and Roadside site, see Xu et al. (2015a; 2015b) for details
	6 November - 4 December, 2012	7.90	1.18	0.25	0.93	1.19	0.15	
Yorkville, GA, USA	26 June - 20 July, 2012	11.20	0.28	0.28	0.00	1.35	0.12	
	5 December - 20 January, 2013	3.20	0.76	0.16	0.60	0.77	0.25	
Centreville, AL, USA	24 June - 15 July, 2013	3.63	0.08	0.08	0.00[e, f]	0.39	0.10	
Barcelona, Spain	March, 2009	8.10	3.65	0.48	3.17	1.07	0.13	• Kiendler-Scharr et al. (2016) Table S2<br>• Use NO_2^+/NO^+ method (R_{ON} = 0.1) to obtain $NO_{3,org}$<br>• Assume MW of organic nitrate = 200 g/mol when converting $NO_{3,org}$ to ON<br>• ON corresponds to the mass of the org portion
Cabauw, Netherlands	March, 2009	1.26	2.51	0.38	2.13	0.84	0.67	
Finokalia, Greece	March, 2009	1.41	0.05	0.03	0.02	0.07	0.05	

of organic nitrates
• Data also available for May, 2008 for selected sites, see Kiendler-Scharr et al. (2016) for details

Jungfraujoch, Switzerland	May, 2008	0.70	0.31	0.03	0.28	0.06	0.08
Melpitz, Germany	March, 2009	2.10	3.68	0.69	2.99	1.54	0.73
	October, 2008	3.88	2.98	0.74	2.24	1.65	0.43
Payerne, Switzerland	March, 2009	4.05	3.89	0.71	3.19	1.57	0.39
	October, 2008	5.36	2.72	0.20	2.52	0.44	0.08
Puijo, Finland	October, 2008	1.19	0.10	0.07	0.02	0.16	0.14
San Pietro Capofiume, Italy	April, 2008	3.83	2.93	1.32	1.61	2.94	0.77
Vavihill, Switzerland	March, 2009	3.72	3.17	0.98	2.19	2.17	0.58
	October, 2008	2.46	1.66	0.75	0.92	1.66	0.67
Harwell, UK	October, 2008	3.19	3.15	0.90	2.25	2.00	0.63
Helsinki, Finland	March, 2009	2.92	0.94	0.14	0.79	0.32	0.11
Hyytiälä, Finland	March, 2009	1.36	0.23	0.09	0.14	0.20	0.15
	October, 2008	0.82	0.11	0.09	0.02	0.19	0.23
Kpuszta, Hungary	October, 2008	5.27	1.97	0.43	1.54	0.96	0.18
Mace Head, Ireland	March, 2009	1.06	0.58	0.22	0.36	0.49	0.47
Montseny, Spain	March, 2009	3.27	3.25	0.98	2.27	2.18	0.67
Puy de Dome, France	March, 2009	0.58	0.74	0.16	0.57	0.36	0.63
	October, 2008	1.77	0.82	0.23	0.58	0.52	0.29

[a]Organics measured by aerosol mass spectrometer (AMS), except for San Diego data

[b]This value is OM measured by FTIR

[c]Nitrates (total nitrates) measured by aerosol mass spectrometer (AMS), corresponds to the mass of $-ONO_2$ functionality

[d]Calcuated by ($NO_{3,meas}$ - $NO_{3,org}$)

[e]Submicrometer $NO_{3,inorg}$ was also measured by PILS to be 0.04 μg m^{-3}, see Xu et al. (2015b) for details

[f]$NO_{3,inorg}$ in $PM_{2.5}$ was measured by MARGA to be 0.37 μg m^{-3} (stdev 0.2 μg m^{-3}), see Allen et al. (2015) for details

References

Alfassi, Z. B., Padmaja, S., Neta, P., and Huie, R. E.: Rate Constants for Reactions of NO_3. Radicals with Organic-Compounds in Water and Acetonitrile, J Phys Chem-Us, 97, 3780-3782, 1993.

Allen, H. M., Draper, D. C., Ayres, B. R., Ault, A., Bondy, A., Takahama, S., Modini, R. L., Baumann, K., Edgerton, E., Knote, C., Laskin, A., Wang, B., and Fry, J. L.: Influence of crustal dust and sea spray supermicron particle concentrations and acidity on inorganic NO3− aerosol during the 2013 Southern Oxidant and Aerosol Study, Atmos. Chem. Phys., 15, 10669-10685, 10.5194/acp-15-10669-2015, 2015.

Altieri, K. E., Turpin, B. J., and Seitzinger, S. P.: Oligomers, organosulfates, and nitrooxy organosulfates in rainwater identified by ultra-high resolution electrospray ionization FT-ICR mass spectrometry, Atmos Chem Phys, 9, 2533-2542, 2009a.

Altieri, K. E., Turpin, B. J., and Seitzinger, S. P.: Composition of Dissolved Organic Nitrogen in Continental Precipitation Investigated by Ultra-High Resolution FT-ICR Mass Spectrometry, Environmental Science & Technology, 43, 6950-6955, 10.1021/es9007849, 2009b.

Atkinson, R., Aschmann, S. M., Winer, A. M., and Pitts, J. N.: Kinetics of the Gas-Phase Reactions of NO_3 Radicals with a Series of Dialkenes, Cycloalkenes, and Monoterpenes at 295 +/- 1-K, Environmental Science & Technology, 18, 370-375, 1984.

Atkinson, R., Aschmann, S. M., Winer, A. M., and Pitts, J. N.: Kinetics and atmospheric implications of the gas-phase reactions of NO_3 radicals with a series of monoterpenes and related organics at 294 +/- 2-K, Environmental Science & Technology, 19, 159-163, 10.1021/es00132a009, 1985.

Atkinson, R., Aschmann, S. M., and Pitts, J. N.: Rate constants for the gas-phase reactions of the nitrate radical with a series of organic compounds at 296 ± 2 K, J Phys Chem A, 92, 3454-3457, 1988.

Atkinson, R., Aschmann, S. M., and Arey, J.: Rate constants for the gas-phase reactions of OH and NO_3 radicals and O_3 with sabinene and camphene at 296 ± 2 K, Atmospheric Environment, 24, 2647-2654, 1990.

Atkinson, R., Arey, J., Aschmann, S. M., Corchnoy, S. B., and Shu, Y.: Rate constants for the gas-phase reactions of *cis*-3-Hexen-1-ol, *cis*-3-Hexenylacetate, *trans*-2-Hexenal, and Linalool with OH and NO_3 radicals and O_3 at 296 ± 2 K, and OH radical formation yields from the O_3 reactions, Int J Chem Kinet, 27, 941-955, 1995.

Baker, J., Arey, J., and Atkinson, R.: Kinetics of the gas-phase reactions of OH radicals, NO3 radicals and O3 with three C7 carbonyls formed from the atmospheric reactions of myrcene, ocimene and terpinolene, J. Atmos. Chem., 48, 241-260, 2004.

Barnes, I., Bastian, V., Becker, K. H., and Tong, Z.: Kinetics and Products of the Reactions of NO_3 with Monoalkenes, Dialkenes, and Monoterpenes, J Phys Chem-Us, 94, 2413-2419, 1990.

Benter, T., and Schindler, R. N.: Absolute rate coefficients for the reaction of NO_3 radicals with simple dienes, Chemical Physics Letters, 145, 67-70, 10.1016/0009-2614(88)85134-0, 1988.

Berndt, T., and Boge, O.: Gas-phase reaction of NO_3 radicals with isoprene: A kinetic and mechanistic study, Int J Chem Kinet, 29, 755-765, 1997.

Bräuer, P., Mouchel-Vallon, C., Tilgner, A., Mutzel, A., Böge, O., Rodigast, M., Poulain, L., van Pinxteren, D., Aumont, B., and Herrmann, H.: Development of a protocol designed for the self-generation of explicit aqueous phase oxidation schemes of organic compounds, Atmos. Chem. Phys. Discuss., in preparation, 2016.

Buxton, G. V., Wang, J. Q., and Salmon, G. A.: Rate constants for the reactions of NO_3 center dot, SO_4 center dot- and Cl-center dot radicals with formate and acetate esters in aqueous solution, Physical Chemistry Chemical Physics, 3, 2618-2621, 2001.
Cottrell, B. A., Gonsior, M., Isabelle, L. M., Luo, W., Perraud, V., McIntire, T. M., Pankow, J. F., Schmitt-Kopplin, P., Cooper, W. J., and Simpson, A. J.: A regional study of the seasonal variation in the molecular composition of rainwater, Atmospheric Environment, 77, 588-597, 10.1016/j.atmosenv.2013.05.027, 2013.
Day, D. A., Liu, S., Russell, L. M., and Ziemann, P. J.: Organonitrate group concentrations in submicron particles with high nitrate and organic fractions in coastal southern California, Atmospheric Environment, 44, 1970-1979, 2010.
de Semainville, P. G., Hoffmann, D., George, C., and Herrmann, H.: Study of nitrate radical (NO3) reactions with carbonyls and acids in aqueous solution as a function of temperature, Physical Chemistry Chemical Physics, 9, 958-968, 2007.
de Semainville, P. G., Anna, B. D., and George, C.: Aqueous Phase Reactivity of Nitrate Radicals (NO3) Toward Dicarboxylic Acids, Z Phys Chem, 224, 1247-1260, 2010.
Dzepina, K., Mazzoleni, C., Fialho, P., China, S., Zhang, B., Owen, R. C., Helmig, D., Hueber, J., Kumar, S., Perlinger, J. A., Kramer, L. J., Dziobak, M. P., Ampadu, M. T., Olsen, S., Wuebbles, D. J., and Mazzoleni, L. R.: Molecular characterization of free tropospheric aerosol collected at the Pico Mountain Observatory: a case study with a long-range transported biomass burning plume, Atmos Chem Phys, 15, 5047-5068, 10.5194/acp-15-5047-2015, 2015.
Exner, M., Herrmann, H., Michel, J. W., and Zellner, R.: Laser pulse initiated measurements of NO_3 reactions with S(IV) and organic compounds in aqueous solutions, in: Proceedings of EUROTRAC Symposium ´92: Photo-oxi dants: Precursors and Products, edited by: Borrell, P. M., Borrell, P., Cvitaš, T., and Seiler, W., SPB Academic Publishing, The Hague, NL, 615 – 618, 1993.
Exner, M., Herrmann, H., and Zellner, R.: Rate Constants for the Reactions of the NO_3 Radical with $HCOOH/HCOO^-$ and CH_3COOH/CH_3COO^- in Aqueous-Solution between 278-K and 328-K, Journal of Atmospheric Chemistry, 18, 359-378, 1994.
Fry, J. L., Kiendler-Scharr, A., Rollins, A. W., Brauers, T., Brown, S. S., Dorn, H. P., Dubé, W. P., Fuchs, H., Mensah, A., Rohrer, F., Tillmann, R., Wahner, A., Wooldridge, P. J., and Cohen, R. C.: SOA from limonene: role of NO3 in its generation and degradation, Atmos. Chem. Phys., 11, 3879-3894, 2011.
Fry, J. L., Draper, D. C., Zarzana, K. J., Campuzano-Jost, P., Day, D. A., Jimenez, J. L., Brown, S. S., Cohen, R. C., Kaser, L., Hansel, A., Cappellin, L., Karl, T., Hodzic Roux, A., Turnipseed, A., Cantrell, C., Lefer, B., and Grossberg, N.: Observations of gas- and aerosol-phase organic nitrates at BEACHON-RoMBAS 2011, Atmos. Chem. Phys., 13, 8585-8605, 2013.
Gao, S., Surratt, J. D., Knipping, E. M., Edgerton, E. S., Shahgholi, M., and Seinfeld, J. H.: Characterization of polar organic components in fine aerosols in the southeastern United States: Identity, origin, and evolution, Journal of Geophysical Research: Atmospheres, 111, n/a-n/a, 10.1029/2005JD006601, 2006.
Gómez-González, Y., Wang, W., Vermeylen, R., Chi, X., Neirynck, J., Janssens, I. A., Maenhaut, W., and Claeys, M.: Chemical characterisation of atmospheric aerosols during a 2007 summer field campaign at Brasschaat, Belgium: sources and source processes of biogenic secondary organic aerosol, Atmos Chem Phys, 12, 125-138, 10.5194/Acp-12-125-2012, 2012.
Gross, S., and Bertram, A. K.: Reactive uptake of NO_3, N_2O_5, NO_2, HNO_3, and O_3 on three types of polycyclic aromatic hydrocarbon surfaces, J. Phys. Chem. A, 112, 3104-3113, 2008a.

Gross, S., and Bertram, A. K.: Reactive Uptake of NO_3, N_2O_5, NO_2, HNO_3, and O_3 on Three Types of Polycyclic Aromatic Hydrocarbon Surfaces, J. Phys. Chem. A, 2008b.
Gross, S., and Bertram, A. K.: Products and kinetics of the reaction of an alkane monolayer and a terminal alkene monolayer with NO_3 radicals, J. Geophys. Res., 114, D02307, doi:10.1029/2008JD010987, 2009.
Gross, S., Iannone, R., Xiao, S., and Bertram, A. K.: Reactive uptake studies of NO_3 and N_2O_5 on alkenoic acid, alkanoate, and polyalcohol substrates to probe nighttime aerosol chemistry, Physical Chemistry Chemical Physics, 11, 7792-7803, 2009a.
Gross, S., Iannone, R. Q., Xiao, S., and Bertram, A. K.: Reactive uptake studies of NO_3 and N_2O_5 on alkenoic acid, alkanoate and polyalcohol substrates to probe nighttime aerosol chemistry, Phys. Chem. Chem. Phys., 11, 7792-7803, 2009b.
Hansen, A. M. K., Kristensen, K., Nguyen, Q. T., Zare, A., Cozzi, F., Nojgaard, J. K., Skov, H., Brandt, J., Christensen, J. H., Strom, J., Tunved, P., Krejci, R., and Glasius, M.: Organosulfates and organic acids in Arctic aerosols: speciation, annual variation and concentration levels, Atmos Chem Phys, 14, 7807-7823, 10.5194/acp-14-7807-2014, 2014.
Hanson, D. R., Ravishankara, A. R., and Solomon, S.: Heterogeneous reactions in sulfuric acid aerosols: A framework for model calculations, J. Geophys. Res., 99, 3615-3629, 1994.
He, Q.-F., Ding, X., Wang, X.-M., Yu, J.-Z., Fu, X.-X., Liu, T.-Y., Zhang, Z., Xue, J., Chen, D.-H., Zhong, L.-J., and Donahue, N. M.: Organosulfates from Pinene and Isoprene over the Pearl River Delta, South China: Seasonal Variation and Implication in Formation Mechanisms, Environmental Science & Technology, 10.1021/es501299v, 2014.
Herrmann, H., Exner, M., and Zellner, R.: Reactivity Trends in Reactions of the Nitrate Radical (No3) with Inorganic and Organic Cloudwater Constituents, Geochim Cosmochim Ac, 58, 3239-3244, 1994.
Herrmann, H., and Zellner, R.: Reactions of NO_3-Radicals in Aqueous Solution, in: N-Centered Radicals, edited by: Alfassi, Z. B., Wiley, Chichester, 291-343, 1998.
Herrmann, H.: Kinetics of aqueous phase reactions relevant for atmospheric chemistry, Chemical Reviews, 103, 4691-4716, 10.1021/cr020658q, 2003.
Herrmann, H., Schaefer, T., Tilgner, A., Styler, S. A., Weller, C., Teich, M., and Otto, T.: Tropospheric Aqueous-Phase Chemistry: Kinetics, Mechanisms, and Its Coupling to a Changing Gas Phase, Chemical Reviews, 115, 4259-4334, 10.1021/cr500447k, 2015.
Hoffmann, D., Weigert, B., Barzaghi, P., and Herrmann, H.: Reactivity of poly-alcohols towards OH, NO_3 and SO_4^- in aqueous solution, Physical Chemistry Chemical Physics, 11, 9351-9363, 2009.
Hoffmann, D., Tilgner, A., Iinuma, Y., and Herrmann, H.: Atmospheric Stability of Levoglucosan: A Detailed Laboratory and Modeling Study, Environmental Science & Technology, 44, 694-699, 10.1021/es902476f, 2010.
Hung, H., -M,, Katrib, Y., and Martin, S., T.: Products and Mechanisms of the Reaction of Oleic Acid with Ozone and Nitrate Radical, The Journal of Physical Chemistry A, 109, 4517-4530, 10.1021/jp0500900, 2005.
Iannone, R., Xiao, S., and Bertram, A. K.: Potentially important nighttime heterogeneous chemistry: NO_3 with aldehydes and N_2O_5 with alcohols, Physical Chemistry Chemical Physics, 13, 10214-10223, 10.1039/C1CP20294D, 2011.
Iinuma, Y., Müller, C., Berndt, T., Böge, O., Claeys, M., and Herrmann, H.: Evidence for the Existence of Organosulfates from b-Pinene Ozonolysis in Ambient Secondary Organic Aerosol, Environ. Sci. Technol., 41, 6678-6683, http://dx.doi.org/10.1021/es070938t, 2007.

Iinuma, Y., Keywood, M., and Herrmann, H.: Characterisation of primary and secondary organic aerosols in Melbourne airshed: The influence of biogenic emissions, wood smoke and bushfires, Atmospheric Environment, 54-63, 10.1016/j.atmosenv.2015.12.014, 2016.

Ito, O., Akiho, S., and Iino, M.: Kinetics for Reactions of the Nitrate Radical (NO_3) with Aldehydes in Acetonitrile, J Phys Chem-Us, 93, 4079-4083, 1989.

Kahnt, A., Behrouzi, S., Vermeylen, R., Shalamzari, M. S., Vercauteren, J., Roekens, E., Claeys, M., and Maenhaut, W.: One-year study of nitro-organic compounds and their relation to wood burning in PM10 aerosol from a rural site in Belgium, Atmospheric Environment, 81, 561-568, 10.1016/J.Atmosenv.2013.09.041, 2013.

Kaiser, J. C., Riemer, N., and Knopf, D. A.: Detailed heterogeneous oxidation of soot surfaces in a particle-resolved aerosol model, Atmos. Chem. Phys., 11, 4505-4520, 10.5194/acp-11-4505-2011, 2011.

Kiendler-Scharr, A., Mensah, A. A., Friese, E., Topping, D., Nemitz, E., Prevot, A. S. H., Äijälä, M., Allan, J., Canonaco, F., Canagaratna, M., Carbone, S., Crippa, M., Dall Osto, M., Day, D. A., De Carlo, P., Di Marco, C. F., Elbern, H., Eriksson, A., Freney, E., Hao, L., Herrmann, H., Hildebrandt, L., Hillamo, R., Jimenez, J. L., Laaksonen, A., McFiggans, G., Mohr, C., O'Dowd, C., Otjes, R., Ovadnevaite, J., Pandis, S. N., Poulain, L., Schlag, P., Sellegri, K., Swietlicki, E., Tiitta, P., Vermeulen, A., Wahner, A., Worsnop, D., and Wu, H. C.: Organic nitrates from night-time chemistry are ubiquitous in the European submicron aerosol, Geophys Res Lett, n/a-n/a, 10.1002/2016GL069239, 2016.

Knopf, D. A., Mak, J., Gross, S., and Bertram, A. K.: Does atmospheric processing of saturated hydrocarbon surfaces by NO_3 lead to volatilization, Geophys. Res. Lett., 33, L17816, doi:10.1029/2006GL026884, 2006.

Knopf, D. A., Forrester, S. M., and Slade, J. H.: Heterogeneous oxidation kinetics of organic biomass burning aerosol surrogates by O_3, NO_2, N_2O_5, and NO_3, Physical Chemistry Chemical Physics, 13, 21050-21062, 2011.

Kourtchev, I., Fuller, S., Aalto, J., Ruuskanen, T. M., McLeod, M. W., Maenhaut, W., Jones, R., Kulmala, M., and Kalberer, M.: Molecular Composition of Boreal Forest Aerosol from Hyytiala, Finland, Using Ultrahigh Resolution Mass Spectrometry, Environmental Science & Technology, 47, 4069-4079, 10.1021/es3051636, 2013.

Kourtchev, I., O'Connor, I. P., Giorio, C., Fuller, S. J., Kristensen, K., Maenhaut, W., Wenger, J. C., Sodeau, J. R., Glasius, M., and Kalberer, M.: Effects of anthropogenic emissions on the molecular composition of urban organic aerosols: An ultrahigh resolution mass spectrometry study, Atmospheric Environment, 89, 525-532, 10.1016/J.Atmosenv.2014.02.051, 2014.

Kristensen, K., and Glasius, M.: Organosulfates and oxidation products from biogenic hydrocarbons in fine aerosols from a forest in North West Europe during spring, Atmospheric Environment, 45, 4546-4556, 10.1016/j.atmosenv.2011.05.063, 2011.

Lee, L., Wooldridge, P., Nah, T., Wilson, K., and Cohen, R.: Observation of rates and products in the reaction of NO_3 with submicron squalane and squalene aerosol, Physical Chemistry Chemical Physics, 15, 882-892, 10.1039/C2CP42500A, 2013.

Lin, P., Rincon, A. G., Kalberer, M., and Yu, J. Z.: Elemental Composition of HULIS in the Pearl River Delta Region, China: Results Inferred from Positive and Negative Electrospray High Resolution Mass Spectrometric Data, Environmental Science & Technology, 46, 7454-7462, 10.1021/es300285d, 2012.

Lu, J. W., Flores, J. M., Lavi, A., Abo-Riziq, A., and Rudich, Y.: Changes in the optical properties of benzo[a]pyrene-coated aerosols upon heterogeneous reactions with NO_2

and NO_3, Physical Chemistry Chemical Physics, 13, 6484-6492, 10.1039/c0cp02114h, 2011.
Mak, J., Gross, S., and Bertram, A. K.: Uptake of NO_3 on soot and pyrene surfaces, Geophys. Res. Lett., 34, L10804
10.1029/2006gl029756, 2007.
Mazzoleni, L. R., Ehrmann, B. M., Shen, X. H., Marshall, A. G., and Collett, J. L.: Water-Soluble Atmospheric Organic Matter in Fog: Exact Masses and Chemical Formula Identification by Ultrahigh-Resolution Fourier Transform Ion Cyclotron Resonance Mass Spectrometry, Environmental Science & Technology, 44, 3690-3697, 10.1021/Es903409k, 2010.
Mazzoleni, L. R., Saranjampour, P., Dalbec, M. M., Samburova, V., Hallar, A. G., Zielinska, B., Lowenthal, D. H., and Kohl, S.: Identification of water-soluble organic carbon in non-urban aerosols using ultrahigh-resolution FT-ICR mass spectrometry: organic anions, Environmental Chemistry, 9, 285-297, 10.1071/En11167, 2012.
Mead, R. N., Felix, J. D., Avery, G. B., Kieber, R. J., Willey, J. D., and Podgorski, D. C.: Characterization of CHOS compounds in rainwater from continental and coastal storms by ultrahigh resolution mass spectrometry, Atmospheric Environment, 105, 162-168, 10.1016/j.atmosenv.2015.01.057, 2015.
Moise, T., Talukdar, R. K., Frost, G. J., Fox, R. W., and Rudich, Y.: Reactive uptake of NO_3 by liquid and frozen organics, J. Geophys. Res., 107, D24014, DOI:24010.21029/22001JD000334, 2002.
Neta, P., and Huie, R. E.: Rate Constants for Reactions of NO_3 Radicals in Aqueous-Solutions, J Phys Chem-Us, 90, 4644-4648, 10.1021/J100410a035, 1986.
Neta, P., Huie, R. E., and Ross, A. B.: Rate Constants for Reactions of Inorganic Radicals in Aqueous Solution, Journal of Physical and Chemical Reference Data, 17, 1027-1284, doi:http://dx.doi.org/10.1063/1.555808, 1988.
Nguyen, Q. T., Christensen, M. K., Cozzi, F., Zare, A., Hansen, A. M. K., Kristensen, K., Tulinius, T. E., Madsen, H. H., Christensen, J. H., Brandt, J., Massling, A., Nojgaard, J. K., and Glasius, M.: Understanding the anthropogenic influence on formation of biogenic secondary organic aerosols in Denmark via analysis of organosulfates and related oxidation products, Atmos Chem Phys, 14, 8961-8981, 10.5194/acp-14-8961-2014, 2014.
O'Brien, R. E., Laskin, A., Laskin, J., Rubitschun, C. L., Surratt, J. D., and Goldstein, A. H.: Molecular characterization of S- and N-containing organic constituents in ambient aerosols by negative ion mode high-resolution Nanospray Desorption Electrospray Ionization Mass Spectrometry: CalNex 2010 field study, Journal of Geophysical Research: Atmospheres, 119, 12,706-712,720, 10.1002/2014JD021955, 2014.
Pfrang, C., Shiraiwa, M., and Pöschl, U.: Coupling aerosol surface and bulk chemistry with a kinetic double layer model (K2-SUB): oxidation of oleic acid by ozone, Atmos. Chem. Phys., 10, 4537-4557, 10.5194/acp-10-4537-2010, 2010.
Pitts, J. N., Jr., Bierman, H. W., Atkinson, R., and Winer, A. M.: Atmospheric implications of simultaneous measurements of NO3 radicals and HONO, Geophys. Res. Lett., 11, 557-560, 1984.
Pöschl, U., Rudich, Y., and Ammann, M.: Kinetic model framework for aerosol and cloud surface chemistry and gas-particle interactions – Part 1: General equations, parameters, and terminology, Atmos. Chem. Phys., 7, 5989-6023, 10.5194/acp-7-5989-2007, 2007.
Raabe, G.: Eine laserphotolytische Studie zur Kinetik von Reaktionen des NO_3-Radikals in wa□ssriger Lo□sung, University of Essen, 1996.

Reemtsma, T., These, A., Venkatachari, P., Xia, X., Hopke, P. K., Springer, A., and Linscheid, M.: Identification of Fulvic Acids and Sulfated and Nitrated Analogues in Atmospheric Aerosol by Electrospray Ionization Fourier Transform Ion Cyclotron Resonance Mass Spectrometry, Analytical Chemistry, 78, 8299-8304, 10.1021/ac061320p, 2006.

Renbaum-Wolff, L., Grayson, J. W., Bateman, A. P., Kuwata, M., Sellier, M., Murray, B. J., Shilling, J. E., Martin, S. T., and Bertram, A. K.: Viscosity of α-pinene secondary organic material and implications for particle growth and reactivity, Proceedings of the National Academy of Sciences, 110, 8014-8019, 2013.

Robinson, A. L., Donahue, N. M., and Rogge, W. F.: Photochemical oxidation and changes in molecular composition of organic aerosol in the regional context, Journal of Geophysical Research: Atmospheres, 111, n/a-n/a, 10.1029/2005JD006265, 2006.

Roldin, P., Eriksson, A. C., Nordin, E. Z., Hermansson, E., Mogensen, D., Rusanen, A., Boy, M., Swietlicki, E., Svenningsson, B., Zelenyuk, A., and Pagels, J.: Modelling non-equilibrium secondary organic aerosol formation and evaporation with the aerosol dynamics, gas- and particle-phase chemistry kinetic multilayer model ADCHAM, Atmos. Chem. Phys., 14, 7953-7993, 10.5194/acp-14-7953-2014, 2014.

Rollins, A. W., Browne, E. C., Min, K. E., Pusede, S. E., Wooldridge, P. J., Gentner, D. R., Goldstein, A. H., Liu, S., Day, D. A., Russell, L. M., and Cohen, R. C.: Evidence for NO_x Control over Nighttime SOA Formation, Science, 337, 1210-1212, 2012.

Rollins, A. W., Pusede, S., Wooldridge, P., Min, K. E., Gentner, D. R., Goldstein, A. H., Liu, S., Day, D. A., Russell, L. M., Rubitschun, C. L., Surratt, J. D., and Cohen, R. C.: Gas/particle partitioning of total alkyl nitrates observed with TD-LIF in Bakersfield, Journal of Geophysical Research: Atmospheres, 118, 6651-6662, 10.1002/jgrd.50522, 2013.

Ross, A. B., Bielski, B. H. J., Buxton, G. V., Cabelli, D. E., Helman, W. P., Huie, R. E., Grodkovski, J., Neta, P., Mulazzani, Q. G., and Wilkinson, F.: NIST standard reference database 40: NDRL/NIST solution kinetics database vers. 3.0, 1998.

Rousse, D., and George, C.: A novel long path photolysis cell-application to the reactivity of selected organic compounds toward the nitrate radical (NO_3), Physical Chemistry Chemical Physics, 6, 3408-3414, 2004.

Schaefer, T., van Pinxteren, D., and Herrmann, H.: Multiphase Chemistry of Glyoxal: Revised Kinetics of the Alkyl Radical Reaction with Molecular Oxygen and the Reaction of Glyoxal with OH, NO_3, and SO_4^- in Aqueous Solution, Environmental Science & Technology, 49, 343-350, 10.1021/es505860s, 2015.

Schmitt-Kopplin, P., Gelencser, A., Dabek-Zlotorzynska, E., Kiss, G., Hertkorn, N., Harir, M., Hong, Y., and Gebefugi, I.: Analysis of the Unresolved Organic Fraction in Atmospheric Aerosols with Ultrahigh-Resolution Mass Spectrometry and Nuclear Magnetic Resonance Spectroscopy: Organosulfates As Photochemical Smog Constituents, Analytical Chemistry, 82, 8017-8026, 10.1021/ac101444r, 2010.

Schöne, L., Schindelka, J., Szeremeta, E., Schaefer, T., Hoffmann, D., Rudzinski, K. J., Szmigielski, R., and Herrmann, H.: Atmospheric aqueous phase radical chemistry of the isoprene oxidation products methacrolein, methyl vinyl ketone, methacrylic acid and acrylic acid - kinetics and product studies, Physical Chemistry Chemical Physics, 16, 6257-6272, 10.1039/C3CP54859G, 2014.

Shastri, L. V., and Huie, R. E.: Rate Constants for Hydrogen Abstraction Reactions of NO_3 in Aqueous-Solution, Int J Chem Kinet, 22, 505-512, 1990.

Shiraiwa, M., Garland, R. M., and Pöschl, U.: Kinetic double-layer model of aerosol surface chemistry and gas-particle interactions (K2-SURF): Degradation of polycyclic

aromatic hydrocarbons exposed to O3, NO2, H2O, OH and NO3, Atmos. Chem. Phys., 9, 9571-9586, 2009.
Shiraiwa, M., Pfrang, C., and Pöschl, U.: Kinetic multi-layer model of aerosol surface and bulk chemistry (KM-SUB): the influence of interfacial transport and bulk diffusion on the oxidation of oleic acid by ozone, Atmos. Chem. Phys., 10, 3673-3691, 10.5194/acp-10-3673-2010, 2010.
Shiraiwa, M., Pöschl, U., and Knopf, D. A.: Multiphase Chemical Kinetics of NO_3 Radicals Reacting with Organic Aerosol Components from Biomass Burning, Environmental Science & Technology, 46, 6630-6636, 10.1021/es300677a, 2012.
Surratt, J. D., Gómez-González, Y., Chan, A. W. H., Vermeylen, R., Shahgholi, M., Kleindienst, T. E., Edney, E. O., Offenberg, J. H., Lewandowski, M., Jaoui, M., Maenhaut, W., Claeys, M., Flagan, R. C., and Seinfeld, J. H.: Organosulfate Formation in Biogenic Secondary Organic Aerosol, The Journal of Physical Chemistry A, 112, 8345-8378, 10.1021/jp802310p, 2008.
Tao, S., Lu, X., Levac, N., Bateman, A. P., Nguyen, T. B., Bones, D. L., Nizkorodov, S. A., Laskin, J., Laskin, A., and Yang, X.: Molecular Characterization of Organosulfates in Organic Aerosols from Shanghai and Los Angeles Urban Areas by Nanospray-Desorption Electrospray Ionization High-Resolution Mass Spectrometry, Environmental Science & Technology, 48, 10993-11001, 10.1021/es5024674, 2014.
Toyota, K., Kanaya, Y., Takahashi, M., and Akimoto, H.: A box model study on photochemical interactions between VOCs and reactive halogen species in the marine boundary layer, Atmos Chem Phys, 4, 1961-1987, 2004.
van Pinxteren, D., Bruggemann, E., Gnauk, T., Iinuma, Y., Muller, K., Nowak, A., Achtert, P., Wiedensohler, A., and Herrmann, H.: Size- and time-resolved chemical particle characterization during CAREBeijing-2006: Different pollution regimes and diurnal profiles, J Geophys Res-Atmos, 114, 10.1029/2008JD010890, 2009.
Wang, X. K., Rossignol, S., Ma, Y., Yao, L., Wang, M. Y., Chen, J. M., George, C., and Wang, L.: Identification of particulate organosulfates in three megacities at the middle and lower reaches of the Yangtze River, Atmos. Chem. Phys. Discuss., 2015, 21415-21448, 10.5194/acpd-15-21415-2015, 2015.
Wayne, R. P., Barnes, I., Biggs, P., Burrows, J. P., Canosa-Mas, C. E., Hjorth, J., LeBras, G., Moortgat, G. K., Perner, D., Poulet, G., Restelli, G., and Sidebottom, H.: The Nitrate Radical: Physics, Chemistry, and the Atmosphere, Atmospheric Environment Part A - General Topics, 25, 1-203, 1991.
Willoughby, A. S., Wozniak, A. S., and Hatcher, P. G.: A molecular-level approach for characterizing water-insoluble components of ambient organic aerosol particulates using ultrahigh-resolution mass spectrometry, Atmos Chem Phys, 14, 10299-10314, 10.5194/acp-14-10299-2014, 2014.
Wozniak, A. S., Willoughby, A. S., Gurganus, S. C., and Hatcher, P. G.: Distinguishing molecular characteristics of aerosol water soluble organic matter from the 2011 trans-North Atlantic US GEOTRACES cruise, Atmos Chem Phys, 14, 8419-8434, 10.5194/acp-14-8419-2014, 2014.
Xiao, S., and Bertram, A. K.: Reactive uptake kinetics of NO_3 on multicomponent and multiphase organic mixtures containing unsaturated and saturated organics, Phys. Chem. Chem. Phys., 13, 6628-6636, 2011.
Xu, L., Guo, H., Boyd, C. M., Klein, M., Bougiatioti, A., Cerully, K. M., Hite, J. R., Isaacman-VanWertz, G., Kreisberg, N. M., Knote, C., Olson, K., Koss, A., Goldstein, A. H., Hering, S. V., de Gouw, J., Baumann, K., Lee, S.-H., Nenes, A., Weber, R. J., and Ng, N. L.: Effects of anthropogenic emissions on aerosol formation from isoprene

and monoterpenes in the southeastern United States, Proceedings of the National Academy of Sciences, 112, 37-42, 2015a.
Xu, L., Suresh, S., Guo, H., Weber, R. J., and Ng, N. L.: Aerosol characterization over the southeastern United States using high-resolution aerosol mass spectrometry: spatial and seasonal variation of aerosol composition and sources with a focus on organic nitrates, Atmos. Chem. Phys., 15, 7307-7336, 10.5194/acp-15-7307-2015, 2015b.
Yang, X. K., Wang, J. Q., and Wang, T. D.: Rate constants for the reactions of NO_3 center dot and SO_4 center dot- radicals with oxalic acid and oxalate anions in aqueous solution, Chinese Chem Lett, 15, 583-586, 2004.
Zellner, R., Herrmann, H., Exner, M., Jacobi, H.-W., Raabe, G., and Reese, A.: Formation and reactions of oxidants in the aqueous phase, in: Heterogeneous and Liquid Phase Processes, edited by: Warneck, P., Springer, 146–152, 1996.
Zhang, P., Sun, W., Li, N., Wang, Y., Shu, J., Yang, B., and Dong, L.: Effects of Humidity and NO_3 / N_2O_5 Ratio on the Heterogeneous Reaction of Fluoranthene and Pyrene with $N_2O_5/NO_3/NO_2$, Env. Sci. Tech., 48, 13130-13137, 10.1021/es504508v, 2014a.
Zhang, X., Cappa, C. D., Jathar, S. H., McVay, R. C., Ensberg, J. J., Kleeman, M. J., and Seinfeld, J. H.: Influence of vapor wall loss in laboratory chambers on yields of secondary organic aerosol, Proceedings of the National Academy of Sciences, 111, 5802-5807, 2014b.
Zhang, Y., Chapleski, R. C., Lu, J. W., Rockhold, T. H., Troya, D., and Morris, J. R.: Gas-surface reactions of nitrate radicals with vinyl-terminated self-assembled monolayers, Physical Chemistry Chemical Physics, 16, 16659-16670, 10.1039/C4CP01982B, 2014c.
Zhang, Y., and Morris, J. R.: Hydrogen Abstraction Probability in Reactions of Gas-Phase NO_3 with an OH-Functionalized Organic Surface, The Journal of Physical Chemistry C, 119, 14742-14747, 10.1021/acs.jpcc.5b00562, 2015.
Zhao, Y., Hallar, A. G., and Mazzoleni, L. R.: Atmospheric organic matter in clouds: exact masses and molecular formula identification using ultrahigh-resolution FT-ICR mass spectrometry, Atmos Chem Phys, 13, 12343-12362, 10.5194/acp-13-12343-2013, 2013.
Zhao, Z., Husainy, S., Stoudemayer, C. T., and Smith, G. D.: Reactive uptake of NO_3 radicals by unsaturated fatty acid particles, Physical Chemistry Chemical Physics, 13, 17809-17817, 2011.
